# Predicting the global potential distribution of *Bursaphelenchus xylophilus* using an ecological niche model: expansion trend and the main driving factors

**DOI:** 10.1186/s12862-024-02234-1

**Published:** 2024-04-17

**Authors:** Yang Xiao, Qiqiang Guo, Na Xie, Gangyi Yuan, Mengyun Liao, Qin Gui, Guijie Ding

**Affiliations:** Institute for Forest Resources & Environment of Guizhou, Key Laboratory of Forest Cultivation in Plateau Mountain of Guizhou Province, College of Forestry, Guizhou University, 550025 Guiyang, PR China

**Keywords:** *Bursaphelenchu xylophilus*, Climate change, Pine wilt disease, MaxEnt, Distribution

## Abstract

*Bursaphelenchus xylophilus* (Steiner&Buhrer) Nickle is a global quarantine pest that causes devastating mortality in pine species. The rapid and uncontrollable parasitic spread of this organism results in substantial economic losses to pine forests annually. In this study, we used the MaxEnt model and GIS software ArcGIS10.8 to predict the distribution of *B. xylophilus* based on collected distribution points and 19 environmental variables (with a correlation coefficient of|R| > 0.8) for the contemporary period (1970–2000), 2041–2060 (2050s), 2061–2080 (2070s), and 2081–2100 (2090s) under four shared socioeconomic pathways (SSPs). We conducted a comprehensive analysis of the key environmental factors affecting the geographical distribution of B. *xylophilus* and suitable distribution areas. Our results indicate that in current prediction maps *B. xylophilus* had potential suitable habitats in all continents except Antarctica, with East Asia being the region with the most highly suitable areas and the most serious epidemic area currently. Precipitation of the warmest quarter, temperature seasonality, precipitation of the wettest month, and maximum temperature of the warmest month were identified as key environmental variables that determine the distribution of *B. xylophilus*. Under future climatic conditions, the potential geographic distribution of *B. xylophilus* will expand relative to current conditions. In particular, under the SSP5-8.5 scenario in 2081–2100, suitable areas will expand to higher latitudes, and there will be significant changes in suitable areas in Europe, East Asia, and North America. These findings are crucial for future prevention and control management and monitoring.

**Supplementary Information:**

The online version contains supplementary material available at 10.1186/s12862-024-02234-1.

## Introduction

Global climate change is a critical crisis and a formidable challenge for humankind, with far-reaching impacts on natural ecosystems and human development. Research has shown that climate change is already a major threat to biodiversity and is expected to become a primary and immediate driver of biodiversity loss in the coming decades [1, 2]. In this context, numerous species are adapting to complicated and volatile environments through migration, including endangered plants and animals, as well as invasive pests. Furthermore, human disturbances are affecting climate factors, and the air temperature has risen by an average of 0.32 °F (0.18℃) per decade since 1981, with climate sensitivities ranging between 2.5℃ and 4℃ [3, 4]. Therefore, several researchers have reported the migration of pests and pathogens’ geographical distribution due to climate change [5].

The global climate change has caused an expansion in the geographic distribution of a majority of biological species, particularly insect pests [6–8], resulting in two major issues. Firstly, the disorderly spread of species distribution has led to biological invasion, which is a significant problem faced by humanity today, and is mainly caused by accelerated globalization, human disturbances, international trade, and tourism [9–11]. Secondly, interactions between forest trees and their parasites have become more complex, hindering the control effectiveness against harmful species. Thus, it is crucial to understand how climate change affects the survival range of insect pests to identify suitable areas for taking effective measures of biological control in the context of current and future climate change. Studies have reported the migration of the geographical distribution of pests and pathogens due to climate change, highlighting the need for effective management strategies to mitigate their impact on ecosystems and human well-being.

Modeling approaches for predicting species distributions based on species ecological niches are now commonly used [12]. In recent years, there has been growing interest in using ecological niche models (ENMs) to study the spatial occurrence patterns of invasive species. ENMs have proven to be effective in predicting species potential distributions and assessing extinction risks. These models use ecological niche theory to analyze known species distribution data and associated environmental variables. Models are constructed using algorithms to project results to different geographic spaces for potential distribution prediction [13, 14]. With the development of science and technology, a variety of ecological niche models based on different algorithms had been produced. For instance, BIOCLIM, GARP, CLIMEX, RF, MaxEnt have been extensively applied in various applications [15–19]. Among these, the MaxEnt model has clear advantages over other methods, including feature class selection, handling complex interactions between variables [20, 21]. The Maxent model, among various species distribution models, exhibits a high level of accuracy in predicting outcomes for species characterized by limited sample size, restricted geographical range, and constrained environmental tolerance [22]. The main emphasis lies in the characteristics of short duration, simplified operation, minimal sample size requirement, and superior performance. As a result, MaxEnt is widely used to predict the geographical distributions of endangered animals, plants, and invasive species colonization [7, 23–25].

*Bursaphelenchus xylophilus* (Steiner & Buhrer) Nickle, which is native to North America, is a serious pest that can harm dozens of pine species and a few non-pine conifer species. It spread to Asia and Europe from the 1970s to the 1990s [26, 27]. Once a tree is infected by *B. xylophilus*, Pine Wilt Disease (PWD) occurs and spreads to other individuals due to anthropogenic activities and climate change. This can cause a growing number of trees to wither and die within several months [28, 29]. According to the report, approximately 580,000 hm^2^ of pine forests in Japan were affected by this species, representing 28% of the total pine forest area in the country in 2000 [30]. PWD had caused the direct economic losses of about 4.2 billion yuan in China alone, the occurrence area was1,511,500 hm^2^ in 2022 [31]. Therefore, how to prevent and control PWD has become a major global forestry issue. At present, *B. xylophilus* has become a critical quarantine invasive species in the world, and PWD, known as the cancer of pine trees, is recognized as a great threat to forest ecosystems worldwide [32–34].

Pine forests are famous for being fast-growing and productive timber forests, which have important economic value and occupy a special position in the world timber production. Doubtlessly, the conservation of pine plants and the monitoring of *B. xylophilus* expansion are common tasks worldwide. Currently, the vast majority of researches about *B. xylophilus* have been carried on its physiology, ecology, and past distribution [35–37]. However, the detailed global distribution and the future spreading trends of *B. xylophilus* are still unclear [35, 38]. In other words, based on current and future climate conditions, the habitat suitability of *B. xylophilus* and environmental factors driving its global distribution are still not understood. We imported a lot of occurrence points data and used MaxEnt modeling to predict the suitable survival areas of *B. xylophilus* worldwide.

Our study aims to answer the following questions: (1) What is the current habitat suitability of *B. xylophilus*? (2) What are the main driving factors for the global expansion of *B. xylophilus* distribution? and (3) Will the habitat suitability for *B. xylophilus* contract or expand under future global climate change? Finally, our findings will help facilitate the development of effective control programs for *B. xylophilus*.

## Materials and methods

### Occurrence data of B. xylophilus

**Fig. 1:**
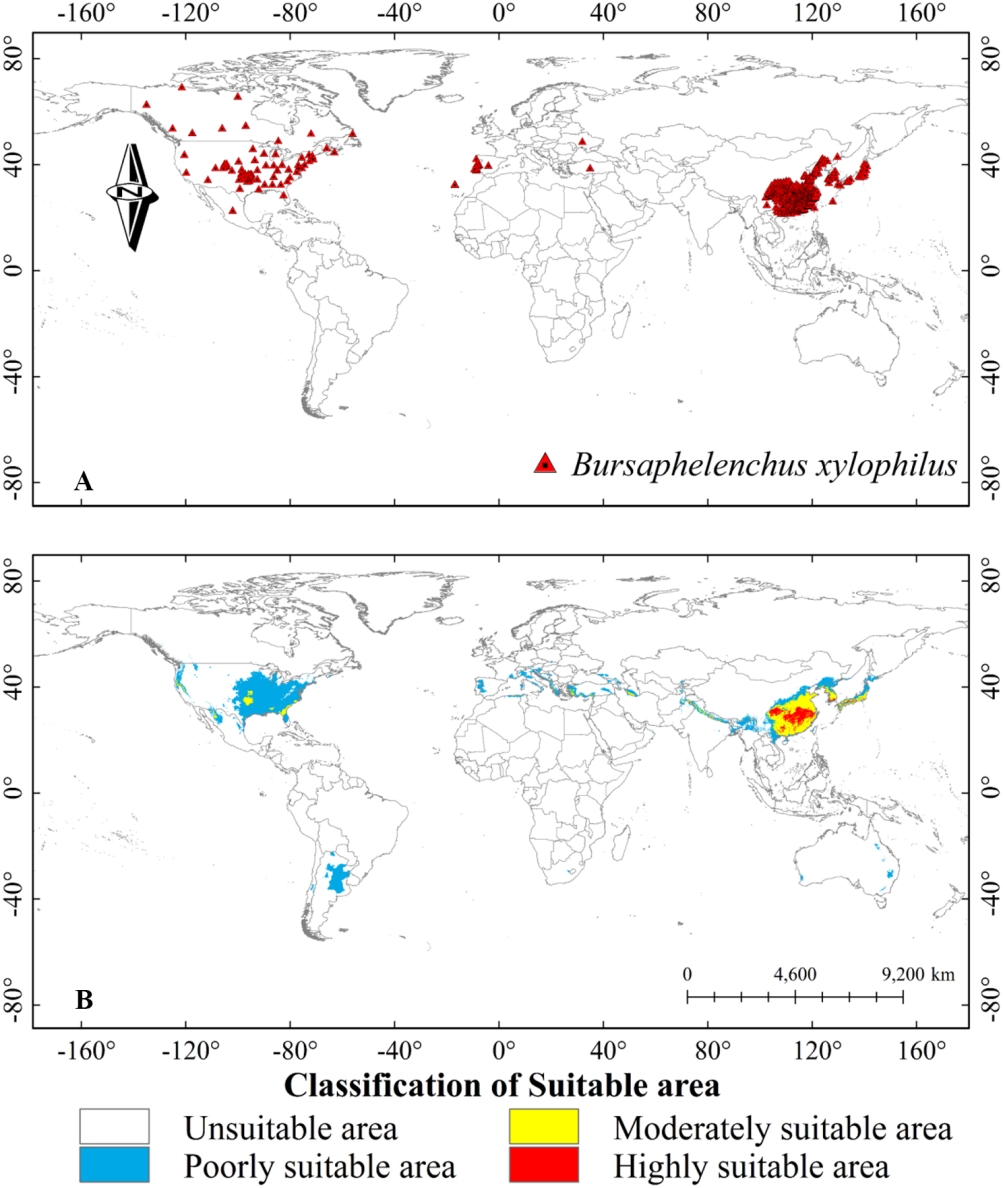
(**A**) Global geographic distribution of the occurrence points of *B. xylophilus*; (**B**) Predicted distribution of *B. xylophilus* suitable areas under current climatic conditions; *Note* In the horizontal direction, the negative value is the west longitude, and the positive value is the east longitude; In the vertical direction, negative values are south latitudes and positive values are north latitudes. The world map (1:460,000,000) was downloaded from the Google Earth Pro (version 7.0) (https://earth.google.com) for using as the base map, and the same is as following the Figs. 7, 8, 9, and 10

We obtained occurrence point data for *B. xylophilus* from multiple sources, including the Centre for Agriculture and Bioscience International (CABI), the Global Biodiversity Information Facility (GBIF) (the last time to browse CABI was November 13, 2022, and to browse GBIF was November 8, 2022), Announcement No. 6 of 2022 issued by the State Forestry and Grassland Administration of China, and publicly available studies [30, 39–41]. For locations mentioned in publicly available studies without latitude and longitude data, we used Google Earth (version 7.1) to obtain GPS coordinates. A total of 857 primary occurrence sites were collected from Asia, North America, and Europe. We opened the Trim duplicate occurrences function in ENMTools [42], then checked the grid cell and ran the result on the BIO01 raster layer (Any climate variable raster layer). Only one distribution point was retained in each 5 km × 5 km grid, thus reducing the influence of sampling bias and data redundancy on the prediction results. Finally, 844 distribution points were retained for model training and verification [43]. (Fig. 1-A, Table **S1**).

### Environmental variables

There was previous evidence that *B. xylophilus* were able to adapt to any forest, independent of climate and geography, as long as pine hosts and *Monochamus* vectors were available [44]. In North America, *B. xylophilus* was found from sub-boreal to sub-tropical pine forest [45]. It is enough to illustrate the strong environmental adaptability of *B. xylophilus*. In addition, environmental triggers such as higher temperatures, reduced rainfall, or mechanical damage to trees are thought to promote the growth of these deadly populations [46]. After learning the specificity of this species’ habitat, we did not eliminate any environmental variables in advance. Therefore, the default contribution bioclimatic factor prediction model was used in this study.

The 19 environment variables used were downloaded from WorldClim (http://www.worldclim.org, downloaded on 13th December 2022). The time range of current environmental data was 1970–2000. The time range of future environmental data was 2041–2100. It was divided into 3 periods: 2041–2060, 2061–2080 and 2081–2100. The Beijing Climate Center Climate System Model 2 Medium Resolution (BCC-CSM2-MR) [47] and four shared socio-economic paths were selected for future climate data. The four paths were SSP1-2.6 Sustainable Pathway, SSP2-4.5 Intermediate Pathway, SSP3-7.0 Regional Competitive Pathway, and SSP5-8.5 Fossil Fuel Pathway [48, 49]. All environment variables were downloaded in TIFF format. Then, we converted and processed the raw environmental data in ArcGIS 10.8 (https://www.arcgis.com/) using the SDM toolbox v2.5 (http://www.sdmtoolbox.org/) to obtain bioclimatic data in *. asc format that conforms to MaxEnt software (version 3.4.4), based on the distribution area of *B. xylophilus*. The aforementioned environmental variable raster layers were all characterized by a spatial resolution of 2.5 arc minutes and projected on the coordinate system WGS1984.

### Filter out the appropriate variables

To obtain a matrix of Pearson correlation coefficients among the 19 current (1970–2000) climate factors, we used the correlation function of ENM Tools v1.4 (Fig S1). We chose pearson correlation coefficient R as 0.8. When the absolute value of the correlation coefficient of two environmental variables was greater than 0.8 (|R|>0.8), we removed variables with small contributions and retained biologically important variables from the original model to reduce multicollinearity in environmental variables [50]. We added the obtained 844 valid occurrence points and 19 current climate factor data to MaxEnt software and ran it 4 times. After comparing the results of the percent contribution and Jackknife analysis, we needed to eliminate climate factors that contributed less than or equal to 0.5, and retain relatively important climate factors among the significantly correlated factors. Five climate factors were eventually obtained from 19 environmental variables to participate in the modeling (Table 1).

**Table 1:** The 5 environment variables used in the final MaxEnt model

Environmental variables	Description
BIO04	Temperature seasonality (standard deviation *100)
BIO05	Max temperature of warmest month (°C)
BIO13	Precipitation of wettest month (mm)
BIO14	Precipitation of driest month (mm)
BIO18	Precipitation of warmest quarter (mm)

### Model optimization

In this study, Feature Class (FC) and Regularization Multiplier (RM) are the two most key parameters that affect the results of Maxent model analysis [51, 52]. There were five types of Feature Classes, namely L-Linear features, Q-Quadratic features, P-Product features, T-Threshold features, H-Hinge features. Hinge features were introduced later than Threshold features and were used as a substitute rather than a complement to threshold features [53]. Therefore, this study ignored the latter. We set up 15 combinations (l, q, p, h, lq, lp, lh, qp, qh, ph, lqp, lqh, lph, qph, lqph) for model testing. Set 40 RM parameters in the range of 0.1-4 with an interval of 0.1. The model calibration, creation and evaluation were done in the “kuenm” program. This was an R package using MaxEnt as the modeling algorithm [54]. To optimizing these two parameters helped to reduce simulation over-fitting and complexity, thus significantly improving prediction accuracy. Finally, we have two criteria to choose the model for optimal calibration: statistically significant means that the omission rate was lower than the threshold (0.05) and the delta AICc value was not higher than 2 [54]. The model that satisfies both conditions will be considered as the optimally calibrated model.

### MaxEnt model construction

To begin with, we browsed the processed distribution points and bioclimatic data respectively in the MaxEnt interface. A subsample for the replicated run type was selected, with a random test percentage of 30% and 70% for the training and test set. To ensure sufficient time for the model to converge, the maximum iteration was set to 5000. The output format was set to Logistic with an output file type of asc to allow ArcGIS to recognize the prediction layer file. Then, the number of threads was set to 4, and the number of repetitions was 10. When the modeling was completed using MaxEnt software [51], the robust variables were determined by combining Jackknife analysis and assessing the contribution of environmental variables, resulting in the creation of a response curve. The accuracy of the simulation results was evaluated using the area under curve (AUC) of the receiver operating characteristic curve (ROC). AUC_TEST_ is generally thought not to suffer from the same overfitting problems as AUC_TRAIN_ because overfitting the model to the training data should not necessarily improve fit to independent test data [55]. Overfitting is when an existing model doesn’t generalize well from the original dataset to a new dataset. The criterion for overfitting is very simple: the model has very low errors on the training set (the original dataset) and very high errors on a new test set (the new dataset). The AUC value determined the accuracy of the model which ranged from 0 to 1. The larger the value, the higher the accuracy of the model prediction. When the value of AUC was less than or equal to 0.6, it indicated that the model performance failed and the predicted results were unreliable. Further, 0.6 to 0.7 means poor, 0.7 to 0.8 means Fair, 0.8 to 0.9 means Good, 0.9 to 1.0 means excellent prediction accuracy [19, 56].

### Hierarchical classification of species habitats and changes in spatial patterns

Based on the modeling results, ArcGIS 10.8 was used to visually present the potential distribution area forecast maps for the current period (1970–2000) and the future 2050s, 2070s, and 2090s, and to calculate the trend and area of suitable distribution regions. When creating the future 12 forecast distribution (3 periods x 4 paths), we browsed to the folder where we set the corresponding path and loaded to the " Projection layers directory/files”. For this study, we selected the maximum test sensitivity plus specificity (MTSPS) among all Logistic Thresholds generated by the MaxEnt model. In general, this was a commonly used and well-performing adaptive threshold that maximizes the sum of sensitivity and specificity [57]. The final Logistic Threshold was determined to be 0.1181 and was used to classify the suitable and unsuitable area. The probability (P) of *B. xylophilus* was set to range from 0 to 1 on the final suitability map. The potential distribution areas were divided into four classes on the global geographic map: highly suitable area (*P* > 0.6), moderately suitable area (0.4 < *P* ≤ 0.6), poorly suitable area (MTSPS < *P* ≤ 0.4), and unsuitable area (P ≤ MTSPS) [19, 58].

We used the “distribution changes between binary SDMs” tool in SDM toolbox v2.5 to create binary maps with MTSPS as the threshold. All the binary maps for each of the 12 climate change scenarios from 2041 to 2100 were compared with the binary maps for the current climate scenarios to clarify the future changes in the distribution of *B. xylophilus*.

## Results

### Optimal calibration model and model accuracy evaluation

The default parameters of MaxEnt fail to meet contemporary requirements, and although early developers extensively tested numerous combinatorial models, their primary emphasis was on predicting actual distributions rather than promoting post-construction transferability. Our optimization using the Kuenm package resulted in a total of 600 candidate models. We selected two models, both of which satisfy both of the previously mentioned criteria. In Table 2 it can be clearly observed that the value of Delta.AICc was zero when RM = 0.6 and FC = QPH. This combination had the lowest Delta.AICc compared to the other combinations. It was shown that when RM = 0.6 and FC = QPH, the complexity of the model and the degree of overfitting were reduced and the model accuracy was higher.

**Fig. 2:**
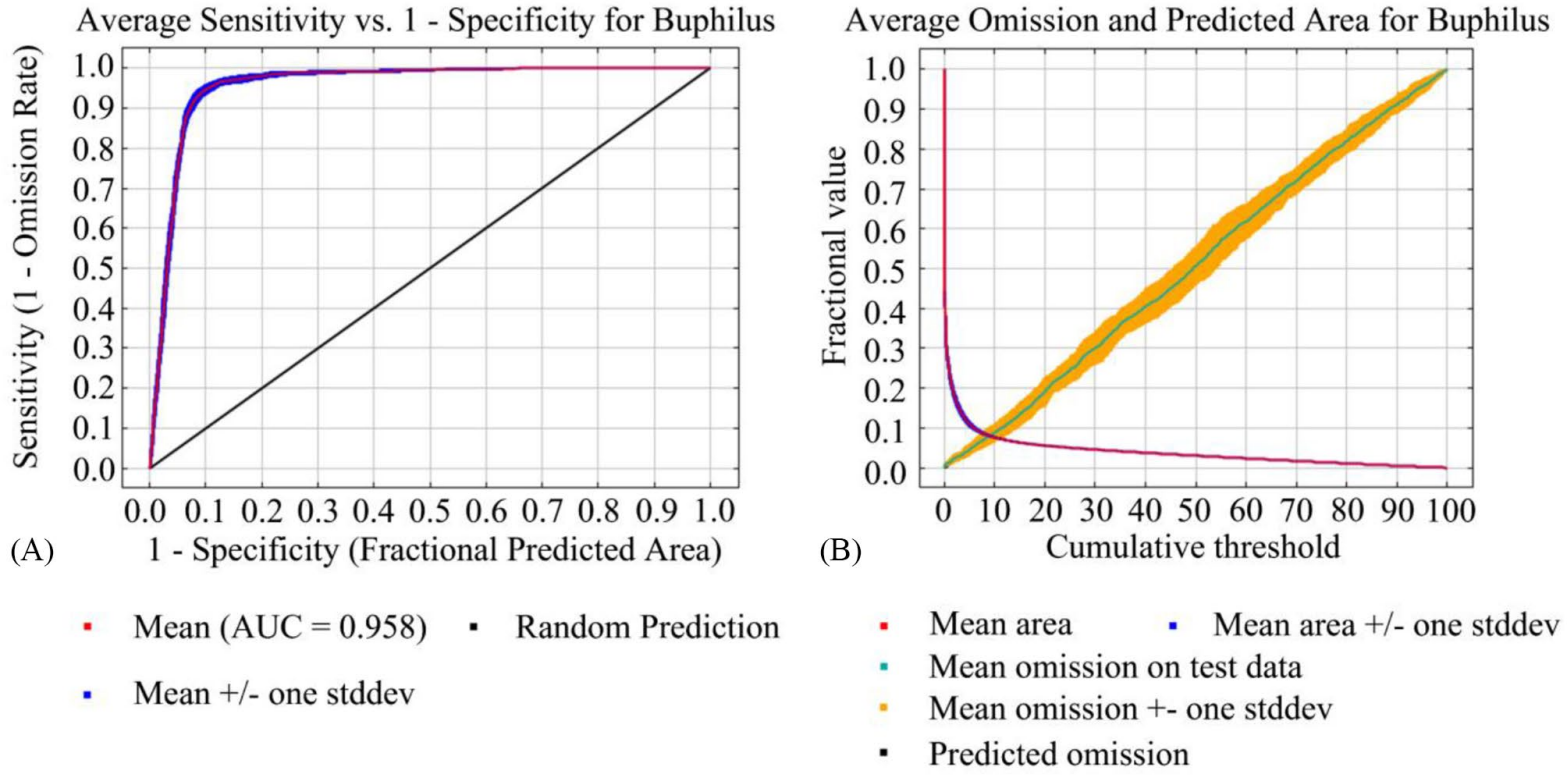
Accuracy analysis of the model evaluating the potential geographical distribution of *B. xylophilus*; (**A**): Receiver operating characteristic curve (ROC curve); (**B**): Omission rate

To prevent model overfitting, the model reliability was verified using AUC_DIFF_ =|AUC_TRAIN_ - AUC_TEST_| (0.0022). The smaller the value, the less overfitting the constructed model. The ROC curves and mean AUC values were used to assess the predicted distribution of *B. xylophilus*. The AUC value of the ultimate model was 0.958 (SD = 0.0037) (Fig. 2-A), indicating excellent model accuracy. As a result, the predicted distribution of *B. xylophilus* based on the MaxEnt model has excellent results for the projecting of potential distribution areas under current climate scenarios.

### The impact of environmental factors on the potential geographical distribution of B. xylophilus

**Fig. 3:**
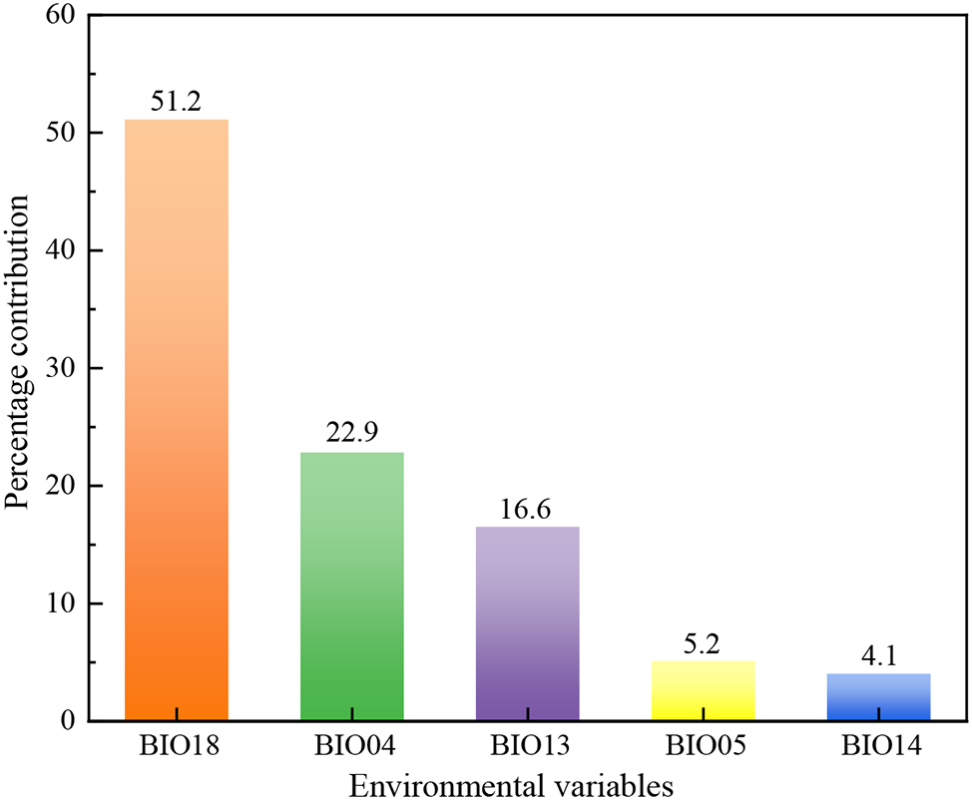
Environmental variables for modelling and corresponding contribution rates

**Fig. 4:**
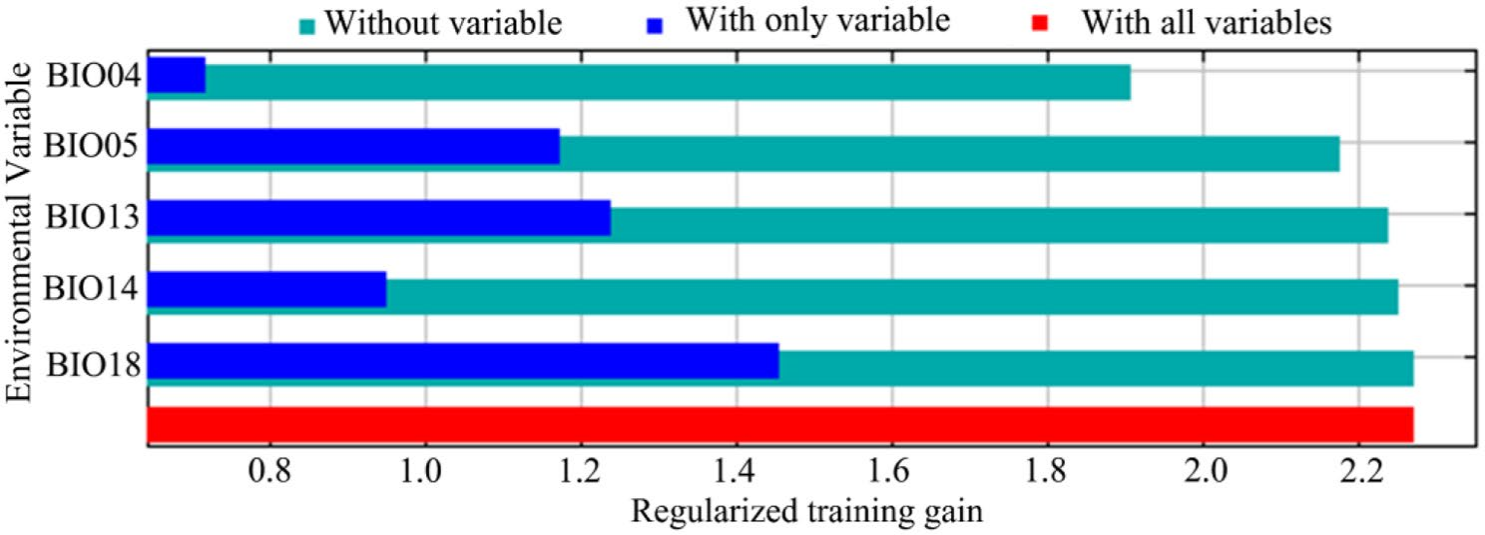
Jackknife analysis for evaluating the relative importance of major environmental variables for *B. xylophilus*

The modeling results demonstrated that among the five environmental variables used, BIO18 had the largest relative contribution (51.2%) to the potential distribution of *B. xylophilus* (Fig. 3). Following BIO18, BIO04 and BIO13 contributed 22.9% and 16.6%, respectively, making the cumulative contribution of these three variables 90.7%. The Jackknife analysis was also used to evaluate the importance of each variable. Figure 4 shows that the highest test gain was achieved when only BIO18 was considered, indicating that it provided the most critical information. The overall gain decreased significantly when BIO04 and BIO05 were ignored, demonstrating that these two environmental variables contained unique information not found in other environmental variables. Except for BIO04, the environmental factor with the lowest gain was BIO14 with the lowest contribution (4.1%). Hence, the key climatic variables impacting the geographical distribution of *B. xylophilus* were BIO18, BIO04, BIO13 and BIO05.

**Table 2:** Optimal model parameters based on kuenm package

Parameter settings	RM	FC	AUC ratio	Delta_AICc	OR	Partial ROC
Default	1.0	LQPH	1.914	14.630	0.047	0
Optimized	0.3	LQPH	1.906	1.644	0.047	0
Optimized	0.6	QPH	1.909	0	0.047	0

RM: Regularization multiplier; FC: Feature classes; AICc: Akaike information criterion correction; OR: Omission rate; L = Linear features, Q = Quadratic features, P = Product features, H = Hinge features

### Response of environmental variables

**Fig. 5:**
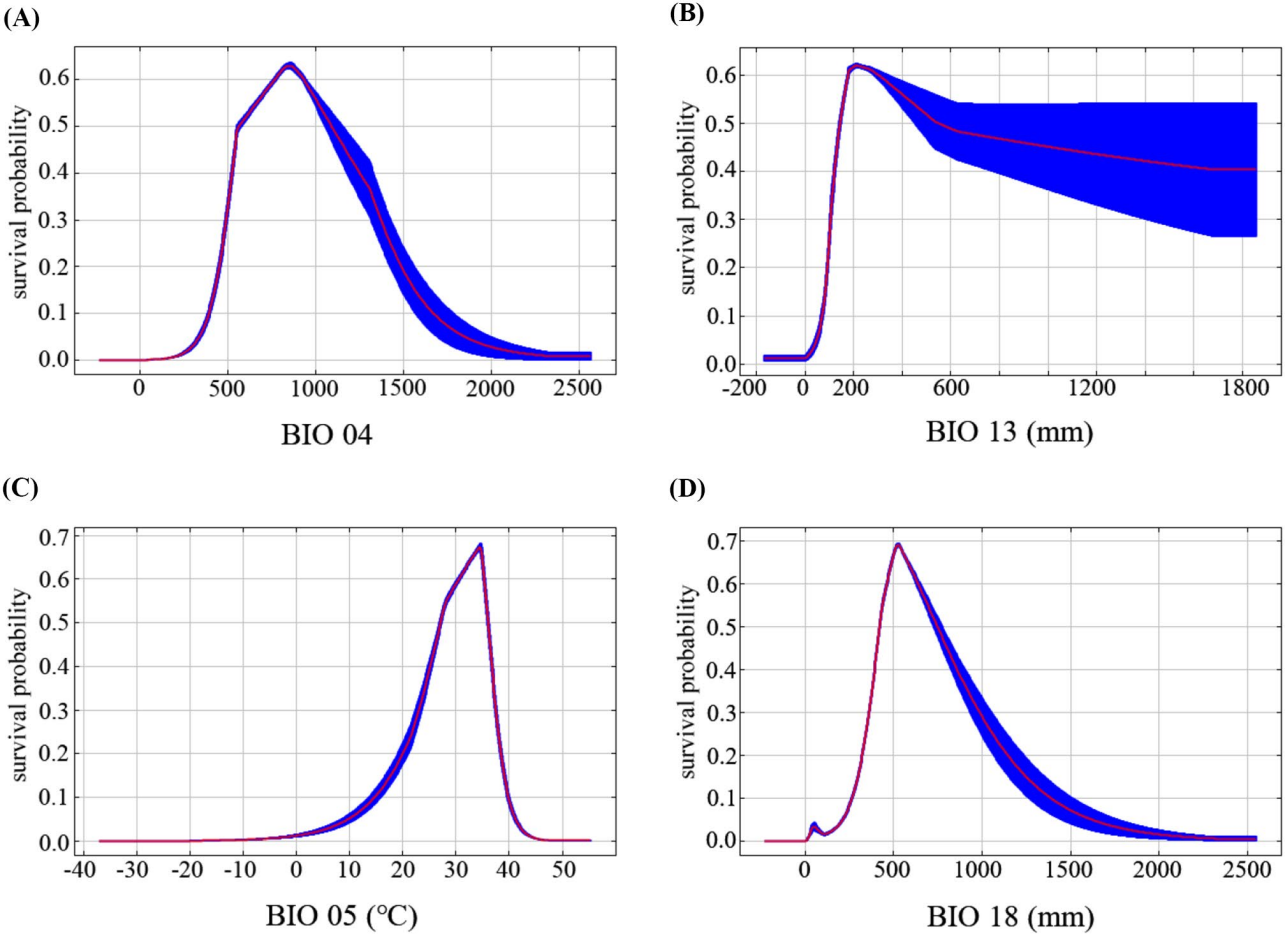
The relationships between optimum variables and survival probability of *B. xylophilus*. (**A**) Temperature seasonality; (**B**) Precipitation of the wettest month; (**C**) Max temperature of the warmest month; (**D**) Precipitation of the warmest quarter; Values shown were averaged over 10 replicate runs; Blue margins show ± SD

The response curve indicated that the survival probability of a species changes with the change of an environmental factor, and it could be used to explore the ecological relationship between various environmental factors and the distribution of *B. xylophilus*. They (Fig. 5-A, B, C, D) showed a dynamic relationship between the probability of the presence of *B. xylophilus* and the index values of the optimum factors. The suitable range of the probability of survival of *B. xylophilus* was determined by the probability greater than 0.5 [59]. For example, the precipitation of the warmest quarter (BIO18) had suitable probability of survival for *B. xylophilus* in regions where the optimal range was 450 mm to 750 mm. Similarly, when the precipitation range of the wettest month (BIO13) was 180–450 mm, the survival probability of this species was suitable. When the temperature seasonality (BIO14) ranges from 510 to 1150, the survival probability of this species was suitable. Finally, when the maximum temperature of the warmest month (BIO05) was 27℃∼ 36℃, the survival of *B. xylophilus* was suitable.

### Global distribution of potential suitable areas of B. xylophilus under current climate scenarios (1970–2000)

The current map showed that potential suitable areas for *B. xylophilus* are distributed to the varying degrees on all continents except Antarctica. The utilization of species’ known distribution data for predicting their potential distribution demonstrates a robust predictive efficacy. (Fig. 1-A, B). The majority of potentially suitable areas were located in the northern hemisphere, concentrated between 20° to 50° north latitude, and in the southern hemisphere, concentrated between 10° to 40° south latitude. Under the current climate conditions, the highly suitable area was mainly distributed in eastern Asia, and a few highly suitable areas were distributed in western North America and southern Asia. In Asia, highly suitable areas were mainly found in Japan, South Korea, China, India, Pakistan and Nepal. In North America, these areas were mainly on the West coast of the United States. Southern China, southern Japan, South Korea, southwest Turkey, northern Iran, North Korea, and northern Mexico had moderately suitable areas, mainly distributed in the vicinity of highly suitable areas. Poorly suitable areas were mainly distributed in central North America, southern South America, the Mediterranean coast of Europe, central and eastern Asia, and southeastern Oceania.

**Table 3:** Prediction of suitable area of *Bursaphelenchus xylophilus* under different climatic scenarios

Shared socio-economic pathways, SSPs| Decades		Predicted area (×10^6^ km^2^) and % of the corresponding current area							
		Total suitable area		Poorly suitable area		Moderately suitable area		Highly suitable area	
1970–2000		6.46		4.57		1.47		0.43	
SSP1-2.6	2050s	7.86	121.75%	5.75	126.00%	1.35	92.35%	0.76	177.23%
	2070s	8.22	127.32%	6.04	132.28%	1.31	89.40%	0.87	204.38%
	2090s	8.28	128.13%	5.93	129.75%	1.42	97.05%	0.93	217.50%
SSP2-4.5	2050s	8.10	125.33%	5.94	130.13%	1.23	83.65%	0.93	217.13%
	2070s	8.77	135.79%	6.64	145.34%	1.27	86.70%	0.86	202.23%
	2090s	8.32	128.76%	6.20	135.68%	1.14	77.50%	0.98	230.69%
SSP3-7.0	2050s	8.21	127.04%	6.07	132.96%	1.29	88.12%	0.84	197.40%
	2070s	8.40	130.10%	6.38	139.76%	1.37	93.47%	0.65	152.46%
	2090s	8.77	135.70%	6.87	150.43%	1.32	90.27%	0.57	134.16%
SSP5-8.5	2050s	8.82	136.60%	6.68	146.26%	1.22	82.90%	0.93	217.71%
	2070s	8.53	132.03%	6.51	142.60%	1.38	94.09%	0.64	149.20%
	2090s	9.12	141.23%	7.44	162.92%	1.19	80.93%	0.50	116.26%

**Fig. 6:**
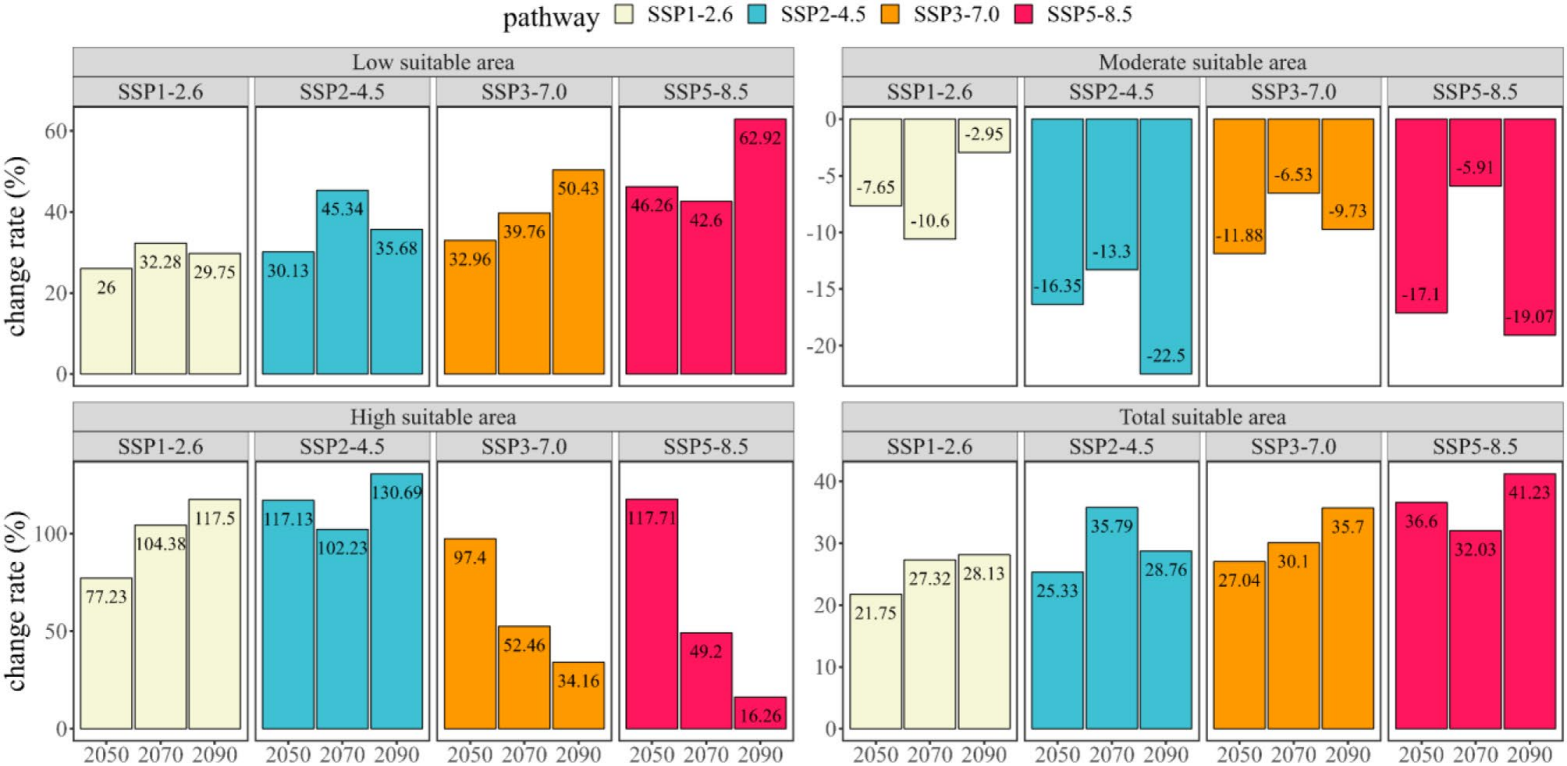
The rate of change of the predicted potential geographical distribution of *B. xylophilus* under future climate conditions compared with the potential geographical distribution under near current climate conditions

The predicted total area of potential suitable habitat for *B. xylophilus* was 6.46 × 10^6^ km^2^ under current climatic conditions, with the highly suitable area covering 0.43 × 10^6^ km^2^, accounting for 6.65% of the total suitable range. Moderately suitable areas covered 1.47 × 10^6^ km^2^, accounting for 22.75% of the total suitable area, and poorly suitable areas covered 4.57 × 10^6^ km^2^, accounting for 70.74% of the total suitable area (Table 3).

### Global distribution of potential suitable areas of B. xylophilus under future climate scenarios

For future projections (i.e. 2041–2060, 2061–2080 and 2081–2100) and CMIP6 climate scenarios (SSP1-2.6, SSP2-4.5, SSP3-7.0 and SSP5-8.5), a total of 12 climate scenarios were analyzed. Compared to the current climate scenario, the overall area of potential suitable areas increased, with a trend of migration towards higher latitudes Table 3; Figs. 9 and 10. Poorly and potential highly suitable areas increased, while potential moderately suitable areas decreased.

**Fig. 7:**
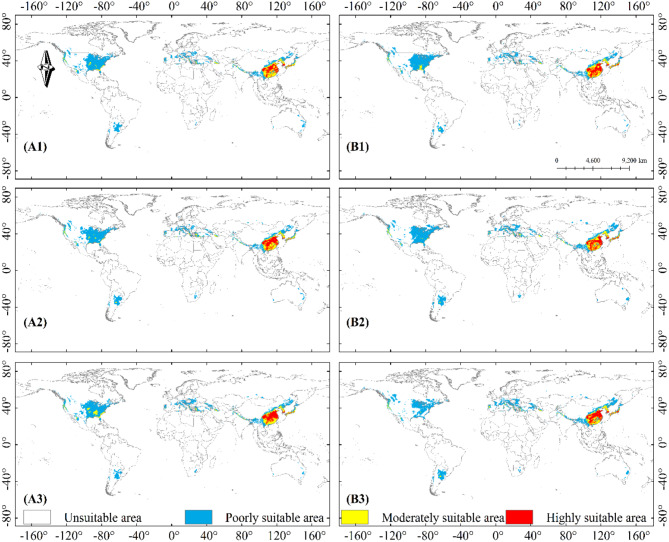
Potential distribution of *B. xylophilus* under future climate scenarios; Years: (1)2041–2060, (2)2061–2080, (3)2080–2100; Path: A: SSP1-2.6 and B: SSP2-4.5

The details are as follows: The combination of climate scenarios from 2081 to 2100 SSP5-8.5 anticipated the maximum potential total suitable area (9.12 × 10^6^ km^2^) for the future climate scenarios, an increase of 41.23% from the current climate conditions (Fig. 6). According to the 2041–2060 SSP1-2.6 climatic scenario combination, the minimum potential total suitable area (7.86 × 10^6^ km^2^) increased by 21.75% in contrast to the current climate conditions. The greatest estimated potential highly suitable area for 2081–2100 SSP2-4.5 was 0.98 × 10^6^ km^2^, which was 130.67% larger than the current climate conditions. The 2081–2100 SSP5-8.5 climatic scenario combination indicated the lowest potential highly suitable area (0.49 × 10^6^ km^2^), which grew by 16.26% compared to the current climate conditions. The maximum predicted potential moderately suitable area for 2081–2100 SSP1-2.6 was 1.42 × 10^6^ km^2^, which was 2.95% less than the current climate conditions. The minimum predicted potential moderately suitable area for 2081–2100 SSP2-4.5 was 1.14 × 10^6^ km^2^, which was 22.5% less than for the current climate conditions. The combination of climate scenarios from 2081 to 2100 SSP5-8.5 anticipated the maximum potential poorly suitable area (7.44 × 10^6^ km^2^) for the future climate scenarios, an increase of 62.92% from the current climate conditions. The combination of climate scenarios from 2041 to 2060 SSP1-2.6 anticipated the minimum potential poorly suitable area (1.14 × 10^6^ km^2^) for the future climate scenarios, an increase of 26% from the current climate conditions.

**Fig. 8:**
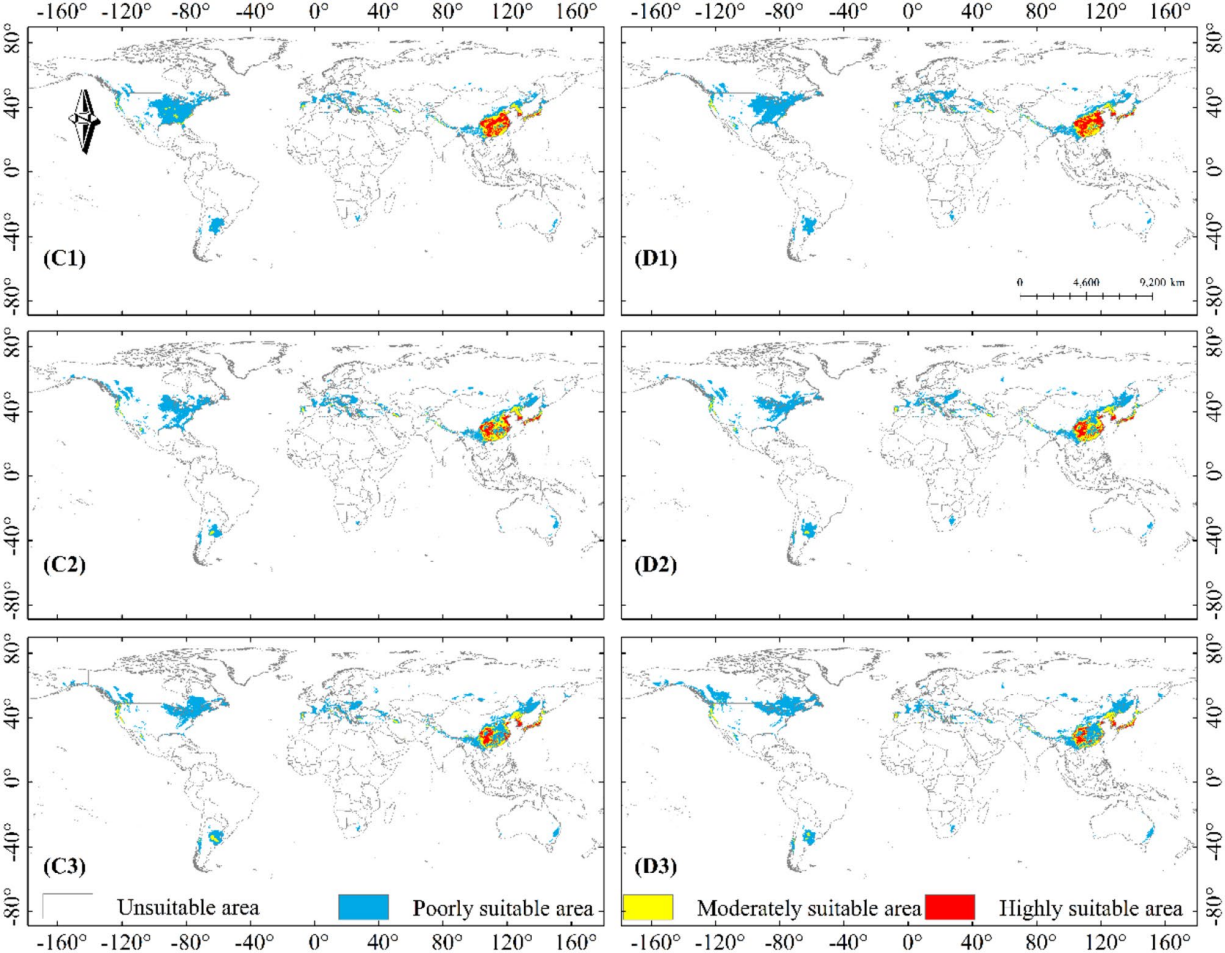
Potential distribution of *B. xylophilus* under future climate scenarios; Years: (1)2041–2060, (2)2061–2080, (3)2080–2100; Path: C: SSP3-7.0 and D: SSP5-8.5

The projections showed similar changes in the projected distribution areas for SSP1-2.6 and SSP2-4.5 compared to the current climate scenario. Significant expansion of potential highly suitable area could be observed in parts of Chinese mainland, Japan and Korea in 2041–2100 in SSP1-2.6 and SSP2-4.5 (Fig. 7-A1, A2, A3, B1, B2, B3). With the increase of concentration path and the passage of time, the coverage of each kind of suitable areas changed correspondingly. The expansion of the potential poorly suitable area to high latitudes continued in 2041–2060 SSP3-7.0 and SSP5-8.5 climate scenarios, with changes in the potential highly suitable area occurring in East Asia at any rate (Fig. 8-C1, D1). The potential poorly suitable areas began to appear within the potential highly suitable areas in East Asia, and the range of potential suitable areas expanded to higher latitudes; potential poorly suitable areas in the contiguous United States and Europe expanded northward, and the range of potential moderately suitable areas increased in 2061–2100 in SSP 3–7.0 and SSP 5-8.5 (Fig. 8-C2, C3, D2, D3).

### Spatial distribution trends of B. xylophilus in the future

**Fig. 9:**
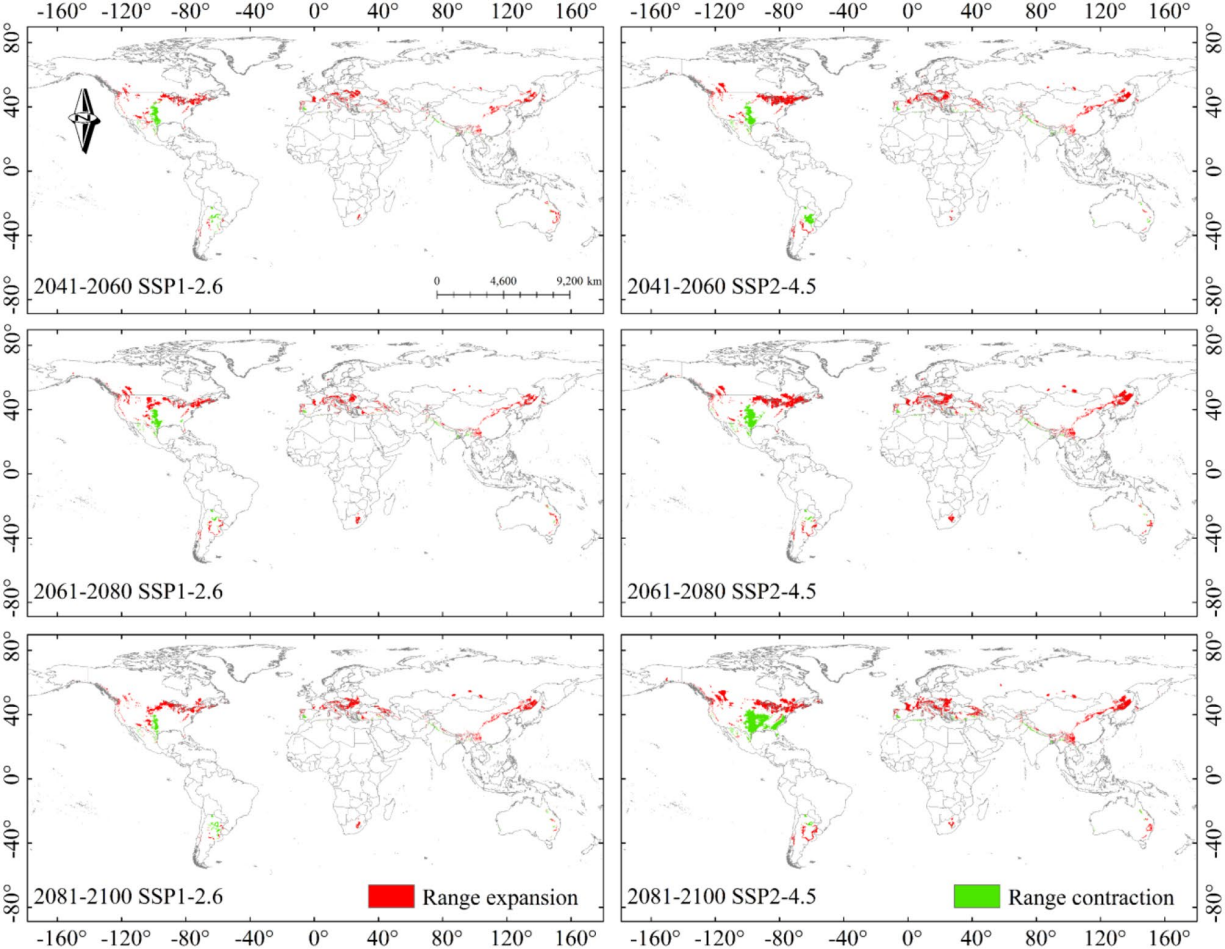
Potential range expansion/contraction of *B. xylophilus* suitable areas under SSP1-2.6 and SSP2-4.5 climate scenarios

**Fig. 10:**
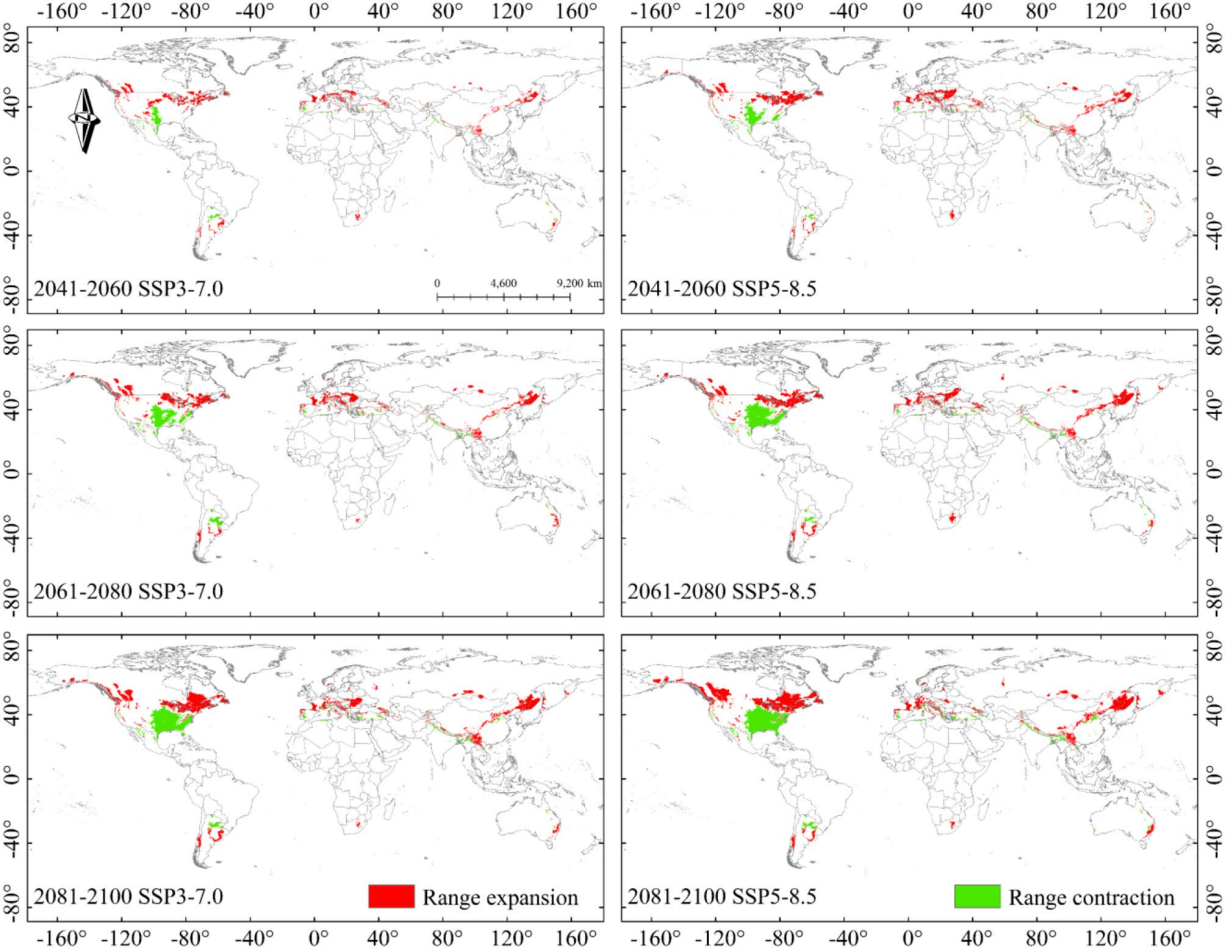
Potential range expansion/contraction of *B. xylophilus* suitable areas under SSP3-7.0 and SSP5-8.5 climate scenarios

We showed the specific location of the increase and loss areas in the map (Range expansion and Range contraction), which was helpful for the local regulatory authorities to take reasonable steps for prevention and control of the occurrence of *B. xylophilus*. Figures 9 and 10 illustrates the changes in the potential global distribution of *B. xylophilus* under the 12 SSP scenarios of CMIP6 from 2041 to 2100. The results indicated that the range of increasing and contracting areas was approximately the same under the future scenario, and the trend of change was to expand to higher latitudes and to contract northward in the low latitude suitable areas of the distribution. In the Americas, the increasing region includes mainly the area around the Great Lakes in the United States, the southeast and southwest regions of Canada, and parts of Argentina and Chile; in Europe, the increase was mainly concentrated in the central part of it; in Asia, the increase was mainly concentrated in East Asia, with a small expansion into southern Russia; in Oceania and Africa, the increase area expanded to parts of Australia and South Africa. The contraction area included mainly the central part of the United States, parts of Mexico, and the Mediterranean coast of Europe. As the SSP level increased, this expansion became more and more systematic.

## Discussion

### Significant factors which impact the potential distribution of B. xylophilus

Natural factors play an irreplaceable role in the distribution of pests and diseases in forests. The climate usually influences the distribution pattern of *B. xylophilus* on a large scale, such as the warmest monthly mean temperature above 20 °C was a high incidence area for PWD [60]. In our study, we investigated the survival temperature range of *B. xylophilus* based on the maximum temperature of the warmest month. The survival temperature of *B. xylophilus* was between 23 ℃ and 37℃, explaining that almost no *B. xylophilus* appeared in the high latitudes and elevations area under the current climate conditions [61]. Unquestionably, the reason is that the temperature is not sufficient for the *B. xylophilus* to survive.

The development and reproduction of *B. xylophilus* are not only influenced by thermal conditions but also by precipitation disturbances [60, 62]. Our study applied MaxEnt modeling and the climate variable that contributed most to the survival probability of *B. xylophilus* was BIO18. From the response curve, it can be observed that a low or excessive precipitation can have a large impact on its survival probability. The optimum rainfall range for *B. xylophilus* was 450 mm to 750 mm (BIO18). In some cases, rainfall-rich areas do facilitate the spread of pests and diseases on forest systems [60]. The survival of *B. xylophilus* exhibited an initial increase followed by a subsequent decrease with increasing precipitation, suggesting that optimal levels of precipitation are conducive to its survival. Precipitation directly determines the moisture content of the air and soil. Research indicated that the occurrence of heavy rainfall in specific regions during a particular time period led to an elevation in humidity levels, thereby facilitating the proliferation of fungus *Beauveria bassiana*. As the natural enemy of *B. xylophilus*, *Beauveria bassiana* had a significant inhibitory effect on the reproduction of *B. xylophilus* [29]. In addition, the survival and movement of *B. xylophilus* had been reported to decrease with the increase of wood moisture content [63]. Thus, this was further evidence that high temperature and dry climatic conditions were more suitable for the spread of PWD [64, 65].

### Reliability of simulation results

In the omission rate curve and the ROC curve of this model, the sample omission rate and the predicted omission rate basically matched and the maximum entropy model had a low AUC_DIFF_ value (Fig. 2-A, B). These results all indicated that the model predicts results with high accuracy and the low spatial correlation of modeled data. The results of Wisz et al. [20] indicated that the MaxEnt model, compared to other models, performs well, when both with more and less sample distribution points. Hence, the present study predicted that the current range of PWD suitable areas were generally consistent with those predicted by Ikegami and Jenkins [35]. The difference between them was the size of the range of suitable habitats in Europe. On the one hand, the reason may be that the version of the bioclimate variable downloaded was different; the previous studies (Ikegami and Jenkins) used outdated data, while this study used updated data. The completeness and accuracy of climate information have an important impact on the prediction effectiveness of the model. With the increasing accuracy of the model, it also facilitated the updating of the climate data. Our data came from WorldClim v2.1, which was much more progressive than the previous database (WorldClim v2.0, downloaded on 27th March 2014). On the other hand, it was due to the different ranges and densities of the collected *B. xylophilus* occurrence points. In this study, the occurrence of *B. xylophilus* in Canada, Mexico, Ukraine, and Turkey was added compared with previous studies (Ikegami and Jenkins), which made the range larger and more applicable to the prediction of potential distribution areas of *B. xylophilus*. In contrast, the occurrence points which we collected were mostly from East Asia, and the sample points were not homogeneous, resulting in a narrow range of ecological niche simulations and poor results for suitable habitats [66]. Consequently, there are discrepancies in the suitable habitat areas of the two predicted results.

### Future variation in potential suitable areas for B. xylophilus

When comparing the current and future potential suitable distribution areas of *B. xylophilus*, we found that the range of suitable areas tended to shift or expand towards higher latitudes under all 12 scenarios we examined. The higher the level of socio-economic complexity and radiation, the greater the extent of suitable area transfer and fragmentation (Figs. 9 and 10). Half of the species evaluated by the Intergovernmental Panel on Climate Change (IPCC) in its Sixth Assessment Report (AR6) displayed a trend towards migrating to polar or higher altitude regions, with some irreversible or near-irreversible effects already taking place [67, 68]. This highlights the increasing risk of climate change and the spread of insects to areas that are currently unsuitable under the present climate conditions [7, 19, 56].

The total suitable area was the largest and varies especially under the 2081–2100 SSP5-8.5 climate scenario. The range of potential suitable areas for *B. xylophilus* extended from the Mediterranean region to central and northern Europe in this climate scenario. In Asia, the unsuitable areas became poorly suitable areas, such as: southern Siberia, northeastern China, and the Russian Sakhalin Islands. In North America, they migrated to the northeast and northwest, respectively. In sum, all of the above is consistent with existing understanding of species responses to climate change. However, the SSP5-8.5 climate scenario will be a pollution type of development, and human development under this climate scenario will be not appropriate [48, 69].

SSP2 is more in line with the plausible future developments than the SSP5 pathway. It presented a “Middle of the Road” scenario, where socio-economic factors followed their historical trends with no significant changes, slowly progressed in sustainability, and uneven patterns of development and income growth [48]. Under the SSP2-4.5 scenario, the range of low and moderate suitable areas in central and southern Europe continues to expand over time, which indicated that in the future, some European countries will be suitable for the survival of *B. xylophilus* [27, 70]. So, to control the spread of PWD, the quarantine department has to implement an early control and an early treatment program to prevent the disease. Meanwhile, since human knowledge of the climate system is limited, the above climate scenarios are only possible future trends and directions of change, with a great deal of uncertainty [19]. However, the application of scenarios has important implications for the study of global climate change. These scenarios help us to understand the long-term implications of short-term decisions and to discuss alternative futures when the future is not at all certain [48].

### Research deficiencies and suggestions for prevention and control

While ENM approaches enhance our understanding of current and future risk areas for *B. xylophilus* colonization, which is helpful for understanding PWD risk, additional things need to be considered, such as PWD being the consequence of multiple factors acting in combination, thus considering the only temperature and precipitation is not enough. Matsuhashi et al. [71] evaluated the risk of PWD by establishing a non-uniform Poisson point process simulation and concluded that low elevation areas were high risk areas. Park et al. [72] considered that the slope had a significant effect on the occurrence and spread of PWD. It had also been suggested that the contribution of altitude was the largest of the numerous influencing factors, and that the distribution of suitable probability changed with altitude [73]. Moreover, human dynamics were direct and indirect drivers of biological invasions, and the spread of *B. xylophilus* was associated with the worldwide trade of forest products [74, 75]. All of the above illustrates that climate variables were not the only determinants of the occurrence of PWD. The impact factors used in this study were restricted to only 19 climatic variables. It will also be necessary to consider altitude, slope, and human dynamics in the future. Additionally, the carrier and transmitter of *B. xylophilus*, *Monochamus* spp, had an important effect on the occurrence of the pest disease. The spread and diffusion of *B. xylophilus* are closely related to vector insects [76]. Utilizing the characteristic of vector insects emerging from infected trees, *B. xylophilus* was able to actively attach itself to the surface or interior of the vector insects during a specific period, and was transmitted to healthy trees along with the vector insects [77]. Vector insects played a crucial role in the transmission chain of PWD. By effectively controlling and eradicating these insects, we were able to effectively break the transmission cycle, which was a widely recognized and highly effective measure for preventing and treating this destructive disease, both domestically and internationally. Control of the PWD will depend on control of its vector. However, this factor was not considered due to difficulties in obtaining data.

Our predictions indicated that there will be large areas suitable for *B. xylophilus* reproduction in the future. The most significant variations in the potential distribution areas were found in East Asia, Europe, parts of North Asia, and North America. Notably, in parts of central Europe and Argentina, where *B. xylophilus* has never been seen before, it is recommended that planning for cutting and replacing pine forests with other species over the next 30 years could prevent the invasion of *B. xylophilus* at its source. For example, it might be useful for the relevant authorities to choose disease-resistant pine seedlings bred in Japan instead of native pine forest [78, 79]. In light of the wide distribution of *B. xylophilus* in East Asia and the high requirements for monitoring accuracy, the local government could use the PWD multi-platform remote sensing with satellite and UAV regional measurements and manual ground verification as the main line for real-time monitoring [71, 80]. In order to keep track of *B. xylophilus* incidence area and make reasonable control decisions. Another study found by simulating the spread of PWD with cellular automata that *B. xylophilus* were more adapted to colder temperature areas [81]. As a result, the control of high altitude and low temperature areas predicted by the model cannot be ignored in the future prevention and control of *B. xylophilus*.

## Conclusion

The aim of this study was to investigate the distribution of potential suitable areas for *B. xylophilus* and to reveal the response of different impact factors. We employed a maximum entropy model and identified Precipitation of the warmest quarter, Temperature seasonality, Precipitation of the wettest month, and Max temperature of the warmest month as crucial climatic factors affecting the distribution of *B. xylophilus*. Our projections for future climate change scenarios indicated a gradual expansion of potentially suitable areas to higher latitudes and colder regions. The suitable area in East Asia, Europe and North America will change significantly. In particular, the total suitable area reached the maximum under the 2081–2100 SSP5-8.5 scenario, and the results had important reference significance for future prevention and control management and monitoring. Since the variables used in this study are bioclimatic variables only, in the future, it will be necessary to add soil, altitude, human activities and host impact factors. With more mature data, models and technical conditions, the prediction of future distribution of *B. xylophilus* will be more accurate.

### Electronic supplementary material

Below is the link to the electronic supplementary material.

Supplementary Material 1

## Data Availability

The datasets generated in this study are available from the corresponding author on reasonable request.
