# Supplementary material for: Predicting the global potential distribution of Bursaphelenchus xylophilus using an ecological niche model: expansion trend and the main driving factors
Source: BMC Ecol Evol. 2024 Apr 17;24:48. doi: 10.1186/s12862-024-02234-1 (PMC11022495; doi:10.1186/s12862-024-02234-1)
Supplement: Supplementary file 1 — Supplementary Material 1 [file 12862_2024_2234_MOESM1_ESM.docx]

**Predicting the global potential distribution of *Bursaphelenchus xylophilus* using an ecological niche model: expansion trend and the main driving factors**

**Appendix. Supplementary Tables and Figures**

**Description of FC and RM**

Feature Class (FC) and Regularization Multiplier (RM) are the two most key parameters that affect the results of MaxEnt model analysis.

1. FC corresponds to different environmental variables, and environmental variables are transformed mathematically, so that MaxEnt can use complex mathematical relationships to speculate on the response of species to environmental factors.

2. RM is a new constraint added to the model based on FC, and the simulation of the model’s response curve is adjusted by changing the numerical value.

The help documentation in MaxEnt software (version 3.4.4) also have detailed instructions on how to select FC and RM.

**Table S1** Global records of *Bursaphelenchus xylophilus* with coordinates.

| SPECIES | LONGITUDE | | LATITUDE |
| --- | --- | --- | --- |
| *Bursaphelenchus xylophilus* | | -100.063 | 66.0208 |
| *Bursaphelenchus xylophilus* | -102.021 | | 22.9792 |
| *Bursaphelenchus xylophilus* | -102.313 | | 38.0625 |
| *Bursaphelenchus xylophilus* | -104.229 | | 39.7292 |
| *Bursaphelenchus xylophilus* | -104.313 | | 40.6458 |
| *Bursaphelenchus xylophilus* | -104.854 | | 39.3958 |
| *Bursaphelenchus xylophilus* | -105.021 | | 39.7708 |
| *Bursaphelenchus xylophilus* | -105.063 | | 40.6042 |
| *Bursaphelenchus xylophilus* | -105.229 | | 39.7708 |
| *Bursaphelenchus xylophilus* | -105.271 | | 40.0208 |
| *Bursaphelenchus xylophilus* | -105.521 | | 39.0208 |
| *Bursaphelenchus xylophilus* | -106.021 | | 54.0208 |
| *Bursaphelenchus xylophilus* | -108.563 | | 39.0625 |
| *Bursaphelenchus xylophilus* | -111.521 | | 34.5208 |
| *Bursaphelenchus xylophilus* | -117.479 | | 52.2708 |
| *Bursaphelenchus xylophilus* | -119.771 | | 37.2708 |
| *Bursaphelenchus xylophilus* | -120.521 | | 44.0208 |
| *Bursaphelenchus xylophilus* | -121.521 | | 69.5208 |
| *Bursaphelenchus xylophilus* | -125.021 | | 53.9792 |
| *Bursaphelenchus xylophilus* | -135.021 | | 62.9792 |
| *Bursaphelenchus xylophilus* | -16.7708 | | 32.6875 |
| *Bursaphelenchus xylophilus* | -16.9375 | | 32.7708 |
| *Bursaphelenchus xylophilus* | -4.0208 | | 39.9792 |
| *Bursaphelenchus xylophilus* | -56.0208 | | 51.9792 |
| *Bursaphelenchus xylophilus* | -62.9792 | | 45.0208 |
| *Bursaphelenchus xylophilus* | -65.9792 | | 46.5208 |
| *Bursaphelenchus xylophilus* | -7.4792 | | 40.1458 |
| *Bursaphelenchus xylophilus* | -7.9375 | | 40.0625 |
| *Bursaphelenchus xylophilus* | -71.1042 | | 42.3542 |
| *Bursaphelenchus xylophilus* | -71.4792 | | 43.6875 |
| *Bursaphelenchus xylophilus* | -71.9792 | | 52.0208 |
| *Bursaphelenchus xylophilus* | -72.6458 | | 41.6875 |
| *Bursaphelenchus xylophilus* | -72.7292 | | 44.0208 |
| *Bursaphelenchus xylophilus* | -74.4792 | | 40.1875 |
| *Bursaphelenchus xylophilus* | -75.4792 | | 39.0208 |
| *Bursaphelenchus xylophilus* | -75.4792 | | 43.0208 |
| *Bursaphelenchus xylophilus* | -76.7708 | | 39.0208 |
| *Bursaphelenchus xylophilus* | -76.8958 | | 40.2708 |
| *Bursaphelenchus xylophilus* | -77.4375 | | 37.5625 |
| *Bursaphelenchus xylophilus* | -8.1458 | | 39.6875 |
| *Bursaphelenchus xylophilus* | -8.2292 | | 40.1042 |
| *Bursaphelenchus xylophilus* | -8.3125 | | 39.6875 |
| *Bursaphelenchus xylophilus* | -8.3125 | | 40.1042 |
| *Bursaphelenchus xylophilus* | -8.4375 | | 40.3958 |
| *Bursaphelenchus xylophilus* | -8.5208 | | 38.3542 |
| *Bursaphelenchus xylophilus* | -8.5625 | | 38.1875 |
| *Bursaphelenchus xylophilus* | -8.5625 | | 38.9792 |
| *Bursaphelenchus xylophilus* | -8.6458 | | 42.4375 |
| *Bursaphelenchus xylophilus* | -80.0208 | | 35.5208 |
| *Bursaphelenchus xylophilus* | -80.5208 | | 38.5208 |
| *Bursaphelenchus xylophilus* | -81.0208 | | 34.0208 |
| *Bursaphelenchus xylophilus* | -82.5208 | | 28.7708 |
| *Bursaphelenchus xylophilus* | -83.0208 | | 40.2708 |
| *Bursaphelenchus xylophilus* | -83.5208 | | 32.7708 |
| *Bursaphelenchus xylophilus* | -84.4792 | | 49.2708 |
| *Bursaphelenchus xylophilus* | -84.8958 | | 38.1875 |
| *Bursaphelenchus xylophilus* | -85.5208 | | 44.2708 |
| *Bursaphelenchus xylophilus* | -86.2708 | | 35.7708 |
| *Bursaphelenchus xylophilus* | -86.2708 | | 40.0208 |
| *Bursaphelenchus xylophilus* | -86.7708 | | 32.7708 |
| *Bursaphelenchus xylophilus* | -89.2708 | | 40.0208 |
| *Bursaphelenchus xylophilus* | -89.7708 | | 32.7708 |
| *Bursaphelenchus xylophilus* | -9.3125 | | 38.6875 |
| *Bursaphelenchus xylophilus* | -90.0208 | | 44.5208 |
| *Bursaphelenchus xylophilus* | -92.0208 | | 31.0208 |
| *Bursaphelenchus xylophilus* | -92.5208 | | 34.7708 |
| *Bursaphelenchus xylophilus* | -92.5208 | | 38.2708 |
| *Bursaphelenchus xylophilus* | -93.5208 | | 42.0208 |
| *Bursaphelenchus xylophilus* | -94.2708 | | 46.2708 |
| *Bursaphelenchus xylophilus* | -94.7708 | | 36.6042 |
| *Bursaphelenchus xylophilus* | -94.9792 | | 35.9375 |
| *Bursaphelenchus xylophilus* | -95.0625 | | 34.7708 |
| *Bursaphelenchus xylophilus* | -95.1458 | | 36.6458 |
| *Bursaphelenchus xylophilus* | -95.2708 | | 36.4375 |
| *Bursaphelenchus xylophilus* | -95.4792 | | 35.6042 |
| *Bursaphelenchus xylophilus* | -95.5208 | | 34.0208 |
| *Bursaphelenchus xylophilus* | -95.5208 | | 36.1458 |
| *Bursaphelenchus xylophilus* | -95.9375 | | 36.8125 |
| *Bursaphelenchus xylophilus* | -95.9792 | | 36.1458 |
| *Bursaphelenchus xylophilus* | -96.3958 | | 35.0625 |
| *Bursaphelenchus xylophilus* | -96.4375 | | 33.9375 |
| *Bursaphelenchus xylophilus* | -96.4792 | | 35.1458 |
| *Bursaphelenchus xylophilus* | -96.6042 | | 34.8958 |
| *Bursaphelenchus xylophilus* | -96.8958 | | 35.6875 |
| *Bursaphelenchus xylophilus* | -96.9375 | | 35.3542 |
| *Bursaphelenchus xylophilus* | -97.0208 | | 55.0208 |
| *Bursaphelenchus xylophilus* | -97.0625 | | 36.1042 |
| *Bursaphelenchus xylophilus* | -97.1875 | | 36.4792 |
| *Bursaphelenchus xylophilus* | -97.2708 | | 36.8125 |
| *Bursaphelenchus xylophilus* | -97.3542 | | 35.0208 |
| *Bursaphelenchus xylophilus* | -97.3958 | | 34.8125 |
| *Bursaphelenchus xylophilus* | -97.4375 | | 35.2292 |
| *Bursaphelenchus xylophilus* | -97.5208 | | 35.4792 |
| *Bursaphelenchus xylophilus* | -97.6042 | | 36.1458 |
| *Bursaphelenchus xylophilus* | -97.7292 | | 36.5625 |
| *Bursaphelenchus xylophilus* | -97.7708 | | 35.5625 |
| *Bursaphelenchus xylophilus* | -98.3542 | | 34.8958 |
| *Bursaphelenchus xylophilus* | -98.5208 | | 38.5208 |
| *Bursaphelenchus xylophilus* | -98.6875 | | 36.8125 |
| *Bursaphelenchus xylophilus* | -99.1458 | | 35.3125 |
| *Bursaphelenchus xylophilus* | -99.2708 | | 31.2708 |
| *Bursaphelenchus xylophilus* | -99.5625 | | 34.6458 |
| *Bursaphelenchus xylophilus* | -99.7708 | | 41.5208 |
| *Bursaphelenchus xylophilus* | 102.2708 | | 27.8958 |
| *Bursaphelenchus xylophilus* | 102.4375 | | 28.3125 |
| *Bursaphelenchus xylophilus* | 102.6875 | | 25.0625 |
| *Bursaphelenchus xylophilus* | 103.1042 | | 30.0625 |
| *Bursaphelenchus xylophilus* | 103.7708 | | 29.5625 |
| *Bursaphelenchus xylophilus* | 104.3542 | | 28.8542 |
| *Bursaphelenchus xylophilus* | 104.5208 | | 28.1875 |
| *Bursaphelenchus xylophilus* | 104.5208 | | 28.4375 |
| *Bursaphelenchus xylophilus* | 104.5208 | | 28.6875 |
| *Bursaphelenchus xylophilus* | 104.5208 | | 32.3958 |
| *Bursaphelenchus xylophilus* | 104.6458 | | 28.7708 |
| *Bursaphelenchus xylophilus* | 104.7292 | | 28.4375 |
| *Bursaphelenchus xylophilus* | 104.7292 | | 29.3542 |
| *Bursaphelenchus xylophilus* | 104.7708 | | 29.3542 |
| *Bursaphelenchus xylophilus* | 104.7708 | | 31.4792 |
| *Bursaphelenchus xylophilus* | 104.8542 | | 29.7708 |
| *Bursaphelenchus xylophilus* | 104.9375 | | 28.6042 |
| *Bursaphelenchus xylophilus* | 104.9792 | | 28.8542 |
| *Bursaphelenchus xylophilus* | 104.9792 | | 29.1875 |
| *Bursaphelenchus xylophilus* | 105.0625 | | 28.7292 |
| *Bursaphelenchus xylophilus* | 105.3125 | | 29.3542 |
| *Bursaphelenchus xylophilus* | 105.3542 | | 30.1042 |
| *Bursaphelenchus xylophilus* | 105.3958 | | 29.1458 |
| *Bursaphelenchus xylophilus* | 105.5208 | | 32.3125 |
| *Bursaphelenchus xylophilus* | 105.6042 | | 29.3958 |
| *Bursaphelenchus xylophilus* | 105.6042 | | 33.3542 |
| *Bursaphelenchus xylophilus* | 105.7292 | | 29.7292 |
| *Bursaphelenchus xylophilus* | 105.8125 | | 28.0625 |
| *Bursaphelenchus xylophilus* | 105.8542 | | 30.1875 |
| *Bursaphelenchus xylophilus* | 105.9375 | | 29.3542 |
| *Bursaphelenchus xylophilus* | 106.0208 | | 31.5625 |
| *Bursaphelenchus xylophilus* | 106.0625 | | 29.8542 |
| *Bursaphelenchus xylophilus* | 106.1042 | | 30.8125 |
| *Bursaphelenchus xylophilus* | 106.1458 | | 30.7708 |
| *Bursaphelenchus xylophilus* | 106.1458 | | 33.3125 |
| *Bursaphelenchus xylophilus* | 106.1875 | | 28.3542 |
| *Bursaphelenchus xylophilus* | 106.2292 | | 24.3125 |
| *Bursaphelenchus xylophilus* | 106.2292 | | 27.4792 |
| *Bursaphelenchus xylophilus* | 106.2292 | | 29.6042 |
| *Bursaphelenchus xylophilus* | 106.2708 | | 29.3125 |
| *Bursaphelenchus xylophilus* | 106.2708 | | 29.9792 |
| *Bursaphelenchus xylophilus* | 106.2708 | | 32.8542 |
| *Bursaphelenchus xylophilus* | 106.3125 | | 31.2708 |
| *Bursaphelenchus xylophilus* | 106.3958 | | 27.8125 |
| *Bursaphelenchus xylophilus* | 106.3958 | | 29.8125 |
| *Bursaphelenchus xylophilus* | 106.4375 | | 23.1458 |
| *Bursaphelenchus xylophilus* | 106.4792 | | 29.4792 |
| *Bursaphelenchus xylophilus* | 106.4792 | | 29.5625 |
| *Bursaphelenchus xylophilus* | 106.5208 | | 29.5208 |
| *Bursaphelenchus xylophilus* | 106.5625 | | 29.3958 |
| *Bursaphelenchus xylophilus* | 106.5625 | | 29.6042 |
| *Bursaphelenchus xylophilus* | 106.6458 | | 29.0208 |
| *Bursaphelenchus xylophilus* | 106.6458 | | 29.7292 |
| *Bursaphelenchus xylophilus* | 106.6458 | | 31.8125 |
| *Bursaphelenchus xylophilus* | 106.6875 | | 29.5208 |
| *Bursaphelenchus xylophilus* | 106.6875 | | 33.1458 |
| *Bursaphelenchus xylophilus* | 106.7708 | | 26.6458 |
| *Bursaphelenchus xylophilus* | 106.7708 | | 30.3958 |
| *Bursaphelenchus xylophilus* | 106.7708 | | 31.8542 |
| *Bursaphelenchus xylophilus* | 106.8542 | | 22.3542 |
| *Bursaphelenchus xylophilus* | 106.8542 | | 27.5625 |
| *Bursaphelenchus xylophilus* | 106.9375 | | 28.8542 |
| *Bursaphelenchus xylophilus* | 106.9375 | | 30.3542 |
| *Bursaphelenchus xylophilus* | 106.9375 | | 33.6042 |
| *Bursaphelenchus xylophilus* | 106.9792 | | 30.8542 |
| *Bursaphelenchus xylophilus* | 107.1042 | | 29.1458 |
| *Bursaphelenchus xylophilus* | 107.1042 | | 29.8542 |
| *Bursaphelenchus xylophilus* | 107.1042 | | 31.5625 |
| *Bursaphelenchus xylophilus* | 107.1875 | | 22.8542 |
| *Bursaphelenchus xylophilus* | 107.2292 | | 30.7292 |
| *Bursaphelenchus xylophilus* | 107.2708 | | 31.9375 |
| *Bursaphelenchus xylophilus* | 107.3542 | | 30.3125 |
| *Bursaphelenchus xylophilus* | 107.3958 | | 29.7292 |
| *Bursaphelenchus xylophilus* | 107.5208 | | 26.6875 |
| *Bursaphelenchus xylophilus* | 107.5208 | | 31.1875 |
| *Bursaphelenchus xylophilus* | 107.5208 | | 31.2292 |
| *Bursaphelenchus xylophilus* | 107.5625 | | 33.2292 |
| *Bursaphelenchus xylophilus* | 107.7292 | | 27.9792 |
| *Bursaphelenchus xylophilus* | 107.7292 | | 29.8542 |
| *Bursaphelenchus xylophilus* | 107.7292 | | 31.3542 |
| *Bursaphelenchus xylophilus* | 107.7708 | | 29.3125 |
| *Bursaphelenchus xylophilus* | 107.7708 | | 30.6458 |
| *Bursaphelenchus xylophilus* | 107.7708 | | 32.9792 |
| *Bursaphelenchus xylophilus* | 107.8958 | | 31.1042 |
| *Bursaphelenchus xylophilus* | 107.8958 | | 32.5625 |
| *Bursaphelenchus xylophilus* | 107.9792 | | 30.2292 |
| *Bursaphelenchus xylophilus* | 107.9792 | | 33.5208 |
| *Bursaphelenchus xylophilus* | 108.0208 | | 32.1042 |
| *Bursaphelenchus xylophilus* | 108.1042 | | 30.0208 |
| *Bursaphelenchus xylophilus* | 108.1875 | | 29.3125 |
| *Bursaphelenchus xylophilus* | 108.2708 | | 22.7708 |
| *Bursaphelenchus xylophilus* | 108.2708 | | 23.1458 |
| *Bursaphelenchus xylophilus* | 108.2708 | | 33.0625 |
| *Bursaphelenchus xylophilus* | 108.3125 | | 22.8542 |
| *Bursaphelenchus xylophilus* | 108.3125 | | 33.3125 |
| *Bursaphelenchus xylophilus* | 108.3542 | | 21.7708 |
| *Bursaphelenchus xylophilus* | 108.3542 | | 22.8125 |
| *Bursaphelenchus xylophilus* | 108.3958 | | 22.8542 |
| *Bursaphelenchus xylophilus* | 108.3958 | | 30.8125 |
| *Bursaphelenchus xylophilus* | 108.3958 | | 31.1458 |
| *Bursaphelenchus xylophilus* | 108.4375 | | 26.7292 |
| *Bursaphelenchus xylophilus* | 108.5208 | | 25.9375 |
| *Bursaphelenchus xylophilus* | 108.5208 | | 32.8958 |
| *Bursaphelenchus xylophilus* | 108.6458 | | 24.4792 |
| *Bursaphelenchus xylophilus* | 108.6875 | | 30.9375 |
| *Bursaphelenchus xylophilus* | 108.6875 | | 31.9375 |
| *Bursaphelenchus xylophilus* | 108.7708 | | 28.8542 |
| *Bursaphelenchus xylophilus* | 108.7708 | | 29.5208 |
| *Bursaphelenchus xylophilus* | 108.8958 | | 25.7708 |
| *Bursaphelenchus xylophilus* | 108.8958 | | 32.3125 |
| *Bursaphelenchus xylophilus* | 108.9375 | | 30.3125 |
| *Bursaphelenchus xylophilus* | 109.0208 | | 28.4375 |
| *Bursaphelenchus xylophilus* | 109.0208 | | 32.6875 |
| *Bursaphelenchus xylophilus* | 109.1042 | | 33.6875 |
| *Bursaphelenchus xylophilus* | 109.1458 | | 27.6875 |
| *Bursaphelenchus xylophilus* | 109.1458 | | 29.6875 |
| *Bursaphelenchus xylophilus* | 109.1458 | | 33.4375 |
| *Bursaphelenchus xylophilus* | 109.1875 | | 27.6875 |
| *Bursaphelenchus xylophilus* | 109.2292 | | 28.1458 |
| *Bursaphelenchus xylophilus* | 109.2708 | | 22.6875 |
| *Bursaphelenchus xylophilus* | 109.3125 | | 22.4375 |
| *Bursaphelenchus xylophilus* | 109.3542 | | 32.3958 |
| *Bursaphelenchus xylophilus* | 109.3542 | | 32.8542 |
| *Bursaphelenchus xylophilus* | 109.3958 | | 24.3542 |
| *Bursaphelenchus xylophilus* | 109.3958 | | 29.4792 |
| *Bursaphelenchus xylophilus* | 109.4375 | | 24.3125 |
| *Bursaphelenchus xylophilus* | 109.4375 | | 29.4792 |
| *Bursaphelenchus xylophilus* | 109.4792 | | 28.5625 |
| *Bursaphelenchus xylophilus* | 109.4792 | | 29.9792 |
| *Bursaphelenchus xylophilus* | 109.4792 | | 30.2708 |
| *Bursaphelenchus xylophilus* | 109.4792 | | 30.3125 |
| *Bursaphelenchus xylophilus* | 109.5625 | | 22.2708 |
| *Bursaphelenchus xylophilus* | 109.5625 | | 23.1042 |
| *Bursaphelenchus xylophilus* | 109.6042 | | 27.9375 |
| *Bursaphelenchus xylophilus* | 109.6458 | | 28.6875 |
| *Bursaphelenchus xylophilus* | 109.6875 | | 26.5625 |
| *Bursaphelenchus xylophilus* | 109.6875 | | 27.4375 |
| *Bursaphelenchus xylophilus* | 109.6875 | | 28.2708 |
| *Bursaphelenchus xylophilus* | 109.7292 | | 32.3125 |
| *Bursaphelenchus xylophilus* | 109.8542 | | 28.9792 |
| *Bursaphelenchus xylophilus* | 109.8958 | | 22.7292 |
| *Bursaphelenchus xylophilus* | 109.8958 | | 31.0625 |
| *Bursaphelenchus xylophilus* | 109.8958 | | 33.5208 |
| *Bursaphelenchus xylophilus* | 109.9375 | | 27.4375 |
| *Bursaphelenchus xylophilus* | 109.9375 | | 28.6042 |
| *Bursaphelenchus xylophilus* | 109.9375 | | 33.8542 |
| *Bursaphelenchus xylophilus* | 109.9792 | | 22.2708 |
| *Bursaphelenchus xylophilus* | 109.9792 | | 24.9792 |
| *Bursaphelenchus xylophilus* | 110.1042 | | 23.3958 |
| *Bursaphelenchus xylophilus* | 110.1042 | | 32.8125 |
| *Bursaphelenchus xylophilus* | 110.1458 | | 22.6458 |
| *Bursaphelenchus xylophilus* | 110.1458 | | 26.6042 |
| *Bursaphelenchus xylophilus* | 110.1458 | | 34.1042 |
| *Bursaphelenchus xylophilus* | 110.1875 | | 24.1458 |
| *Bursaphelenchus xylophilus* | 110.2292 | | 25.2292 |
| *Bursaphelenchus xylophilus* | 110.3125 | | 25.3958 |
| *Bursaphelenchus xylophilus* | 110.3125 | | 26.3958 |
| *Bursaphelenchus xylophilus* | 110.3542 | | 31.0625 |
| *Bursaphelenchus xylophilus* | 110.3542 | | 33.6875 |
| *Bursaphelenchus xylophilus* | 110.3958 | | 28.4792 |
| *Bursaphelenchus xylophilus* | 110.4375 | | 32.9792 |
| *Bursaphelenchus xylophilus* | 110.5625 | | 22.8542 |
| *Bursaphelenchus xylophilus* | 110.5625 | | 29.1458 |
| *Bursaphelenchus xylophilus* | 110.5625 | | 29.3542 |
| *Bursaphelenchus xylophilus* | 110.6458 | | 26.7292 |
| *Bursaphelenchus xylophilus* | 110.6875 | | 25.6042 |
| *Bursaphelenchus xylophilus* | 110.7292 | | 32.0625 |
| *Bursaphelenchus xylophilus* | 110.7708 | | 31.3542 |
| *Bursaphelenchus xylophilus* | 110.7708 | | 32.6458 |
| *Bursaphelenchus xylophilus* | 110.8125 | | 32.6042 |
| *Bursaphelenchus xylophilus* | 110.8125 | | 32.8542 |
| *Bursaphelenchus xylophilus* | 110.8542 | | 21.9375 |
| *Bursaphelenchus xylophilus* | 110.8542 | | 24.8542 |
| *Bursaphelenchus xylophilus* | 110.8958 | | 32.5625 |
| *Bursaphelenchus xylophilus* | 110.8958 | | 33.5208 |
| *Bursaphelenchus xylophilus* | 110.9792 | | 30.8125 |
| *Bursaphelenchus xylophilus* | 111.0208 | | 22.9375 |
| *Bursaphelenchus xylophilus* | 111.0625 | | 25.9375 |
| *Bursaphelenchus xylophilus* | 111.0625 | | 30.1875 |
| *Bursaphelenchus xylophilus* | 111.0625 | | 34.0625 |
| *Bursaphelenchus xylophilus* | 111.1042 | | 32.5208 |
| *Bursaphelenchus xylophilus* | 111.1458 | | 29.4375 |
| *Bursaphelenchus xylophilus* | 111.2292 | | 28.3958 |
| *Bursaphelenchus xylophilus* | 111.2292 | | 30.4792 |
| *Bursaphelenchus xylophilus* | 111.2708 | | 23.4792 |
| *Bursaphelenchus xylophilus* | 111.2708 | | 26.9792 |
| *Bursaphelenchus xylophilus* | 111.2708 | | 30.6875 |
| *Bursaphelenchus xylophilus* | 111.2708 | | 31.8958 |
| *Bursaphelenchus xylophilus* | 111.3125 | | 23.4792 |
| *Bursaphelenchus xylophilus* | 111.3125 | | 24.5208 |
| *Bursaphelenchus xylophilus* | 111.3125 | | 26.3958 |
| *Bursaphelenchus xylophilus* | 111.3125 | | 30.7708 |
| *Bursaphelenchus xylophilus* | 111.3542 | | 25.2708 |
| *Bursaphelenchus xylophilus* | 111.3542 | | 27.7292 |
| *Bursaphelenchus xylophilus* | 111.3542 | | 30.7292 |
| *Bursaphelenchus xylophilus* | 111.4375 | | 27.2292 |
| *Bursaphelenchus xylophilus* | 111.4375 | | 30.3958 |
| *Bursaphelenchus xylophilus* | 111.4375 | | 30.5208 |
| *Bursaphelenchus xylophilus* | 111.4792 | | 24.4792 |
| *Bursaphelenchus xylophilus* | 111.4792 | | 27.3125 |
| *Bursaphelenchus xylophilus* | 111.4792 | | 28.8958 |
| *Bursaphelenchus xylophilus* | 111.4792 | | 33.1458 |
| *Bursaphelenchus xylophilus* | 111.4792 | | 33.3125 |
| *Bursaphelenchus xylophilus* | 111.5208 | | 23.2292 |
| *Bursaphelenchus xylophilus* | 111.5208 | | 23.4375 |
| *Bursaphelenchus xylophilus* | 111.5208 | | 27.2292 |
| *Bursaphelenchus xylophilus* | 111.5208 | | 32.5625 |
| *Bursaphelenchus xylophilus* | 111.5625 | | 22.7708 |
| *Bursaphelenchus xylophilus* | 111.5625 | | 23.8542 |
| *Bursaphelenchus xylophilus* | 111.5625 | | 24.4375 |
| *Bursaphelenchus xylophilus* | 111.6042 | | 26.4792 |
| *Bursaphelenchus xylophilus* | 111.6042 | | 33.8125 |
| *Bursaphelenchus xylophilus* | 111.6458 | | 26.2292 |
| *Bursaphelenchus xylophilus* | 111.6458 | | 29.4375 |
| *Bursaphelenchus xylophilus* | 111.6458 | | 31.0625 |
| *Bursaphelenchus xylophilus* | 111.6458 | | 32.2708 |
| *Bursaphelenchus xylophilus* | 111.6875 | | 27.6875 |
| *Bursaphelenchus xylophilus* | 111.6875 | | 29.0208 |
| *Bursaphelenchus xylophilus* | 111.7708 | | 23.1458 |
| *Bursaphelenchus xylophilus* | 111.7708 | | 27.2708 |
| *Bursaphelenchus xylophilus* | 111.7708 | | 30.1875 |
| *Bursaphelenchus xylophilus* | 111.7708 | | 30.4375 |
| *Bursaphelenchus xylophilus* | 111.8125 | | 22.1875 |
| *Bursaphelenchus xylophilus* | 111.8125 | | 30.8125 |
| *Bursaphelenchus xylophilus* | 111.8542 | | 31.7708 |
| *Bursaphelenchus xylophilus* | 111.9375 | | 25.5625 |
| *Bursaphelenchus xylophilus* | 111.9792 | | 21.8542 |
| *Bursaphelenchus xylophilus* | 112.0208 | | 27.7292 |
| *Bursaphelenchus xylophilus* | 112.1042 | | 24.5625 |
| *Bursaphelenchus xylophilus* | 112.1042 | | 26.8125 |
| *Bursaphelenchus xylophilus* | 112.1458 | | 28.5208 |
| *Bursaphelenchus xylophilus* | 112.1458 | | 32.0625 |
| *Bursaphelenchus xylophilus* | 112.1875 | | 23.9375 |
| *Bursaphelenchus xylophilus* | 112.1875 | | 27.4792 |
| *Bursaphelenchus xylophilus* | 112.1875 | | 29.4375 |
| *Bursaphelenchus xylophilus* | 112.1875 | | 30.3542 |
| *Bursaphelenchus xylophilus* | 112.2292 | | 30.9792 |
| *Bursaphelenchus xylophilus* | 112.2292 | | 31.0625 |
| *Bursaphelenchus xylophilus* | 112.2292 | | 32.1042 |
| *Bursaphelenchus xylophilus* | 112.2708 | | 31.7292 |
| *Bursaphelenchus xylophilus* | 112.3125 | | 24.7292 |
| *Bursaphelenchus xylophilus* | 112.3542 | | 29.6875 |
| *Bursaphelenchus xylophilus* | 112.3958 | | 24.7708 |
| *Bursaphelenchus xylophilus* | 112.3958 | | 26.4375 |
| *Bursaphelenchus xylophilus* | 112.3958 | | 26.9792 |
| *Bursaphelenchus xylophilus* | 112.4375 | | 23.6458 |
| *Bursaphelenchus xylophilus* | 112.5208 | | 27.7292 |
| *Bursaphelenchus xylophilus* | 112.5625 | | 23.1458 |
| *Bursaphelenchus xylophilus* | 112.5625 | | 26.9375 |
| *Bursaphelenchus xylophilus* | 112.5625 | | 28.2708 |
| *Bursaphelenchus xylophilus* | 112.6042 | | 26.8542 |
| *Bursaphelenchus xylophilus* | 112.6042 | | 31.1875 |
| *Bursaphelenchus xylophilus* | 112.6458 | | 24.4792 |
| *Bursaphelenchus xylophilus* | 112.6458 | | 26.8958 |
| *Bursaphelenchus xylophilus* | 112.6875 | | 26.7292 |
| *Bursaphelenchus xylophilus* | 112.7292 | | 23.3125 |
| *Bursaphelenchus xylophilus* | 112.7292 | | 25.7708 |
| *Bursaphelenchus xylophilus* | 112.7292 | | 27.2292 |
| *Bursaphelenchus xylophilus* | 112.7708 | | 32.1458 |
| *Bursaphelenchus xylophilus* | 112.8125 | | 28.3542 |
| *Bursaphelenchus xylophilus* | 112.8958 | | 22.8958 |
| *Bursaphelenchus xylophilus* | 112.8958 | | 23.1458 |
| *Bursaphelenchus xylophilus* | 112.8958 | | 27.2292 |
| *Bursaphelenchus xylophilus* | 112.9375 | | 25.3958 |
| *Bursaphelenchus xylophilus* | 112.9375 | | 27.7708 |
| *Bursaphelenchus xylophilus* | 112.9375 | | 28.2292 |
| *Bursaphelenchus xylophilus* | 112.9792 | | 22.7708 |
| *Bursaphelenchus xylophilus* | 112.9792 | | 23.7708 |
| *Bursaphelenchus xylophilus* | 112.9792 | | 27.1042 |
| *Bursaphelenchus xylophilus* | 112.9792 | | 28.2708 |
| *Bursaphelenchus xylophilus* | 113.0208 | | 22.4792 |
| *Bursaphelenchus xylophilus* | 113.0208 | | 27.9375 |
| *Bursaphelenchus xylophilus* | 113.0625 | | 23.6875 |
| *Bursaphelenchus xylophilus* | 113.0625 | | 28.1458 |
| *Bursaphelenchus xylophilus* | 113.0625 | | 28.8125 |
| *Bursaphelenchus xylophilus* | 113.1042 | | 22.6042 |
| *Bursaphelenchus xylophilus* | 113.1042 | | 26.1458 |
| *Bursaphelenchus xylophilus* | 113.1042 | | 27.8958 |
| *Bursaphelenchus xylophilus* | 113.1042 | | 28.2708 |
| *Bursaphelenchus xylophilus* | 113.1042 | | 29.1458 |
| *Bursaphelenchus xylophilus* | 113.1458 | | 23.0208 |
| *Bursaphelenchus xylophilus* | 113.1458 | | 27.7708 |
| *Bursaphelenchus xylophilus* | 113.1458 | | 31.0208 |
| *Bursaphelenchus xylophilus* | 113.2292 | | 23.3958 |
| *Bursaphelenchus xylophilus* | 113.2292 | | 25.9792 |
| *Bursaphelenchus xylophilus* | 113.2708 | | 23.1458 |
| *Bursaphelenchus xylophilus* | 113.2708 | | 24.7708 |
| *Bursaphelenchus xylophilus* | 113.2708 | | 26.7292 |
| *Bursaphelenchus xylophilus* | 113.2708 | | 29.4792 |
| *Bursaphelenchus xylophilus* | 113.3125 | | 31.8542 |
| *Bursaphelenchus xylophilus* | 113.3542 | | 25.1458 |
| *Bursaphelenchus xylophilus* | 113.3542 | | 27.0208 |
| *Bursaphelenchus xylophilus* | 113.3958 | | 22.5208 |
| *Bursaphelenchus xylophilus* | 113.3958 | | 31.7292 |
| *Bursaphelenchus xylophilus* | 113.4375 | | 24.1875 |
| *Bursaphelenchus xylophilus* | 113.4375 | | 29.4792 |
| *Bursaphelenchus xylophilus* | 113.4792 | | 23.1875 |
| *Bursaphelenchus xylophilus* | 113.5208 | | 23.8958 |
| *Bursaphelenchus xylophilus* | 113.5208 | | 27.6458 |
| *Bursaphelenchus xylophilus* | 113.5625 | | 22.2708 |
| *Bursaphelenchus xylophilus* | 113.5625 | | 26.7708 |
| *Bursaphelenchus xylophilus* | 113.6042 | | 23.5625 |
| *Bursaphelenchus xylophilus* | 113.6042 | | 24.6875 |
| *Bursaphelenchus xylophilus* | 113.6042 | | 24.8125 |
| *Bursaphelenchus xylophilus* | 113.6042 | | 28.6875 |
| *Bursaphelenchus xylophilus* | 113.6458 | | 28.1875 |
| *Bursaphelenchus xylophilus* | 113.6875 | | 31.2708 |
| *Bursaphelenchus xylophilus* | 113.7708 | | 23.0208 |
| *Bursaphelenchus xylophilus* | 113.7708 | | 25.1042 |
| *Bursaphelenchus xylophilus* | 113.7708 | | 26.4792 |
| *Bursaphelenchus xylophilus* | 113.8125 | | 23.2708 |
| *Bursaphelenchus xylophilus* | 113.8125 | | 29.2708 |
| *Bursaphelenchus xylophilus* | 113.8125 | | 31.6042 |
| *Bursaphelenchus xylophilus* | 113.8958 | | 29.7292 |
| *Bursaphelenchus xylophilus* | 113.9375 | | 29.9792 |
| *Bursaphelenchus xylophilus* | 114.0208 | | 30.5625 |
| *Bursaphelenchus xylophilus* | 114.0208 | | 31.2708 |
| *Bursaphelenchus xylophilus* | 114.0208 | | 32.8125 |
| *Bursaphelenchus xylophilus* | 114.0625 | | 24.9792 |
| *Bursaphelenchus xylophilus* | 114.0625 | | 29.5625 |
| *Bursaphelenchus xylophilus* | 114.1042 | | 30.3542 |
| *Bursaphelenchus xylophilus* | 114.1458 | | 24.3542 |
| *Bursaphelenchus xylophilus* | 114.1458 | | 31.5625 |
| *Bursaphelenchus xylophilus* | 114.1875 | | 22.2292 |
| *Bursaphelenchus xylophilus* | 114.2292 | | 24.0625 |
| *Bursaphelenchus xylophilus* | 114.2292 | | 26.9375 |
| *Bursaphelenchus xylophilus* | 114.2708 | | 23.7292 |
| *Bursaphelenchus xylophilus* | 114.3125 | | 23.1875 |
| *Bursaphelenchus xylophilus* | 114.3125 | | 25.1042 |
| *Bursaphelenchus xylophilus* | 114.3125 | | 25.6875 |
| *Bursaphelenchus xylophilus* | 114.3125 | | 26.7708 |
| *Bursaphelenchus xylophilus* | 114.3125 | | 29.8542 |
| *Bursaphelenchus xylophilus* | 114.3125 | | 30.3958 |
| *Bursaphelenchus xylophilus* | 114.3125 | | 30.5625 |
| *Bursaphelenchus xylophilus* | 114.3542 | | 25.3958 |
| *Bursaphelenchus xylophilus* | 114.3542 | | 28.5208 |
| *Bursaphelenchus xylophilus* | 114.3542 | | 30.5208 |
| *Bursaphelenchus xylophilus* | 114.3958 | | 23.1042 |
| *Bursaphelenchus xylophilus* | 114.3958 | | 30.4792 |
| *Bursaphelenchus xylophilus* | 114.3958 | | 30.6458 |
| *Bursaphelenchus xylophilus* | 114.3958 | | 30.8958 |
| *Bursaphelenchus xylophilus* | 114.4375 | | 27.8125 |
| *Bursaphelenchus xylophilus* | 114.4375 | | 28.1042 |
| *Bursaphelenchus xylophilus* | 114.4375 | | 30.5625 |
| *Bursaphelenchus xylophilus* | 114.4792 | | 22.8125 |
| *Bursaphelenchus xylophilus* | 114.4792 | | 24.3958 |
| *Bursaphelenchus xylophilus* | 114.4792 | | 29.6042 |
| *Bursaphelenchus xylophilus* | 114.5208 | | 24.7292 |
| *Bursaphelenchus xylophilus* | 114.5208 | | 26.3125 |
| *Bursaphelenchus xylophilus* | 114.5208 | | 32.2292 |
| *Bursaphelenchus xylophilus* | 114.5625 | | 25.7708 |
| *Bursaphelenchus xylophilus* | 114.5625 | | 29.0208 |
| *Bursaphelenchus xylophilus* | 114.6042 | | 31.3125 |
| *Bursaphelenchus xylophilus* | 114.6458 | | 27.3958 |
| *Bursaphelenchus xylophilus* | 114.6875 | | 30.1042 |
| *Bursaphelenchus xylophilus* | 114.7292 | | 22.9792 |
| *Bursaphelenchus xylophilus* | 114.7292 | | 23.7292 |
| *Bursaphelenchus xylophilus* | 114.7708 | | 23.8125 |
| *Bursaphelenchus xylophilus* | 114.7708 | | 25.6875 |
| *Bursaphelenchus xylophilus* | 114.8125 | | 24.8958 |
| *Bursaphelenchus xylophilus* | 114.8125 | | 26.4792 |
| *Bursaphelenchus xylophilus* | 114.8125 | | 28.3958 |
| *Bursaphelenchus xylophilus* | 114.8125 | | 30.8542 |
| *Bursaphelenchus xylophilus* | 114.8958 | | 26.8125 |
| *Bursaphelenchus xylophilus* | 114.8958 | | 27.0625 |
| *Bursaphelenchus xylophilus* | 114.8958 | | 30.2292 |
| *Bursaphelenchus xylophilus* | 114.8958 | | 30.3958 |
| *Bursaphelenchus xylophilus* | 114.8958 | | 30.6458 |
| *Bursaphelenchus xylophilus* | 114.8958 | | 31.6458 |
| *Bursaphelenchus xylophilus* | 114.9375 | | 24.4375 |
| *Bursaphelenchus xylophilus* | 114.9375 | | 25.3958 |
| *Bursaphelenchus xylophilus* | 114.9375 | | 25.8125 |
| *Bursaphelenchus xylophilus* | 114.9375 | | 27.8125 |
| *Bursaphelenchus xylophilus* | 114.9375 | | 28.2292 |
| *Bursaphelenchus xylophilus* | 114.9375 | | 32.0208 |
| *Bursaphelenchus xylophilus* | 114.9792 | | 30.1042 |
| *Bursaphelenchus xylophilus* | 114.9792 | | 30.1875 |
| *Bursaphelenchus xylophilus* | 115.0208 | | 24.7708 |
| *Bursaphelenchus xylophilus* | 115.0208 | | 25.8542 |
| *Bursaphelenchus xylophilus* | 115.0208 | | 27.1042 |
| *Bursaphelenchus xylophilus* | 115.0208 | | 31.1875 |
| *Bursaphelenchus xylophilus* | 115.0625 | | 30.2292 |
| *Bursaphelenchus xylophilus* | 115.1042 | | 29.2708 |
| *Bursaphelenchus xylophilus* | 115.1458 | | 27.2292 |
| *Bursaphelenchus xylophilus* | 115.1875 | | 23.6458 |
| *Bursaphelenchus xylophilus* | 115.2292 | | 29.8542 |
| *Bursaphelenchus xylophilus* | 115.2708 | | 24.1042 |
| *Bursaphelenchus xylophilus* | 115.2708 | | 30.4375 |
| *Bursaphelenchus xylophilus* | 115.3125 | | 22.9792 |
| *Bursaphelenchus xylophilus* | 115.3125 | | 27.6042 |
| *Bursaphelenchus xylophilus* | 115.3542 | | 26.3542 |
| *Bursaphelenchus xylophilus* | 115.3542 | | 28.4375 |
| *Bursaphelenchus xylophilus* | 115.3542 | | 28.8542 |
| *Bursaphelenchus xylophilus* | 115.3958 | | 25.1458 |
| *Bursaphelenchus xylophilus* | 115.3958 | | 27.7292 |
| *Bursaphelenchus xylophilus* | 115.3958 | | 28.6875 |
| *Bursaphelenchus xylophilus* | 115.3958 | | 30.7708 |
| *Bursaphelenchus xylophilus* | 115.4375 | | 25.9375 |
| *Bursaphelenchus xylophilus* | 115.4375 | | 27.3125 |
| *Bursaphelenchus xylophilus* | 115.4375 | | 30.2292 |
| *Bursaphelenchus xylophilus* | 115.5625 | | 28.0625 |
| *Bursaphelenchus xylophilus* | 115.5625 | | 28.8542 |
| *Bursaphelenchus xylophilus* | 115.5625 | | 29.8542 |
| *Bursaphelenchus xylophilus* | 115.6458 | | 23.3125 |
| *Bursaphelenchus xylophilus* | 115.6458 | | 24.9792 |
| *Bursaphelenchus xylophilus* | 115.6875 | | 29.6875 |
| *Bursaphelenchus xylophilus* | 115.6875 | | 30.7292 |
| *Bursaphelenchus xylophilus* | 115.7292 | | 24.1458 |
| *Bursaphelenchus xylophilus* | 115.7292 | | 28.7292 |
| *Bursaphelenchus xylophilus* | 115.7708 | | 23.9375 |
| *Bursaphelenchus xylophilus* | 115.7708 | | 28.1458 |
| *Bursaphelenchus xylophilus* | 115.7708 | | 29.3125 |
| *Bursaphelenchus xylophilus* | 115.8125 | | 25.6042 |
| *Bursaphelenchus xylophilus* | 115.8125 | | 28.6875 |
| *Bursaphelenchus xylophilus* | 115.8125 | | 29.2292 |
| *Bursaphelenchus xylophilus* | 115.8542 | | 23.4375 |
| *Bursaphelenchus xylophilus* | 115.8542 | | 27.4375 |
| *Bursaphelenchus xylophilus* | 115.8542 | | 29.0208 |
| *Bursaphelenchus xylophilus* | 115.8958 | | 24.5625 |
| *Bursaphelenchus xylophilus* | 115.9375 | | 29.6042 |
| *Bursaphelenchus xylophilus* | 115.9375 | | 30.0625 |
| *Bursaphelenchus xylophilus* | 115.9375 | | 31.7292 |
| *Bursaphelenchus xylophilus* | 115.9375 | | 31.8542 |
| *Bursaphelenchus xylophilus* | 115.9792 | | 29.6875 |
| *Bursaphelenchus xylophilus* | 116.0208 | | 25.8958 |
| *Bursaphelenchus xylophilus* | 116.0208 | | 26.4792 |
| *Bursaphelenchus xylophilus* | 116.0625 | | 29.4375 |
| *Bursaphelenchus xylophilus* | 116.1042 | | 24.2708 |
| *Bursaphelenchus xylophilus* | 116.1042 | | 24.3125 |
| *Bursaphelenchus xylophilus* | 116.1042 | | 27.7708 |
| *Bursaphelenchus xylophilus* | 116.1458 | | 30.1458 |
| *Bursaphelenchus xylophilus* | 116.1875 | | 23.3125 |
| *Bursaphelenchus xylophilus* | 116.1875 | | 23.7292 |
| *Bursaphelenchus xylophilus* | 116.1875 | | 24.6458 |
| *Bursaphelenchus xylophilus* | 116.2292 | | 27.5625 |
| *Bursaphelenchus xylophilus* | 116.2292 | | 28.3958 |
| *Bursaphelenchus xylophilus* | 116.2292 | | 29.2708 |
| *Bursaphelenchus xylophilus* | 116.2708 | | 32.3542 |
| *Bursaphelenchus xylophilus* | 116.3125 | | 23.0208 |
| *Bursaphelenchus xylophilus* | 116.3125 | | 27.9375 |
| *Bursaphelenchus xylophilus* | 116.3125 | | 30.4792 |
| *Bursaphelenchus xylophilus* | 116.3542 | | 23.5208 |
| *Bursaphelenchus xylophilus* | 116.3542 | | 26.8542 |
| *Bursaphelenchus xylophilus* | 116.3542 | | 30.8542 |
| *Bursaphelenchus xylophilus* | 116.3542 | | 31.3958 |
| *Bursaphelenchus xylophilus* | 116.4375 | | 23.2708 |
| *Bursaphelenchus xylophilus* | 116.4375 | | 23.5208 |
| *Bursaphelenchus xylophilus* | 116.4375 | | 23.5625 |
| *Bursaphelenchus xylophilus* | 116.4375 | | 25.0625 |
| *Bursaphelenchus xylophilus* | 116.4792 | | 31.7292 |
| *Bursaphelenchus xylophilus* | 116.5208 | | 27.2292 |
| *Bursaphelenchus xylophilus* | 116.5625 | | 31.7708 |
| *Bursaphelenchus xylophilus* | 116.6042 | | 28.2708 |
| *Bursaphelenchus xylophilus* | 116.6042 | | 30.6458 |
| *Bursaphelenchus xylophilus* | 116.6458 | | 23.6875 |
| *Bursaphelenchus xylophilus* | 116.6458 | | 27.5625 |
| *Bursaphelenchus xylophilus* | 116.6875 | | 23.4792 |
| *Bursaphelenchus xylophilus* | 116.6875 | | 24.3542 |
| *Bursaphelenchus xylophilus* | 116.6875 | | 28.6875 |
| *Bursaphelenchus xylophilus* | 116.6875 | | 30.1458 |
| *Bursaphelenchus xylophilus* | 116.7292 | | 23.2708 |
| *Bursaphelenchus xylophilus* | 116.7292 | | 29.0208 |
| *Bursaphelenchus xylophilus* | 116.7708 | | 23.4792 |
| *Bursaphelenchus xylophilus* | 116.7708 | | 25.7292 |
| *Bursaphelenchus xylophilus* | 116.7708 | | 27.9375 |
| *Bursaphelenchus xylophilus* | 116.8125 | | 26.1875 |
| *Bursaphelenchus xylophilus* | 116.8125 | | 28.2292 |
| *Bursaphelenchus xylophilus* | 116.8542 | | 26.8542 |
| *Bursaphelenchus xylophilus* | 116.8542 | | 30.7292 |
| *Bursaphelenchus xylophilus* | 116.8958 | | 27.2708 |
| *Bursaphelenchus xylophilus* | 116.9375 | | 31.0208 |
| *Bursaphelenchus xylophilus* | 116.9375 | | 31.4792 |
| *Bursaphelenchus xylophilus* | 116.9792 | | 30.6042 |
| *Bursaphelenchus xylophilus* | 117.0208 | | 23.4375 |
| *Bursaphelenchus xylophilus* | 117.0208 | | 23.6875 |
| *Bursaphelenchus xylophilus* | 117.0208 | | 30.1042 |
| *Bursaphelenchus xylophilus* | 117.0208 | | 30.5208 |
| *Bursaphelenchus xylophilus* | 117.0625 | | 27.7292 |
| *Bursaphelenchus xylophilus* | 117.0625 | | 28.2292 |
| *Bursaphelenchus xylophilus* | 117.0625 | | 28.6875 |
| *Bursaphelenchus xylophilus* | 117.0625 | | 36.1875 |
| *Bursaphelenchus xylophilus* | 117.1458 | | 28.9792 |
| *Bursaphelenchus xylophilus* | 117.1458 | | 31.7292 |
| *Bursaphelenchus xylophilus* | 117.1458 | | 36.1875 |
| *Bursaphelenchus xylophilus* | 117.1875 | | 23.7292 |
| *Bursaphelenchus xylophilus* | 117.1875 | | 26.8958 |
| *Bursaphelenchus xylophilus* | 117.1875 | | 29.2708 |
| *Bursaphelenchus xylophilus* | 117.2292 | | 29.3542 |
| *Bursaphelenchus xylophilus* | 117.2708 | | 28.3125 |
| *Bursaphelenchus xylophilus* | 117.2708 | | 30.7292 |
| *Bursaphelenchus xylophilus* | 117.2708 | | 35.6875 |
| *Bursaphelenchus xylophilus* | 117.3125 | | 31.2708 |
| *Bursaphelenchus xylophilus* | 117.3542 | | 24.5208 |
| *Bursaphelenchus xylophilus* | 117.3542 | | 25.9375 |
| *Bursaphelenchus xylophilus* | 117.3542 | | 27.5625 |
| *Bursaphelenchus xylophilus* | 117.4375 | | 25.3125 |
| *Bursaphelenchus xylophilus* | 117.4375 | | 28.3958 |
| *Bursaphelenchus xylophilus* | 117.4792 | | 26.7292 |
| *Bursaphelenchus xylophilus* | 117.4792 | | 27.3542 |
| *Bursaphelenchus xylophilus* | 117.4792 | | 30.2292 |
| *Bursaphelenchus xylophilus* | 117.4792 | | 31.8958 |
| *Bursaphelenchus xylophilus* | 117.5208 | | 32.8958 |
| *Bursaphelenchus xylophilus* | 117.5625 | | 30.6875 |
| *Bursaphelenchus xylophilus* | 117.6042 | | 28.3958 |
| *Bursaphelenchus xylophilus* | 117.6458 | | 26.2708 |
| *Bursaphelenchus xylophilus* | 117.6458 | | 36.2292 |
| *Bursaphelenchus xylophilus* | 117.6875 | | 32.5208 |
| *Bursaphelenchus xylophilus* | 117.7292 | | 28.3125 |
| *Bursaphelenchus xylophilus* | 117.7292 | | 29.8542 |
| *Bursaphelenchus xylophilus* | 117.7708 | | 30.8125 |
| *Bursaphelenchus xylophilus* | 117.8125 | | 26.3958 |
| *Bursaphelenchus xylophilus* | 117.8125 | | 26.8125 |
| *Bursaphelenchus xylophilus* | 117.8542 | | 25.6875 |
| *Bursaphelenchus xylophilus* | 117.8542 | | 29.2708 |
| *Bursaphelenchus xylophilus* | 117.8542 | | 30.6458 |
| *Bursaphelenchus xylophilus* | 117.8958 | | 28.4375 |
| *Bursaphelenchus xylophilus* | 117.8958 | | 31.3125 |
| *Bursaphelenchus xylophilus* | 117.8958 | | 31.6458 |
| *Bursaphelenchus xylophilus* | 117.9375 | | 29.9375 |
| *Bursaphelenchus xylophilus* | 117.9792 | | 28.4375 |
| *Bursaphelenchus xylophilus* | 117.9792 | | 32.7708 |
| *Bursaphelenchus xylophilus* | 118.0208 | | 27.7708 |
| *Bursaphelenchus xylophilus* | 118.1458 | | 24.7292 |
| *Bursaphelenchus xylophilus* | 118.1458 | | 27.3542 |
| *Bursaphelenchus xylophilus* | 118.1458 | | 30.2708 |
| *Bursaphelenchus xylophilus* | 118.1875 | | 25.0625 |
| *Bursaphelenchus xylophilus* | 118.1875 | | 26.6458 |
| *Bursaphelenchus xylophilus* | 118.1875 | | 28.4375 |
| *Bursaphelenchus xylophilus* | 118.1875 | | 29.7708 |
| *Bursaphelenchus xylophilus* | 118.2708 | | 24.6042 |
| *Bursaphelenchus xylophilus* | 118.2708 | | 28.6875 |
| *Bursaphelenchus xylophilus* | 118.2708 | | 32.1042 |
| *Bursaphelenchus xylophilus* | 118.3125 | | 25.3125 |
| *Bursaphelenchus xylophilus* | 118.3125 | | 27.0208 |
| *Bursaphelenchus xylophilus* | 118.3125 | | 29.6875 |
| *Bursaphelenchus xylophilus* | 118.3542 | | 29.8542 |
| *Bursaphelenchus xylophilus* | 118.3542 | | 30.9375 |
| *Bursaphelenchus xylophilus* | 118.3958 | | 24.9792 |
| *Bursaphelenchus xylophilus* | 118.4375 | | 29.1458 |
| *Bursaphelenchus xylophilus* | 118.4375 | | 29.8542 |
| *Bursaphelenchus xylophilus* | 118.4375 | | 30.6875 |
| *Bursaphelenchus xylophilus* | 118.4375 | | 32.1875 |
| *Bursaphelenchus xylophilus* | 118.4375 | | 32.4792 |
| *Bursaphelenchus xylophilus* | 118.5208 | | 28.8958 |
| *Bursaphelenchus xylophilus* | 118.5208 | | 31.5625 |
| *Bursaphelenchus xylophilus* | 118.5625 | | 24.7708 |
| *Bursaphelenchus xylophilus* | 118.5625 | | 27.9375 |
| *Bursaphelenchus xylophilus* | 118.5625 | | 30.3125 |
| *Bursaphelenchus xylophilus* | 118.5625 | | 33.0208 |
| *Bursaphelenchus xylophilus* | 118.6042 | | 24.8958 |
| *Bursaphelenchus xylophilus* | 118.6042 | | 30.0625 |
| *Bursaphelenchus xylophilus* | 118.6458 | | 32.0625 |
| *Bursaphelenchus xylophilus* | 118.6458 | | 34.9375 |
| *Bursaphelenchus xylophilus* | 118.6875 | | 24.9375 |
| *Bursaphelenchus xylophilus* | 118.6875 | | 25.3542 |
| *Bursaphelenchus xylophilus* | 118.7708 | | 26.5625 |
| *Bursaphelenchus xylophilus* | 118.7708 | | 27.5208 |
| *Bursaphelenchus xylophilus* | 118.7708 | | 30.9375 |
| *Bursaphelenchus xylophilus* | 118.7708 | | 31.9792 |
| *Bursaphelenchus xylophilus* | 118.8125 | | 25.0208 |
| *Bursaphelenchus xylophilus* | 118.8125 | | 31.5625 |
| *Bursaphelenchus xylophilus* | 118.8125 | | 32.0625 |
| *Bursaphelenchus xylophilus* | 118.8125 | | 32.3125 |
| *Bursaphelenchus xylophilus* | 118.8542 | | 26.2292 |
| *Bursaphelenchus xylophilus* | 118.8542 | | 27.3542 |
| *Bursaphelenchus xylophilus* | 118.8542 | | 31.9792 |
| *Bursaphelenchus xylophilus* | 118.8542 | | 35.1875 |
| *Bursaphelenchus xylophilus* | 118.8958 | | 28.9792 |
| *Bursaphelenchus xylophilus* | 118.8958 | | 31.3542 |
| *Bursaphelenchus xylophilus* | 118.8958 | | 32.1042 |
| *Bursaphelenchus xylophilus* | 118.9375 | | 25.8542 |
| *Bursaphelenchus xylophilus* | 118.9792 | | 28.9792 |
| *Bursaphelenchus xylophilus* | 118.9792 | | 30.6458 |
| *Bursaphelenchus xylophilus* | 119.0208 | | 25.4375 |
| *Bursaphelenchus xylophilus* | 119.0208 | | 31.6458 |
| *Bursaphelenchus xylophilus* | 119.0625 | | 27.6458 |
| *Bursaphelenchus xylophilus* | 119.0625 | | 29.6042 |
| *Bursaphelenchus xylophilus* | 119.1042 | | 25.4792 |
| *Bursaphelenchus xylophilus* | 119.1458 | | 26.1458 |
| *Bursaphelenchus xylophilus* | 119.1458 | | 28.0625 |
| *Bursaphelenchus xylophilus* | 119.1875 | | 29.0208 |
| *Bursaphelenchus xylophilus* | 119.1875 | | 31.9375 |
| *Bursaphelenchus xylophilus* | 119.1875 | | 34.5625 |
| *Bursaphelenchus xylophilus* | 119.1875 | | 34.8542 |
| *Bursaphelenchus xylophilus* | 119.2292 | | 34.2708 |
| *Bursaphelenchus xylophilus* | 119.2292 | | 35.7708 |
| *Bursaphelenchus xylophilus* | 119.2708 | | 28.6042 |
| *Bursaphelenchus xylophilus* | 119.2708 | | 29.4792 |
| *Bursaphelenchus xylophilus* | 119.3125 | | 35.1458 |
| *Bursaphelenchus xylophilus* | 119.3542 | | 26.1042 |
| *Bursaphelenchus xylophilus* | 119.3542 | | 27.1042 |
| *Bursaphelenchus xylophilus* | 119.3542 | | 34.7708 |
| *Bursaphelenchus xylophilus* | 119.3958 | | 25.7292 |
| *Bursaphelenchus xylophilus* | 119.3958 | | 32.1875 |
| *Bursaphelenchus xylophilus* | 119.4375 | | 26.0208 |
| *Bursaphelenchus xylophilus* | 119.4375 | | 30.8958 |
| *Bursaphelenchus xylophilus* | 119.4375 | | 32.1458 |
| *Bursaphelenchus xylophilus* | 119.4375 | | 32.1875 |
| *Bursaphelenchus xylophilus* | 119.4792 | | 28.4375 |
| *Bursaphelenchus xylophilus* | 119.4792 | | 29.2292 |
| *Bursaphelenchus xylophilus* | 119.4792 | | 31.4375 |
| *Bursaphelenchus xylophilus* | 119.4792 | | 35.4375 |
| *Bursaphelenchus xylophilus* | 119.5208 | | 25.9792 |
| *Bursaphelenchus xylophilus* | 119.5208 | | 26.6875 |
| *Bursaphelenchus xylophilus* | 119.5208 | | 27.4792 |
| *Bursaphelenchus xylophilus* | 119.5625 | | 26.1875 |
| *Bursaphelenchus xylophilus* | 119.5625 | | 26.4792 |
| *Bursaphelenchus xylophilus* | 119.5625 | | 28.1042 |
| *Bursaphelenchus xylophilus* | 119.5625 | | 29.1042 |
| *Bursaphelenchus xylophilus* | 119.6042 | | 31.7292 |
| *Bursaphelenchus xylophilus* | 119.6458 | | 27.1042 |
| *Bursaphelenchus xylophilus* | 119.6458 | | 27.9792 |
| *Bursaphelenchus xylophilus* | 119.6875 | | 29.1042 |
| *Bursaphelenchus xylophilus* | 119.6875 | | 29.8125 |
| *Bursaphelenchus xylophilus* | 119.6875 | | 30.6458 |
| *Bursaphelenchus xylophilus* | 119.7292 | | 27.5625 |
| *Bursaphelenchus xylophilus* | 119.7292 | | 30.2292 |
| *Bursaphelenchus xylophilus* | 119.8125 | | 28.8958 |
| *Bursaphelenchus xylophilus* | 119.8125 | | 31.3542 |
| *Bursaphelenchus xylophilus* | 119.8958 | | 27.2292 |
| *Bursaphelenchus xylophilus* | 119.8958 | | 29.4792 |
| *Bursaphelenchus xylophilus* | 119.9375 | | 28.4375 |
| *Bursaphelenchus xylophilus* | 119.9375 | | 31.0208 |
| *Bursaphelenchus xylophilus* | 119.9792 | | 30.0625 |
| *Bursaphelenchus xylophilus* | 119.9792 | | 30.2708 |
| *Bursaphelenchus xylophilus* | 119.9792 | | 30.5625 |
| *Bursaphelenchus xylophilus* | 120.0208 | | 26.8958 |
| *Bursaphelenchus xylophilus* | 120.0625 | | 28.8958 |
| *Bursaphelenchus xylophilus* | 120.0625 | | 35.8958 |
| *Bursaphelenchus xylophilus* | 120.1042 | | 27.8125 |
| *Bursaphelenchus xylophilus* | 120.1042 | | 28.6458 |
| *Bursaphelenchus xylophilus* | 120.1458 | | 29.3542 |
| *Bursaphelenchus xylophilus* | 120.1875 | | 30.8542 |
| *Bursaphelenchus xylophilus* | 120.2292 | | 27.3125 |
| *Bursaphelenchus xylophilus* | 120.2292 | | 29.3125 |
| *Bursaphelenchus xylophilus* | 120.2708 | | 29.7292 |
| *Bursaphelenchus xylophilus* | 120.2708 | | 30.1875 |
| *Bursaphelenchus xylophilus* | 120.2708 | | 31.5208 |
| *Bursaphelenchus xylophilus* | 120.3125 | | 28.1458 |
| *Bursaphelenchus xylophilus* | 120.3125 | | 31.6875 |
| *Bursaphelenchus xylophilus* | 120.3958 | | 36.3125 |
| *Bursaphelenchus xylophilus* | 120.4375 | | 27.5208 |
| *Bursaphelenchus xylophilus* | 120.4375 | | 29.0625 |
| *Bursaphelenchus xylophilus* | 120.4375 | | 30.0625 |
| *Bursaphelenchus xylophilus* | 120.4375 | | 36.1458 |
| *Bursaphelenchus xylophilus* | 120.4375 | | 36.3958 |
| *Bursaphelenchus xylophilus* | 120.4792 | | 36.1042 |
| *Bursaphelenchus xylophilus* | 120.5625 | | 27.6042 |
| *Bursaphelenchus xylophilus* | 120.5625 | | 27.6875 |
| *Bursaphelenchus xylophilus* | 120.6042 | | 27.9792 |
| *Bursaphelenchus xylophilus* | 120.6042 | | 29.9792 |
| *Bursaphelenchus xylophilus* | 120.6458 | | 27.7708 |
| *Bursaphelenchus xylophilus* | 120.6458 | | 28.0208 |
| *Bursaphelenchus xylophilus* | 120.6875 | | 28.1458 |
| *Bursaphelenchus xylophilus* | 120.7292 | | 28.8542 |
| *Bursaphelenchus xylophilus* | 120.8125 | | 27.9375 |
| *Bursaphelenchus xylophilus* | 120.8542 | | 29.5625 |
| *Bursaphelenchus xylophilus* | 120.8542 | | 30.0208 |
| *Bursaphelenchus xylophilus* | 120.8542 | | 37.3542 |
| *Bursaphelenchus xylophilus* | 120.8958 | | 29.5208 |
| *Bursaphelenchus xylophilus* | 120.9792 | | 23.9792 |
| *Bursaphelenchus xylophilus* | 120.9792 | | 28.1042 |
| *Bursaphelenchus xylophilus* | 121.0208 | | 29.1458 |
| *Bursaphelenchus xylophilus* | 121.1458 | | 27.8542 |
| *Bursaphelenchus xylophilus* | 121.1458 | | 28.8542 |
| *Bursaphelenchus xylophilus* | 121.1458 | | 30.0625 |
| *Bursaphelenchus xylophilus* | 121.2292 | | 28.1458 |
| *Bursaphelenchus xylophilus* | 121.2708 | | 28.6458 |
| *Bursaphelenchus xylophilus* | 121.2708 | | 30.1875 |
| *Bursaphelenchus xylophilus* | 121.2708 | | 37.5208 |
| *Bursaphelenchus xylophilus* | 121.3958 | | 28.3958 |
| *Bursaphelenchus xylophilus* | 121.3958 | | 29.1042 |
| *Bursaphelenchus xylophilus* | 121.3958 | | 29.6458 |
| *Bursaphelenchus xylophilus* | 121.3958 | | 37.5625 |
| *Bursaphelenchus xylophilus* | 121.4375 | | 28.6875 |
| *Bursaphelenchus xylophilus* | 121.4375 | | 29.3125 |
| *Bursaphelenchus xylophilus* | 121.4375 | | 37.5208 |
| *Bursaphelenchus xylophilus* | 121.5208 | | 38.9375 |
| *Bursaphelenchus xylophilus* | 121.5625 | | 29.8125 |
| *Bursaphelenchus xylophilus* | 121.5625 | | 29.8542 |
| *Bursaphelenchus xylophilus* | 121.5625 | | 36.9375 |
| *Bursaphelenchus xylophilus* | 121.6042 | | 37.3958 |
| *Bursaphelenchus xylophilus* | 121.6042 | | 38.8958 |
| *Bursaphelenchus xylophilus* | 121.6042 | | 38.9375 |
| *Bursaphelenchus xylophilus* | 121.6458 | | 38.9375 |
| *Bursaphelenchus xylophilus* | 121.8542 | | 29.8958 |
| *Bursaphelenchus xylophilus* | 121.8958 | | 29.4792 |
| *Bursaphelenchus xylophilus* | 122.0625 | | 37.1875 |
| *Bursaphelenchus xylophilus* | 122.1042 | | 30.0208 |
| *Bursaphelenchus xylophilus* | 122.1458 | | 37.5208 |
| *Bursaphelenchus xylophilus* | 122.4792 | | 37.1875 |
| *Bursaphelenchus xylophilus* | 122.6042 | | 39.2708 |
| *Bursaphelenchus xylophilus* | 123.1042 | | 41.2292 |
| *Bursaphelenchus xylophilus* | 123.3542 | | 41.4375 |
| *Bursaphelenchus xylophilus* | 123.4375 | | 41.7292 |
| *Bursaphelenchus xylophilus* | 123.7292 | | 42.2292 |
| *Bursaphelenchus xylophilus* | 123.7708 | | 41.3542 |
| *Bursaphelenchus xylophilus* | 123.8125 | | 41.3125 |
| *Bursaphelenchus xylophilus* | 123.9375 | | 41.8958 |
| *Bursaphelenchus xylophilus* | 124.0625 | | 40.4792 |
| *Bursaphelenchus xylophilus* | 124.0625 | | 41.8542 |
| *Bursaphelenchus xylophilus* | 124.0625 | | 42.5625 |
| *Bursaphelenchus xylophilus* | 124.1458 | | 41.3125 |
| *Bursaphelenchus xylophilus* | 124.9375 | | 42.1042 |
| *Bursaphelenchus xylophilus* | 125.0625 | | 41.7292 |
| *Bursaphelenchus xylophilus* | 125.9375 | | 41.7292 |
| *Bursaphelenchus xylophilus* | 126.1042 | | 34.8542 |
| *Bursaphelenchus xylophilus* | 126.3958 | | 34.8125 |
| *Bursaphelenchus xylophilus* | 126.5208 | | 33.4375 |
| *Bursaphelenchus xylophilus* | 126.6875 | | 34.7708 |
| *Bursaphelenchus xylophilus* | 126.8542 | | 35.1875 |
| *Bursaphelenchus xylophilus* | 127.7292 | | 36.4792 |
| *Bursaphelenchus xylophilus* | 127.9792 | | 26.4792 |
| *Bursaphelenchus xylophilus* | 128.3542 | | 36.1042 |
| *Bursaphelenchus xylophilus* | 128.5625 | | 35.9375 |
| *Bursaphelenchus xylophilus* | 128.7292 | | 36.5625 |
| *Bursaphelenchus xylophilus* | 128.8542 | | 37.7708 |
| *Bursaphelenchus xylophilus* | 129.0625 | | 35.2708 |
| *Bursaphelenchus xylophilus* | 129.1042 | | 37.5208 |
| *Bursaphelenchus xylophilus* | 129.7708 | | 43.3125 |
| *Bursaphelenchus xylophilus* | 129.8958 | | 32.7708 |
| *Bursaphelenchus xylophilus* | 130.9792 | | 32.4375 |
| *Bursaphelenchus xylophilus* | 133.4792 | | 33.7292 |
| *Bursaphelenchus xylophilus* | 133.4792 | | 33.9375 |
| *Bursaphelenchus xylophilus* | 134.1042 | | 34.1875 |
| *Bursaphelenchus xylophilus* | 134.8125 | | 34.8542 |
| *Bursaphelenchus xylophilus* | 137.9792 | | 35.9792 |
| *Bursaphelenchus xylophilus* | 138.6042 | | 35.6458 |
| *Bursaphelenchus xylophilus* | 139.0208 | | 37.8958 |
| *Bursaphelenchus xylophilus* | 139.0625 | | 36.3958 |
| *Bursaphelenchus xylophilus* | 139.6458 | | 35.8958 |
| *Bursaphelenchus xylophilus* | 139.8125 | | 36.6875 |
| *Bursaphelenchus xylophilus* | 139.9375 | | 40.4375 |
| *Bursaphelenchus xylophilus* | 140.2292 | | 37.3958 |
| *Bursaphelenchus xylophilus* | 140.2708 | | 36.3125 |
| *Bursaphelenchus xylophilus* | 140.3542 | | 38.2708 |
| *Bursaphelenchus xylophilus* | 140.5625 | | 40.1458 |
| *Bursaphelenchus xylophilus* | 140.8542 | | 38.2708 |
| *Bursaphelenchus xylophilus* | 141.1458 | | 39.6875 |
| *Bursaphelenchus xylophilus* | 31.9792 | | 48.9792 |
| *Bursaphelenchus xylophilus* | 34.9792 | | 38.9792 |

**Table S2** The main parameter values of the 600 models for the FC and RM combinations

| Model | Mean_AUC_ratio | Omission_rate_at_5% | AICc | delta_AICc | num_parameters |
| --- | --- | --- | --- | --- | --- |
| M_0.1_F_l_Set_1 | 1.5847 | 0.0514 | 25270.3596 | 3185.1020 | 5 |
| M_0.1_F_q_Set_1 | 1.5147 | 0.0237 | 26840.9612 | 4755.7037 | 5 |
| M_0.1_F_p_Set_1 | 1.7530 | 0.0593 | 22827.2545 | 741.9970 | 9 |
| M_0.1_F_h_Set_1 | 1.9080 | 0.0474 | 22095.8058 | 10.5483 | 115 |
| M_0.1_F_lq_Set_1 | 1.9151 | 0.0277 | 22227.9443 | 142.6868 | 10 |
| M_0.1_F_lp_Set_1 | 1.8354 | 0.0435 | 22608.7052 | 523.4476 | 14 |
| M_0.1_F_lh_Set_1 | 1.9063 | 0.0474 | 22095.8058 | 10.5483 | 115 |
| M_0.1_F_qp_Set_1 | 1.9143 | 0.0316 | 22203.8003 | 118.5428 | 11 |
| M_0.1_F_qh_Set_1 | 1.9037 | 0.0474 | 22095.8058 | 10.5483 | 115 |
| M_0.1_F_ph_Set_1 | 1.9076 | 0.0474 | 22109.6733 | 24.4158 | 120 |
| M_0.1_F_lqp_Set_1 | 1.9192 | 0.0277 | 22168.3764 | 83.1188 | 16 |
| M_0.1_F_lqh_Set_1 | 1.9053 | 0.0474 | 22095.8058 | 10.5483 | 115 |
| M_0.1_F_lph_Set_1 | 1.9088 | 0.0474 | 22096.5767 | 11.3192 | 114 |
| M_0.1_F_qph_Set_1 | 1.9020 | 0.0474 | 22096.9828 | 11.7253 | 113 |
| M_0.1_F_lqph_Set_1 | 1.9076 | 0.0474 | 22096.9828 | 11.7253 | 113 |
| M_0.2_F_l_Set_1 | 1.5825 | 0.0514 | 25270.8205 | 3185.5629 | 5 |
| M_0.2_F_q_Set_1 | 1.5112 | 0.0237 | 26841.7088 | 4756.4513 | 5 |
| M_0.2_F_p_Set_1 | 1.7561 | 0.0593 | 22846.7742 | 761.5166 | 9 |
| M_0.2_F_h_Set_1 | 1.9022 | 0.0474 | 22098.0450 | 12.7875 | 102 |
| M_0.2_F_lq_Set_1 | 1.9084 | 0.0277 | 22321.6109 | 236.3533 | 9 |
| M_0.2_F_lp_Set_1 | 1.8294 | 0.0435 | 22625.1584 | 539.9009 | 13 |
| M_0.2_F_lh_Set_1 | 1.9076 | 0.0474 | 22098.0450 | 12.7875 | 102 |
| M_0.2_F_qp_Set_1 | 1.9181 | 0.0316 | 22235.4921 | 150.2345 | 10 |
| M_0.2_F_qh_Set_1 | 1.9077 | 0.0474 | 22098.0450 | 12.7875 | 102 |
| M_0.2_F_ph_Set_1 | 1.9034 | 0.0474 | 22107.6737 | 22.4162 | 104 |
| M_0.2_F_lqp_Set_1 | 1.9199 | 0.0277 | 22221.3642 | 136.1067 | 13 |
| M_0.2_F_lqh_Set_1 | 1.9060 | 0.0474 | 22098.0450 | 12.7875 | 102 |
| M_0.2_F_lph_Set_1 | 1.9043 | 0.0474 | 22121.2971 | 36.0396 | 112 |
| M_0.2_F_qph_Set_1 | 1.9028 | 0.0474 | 22090.5723 | 5.3148 | 99 |
| M_0.2_F_lqph_Set_1 | 1.9057 | 0.0474 | 22090.5723 | 5.3148 | 99 |
| M_0.3_F_l_Set_1 | 1.5785 | 0.0514 | 25271.3536 | 3186.0961 | 5 |
| M_0.3_F_q_Set_1 | 1.5075 | 0.0237 | 26843.1150 | 4757.8574 | 5 |
| M_0.3_F_p_Set_1 | 1.7441 | 0.0593 | 22865.9130 | 780.6555 | 9 |
| M_0.3_F_h_Set_1 | 1.9082 | 0.0474 | 22099.8293 | 14.5718 | 89 |
| M_0.3_F_lq_Set_1 | 1.9095 | 0.0316 | 22378.9683 | 293.7108 | 8 |
| M_0.3_F_lp_Set_1 | 1.8289 | 0.0435 | 22644.3852 | 559.1276 | 12 |
| M_0.3_F_lh_Set_1 | 1.9076 | 0.0474 | 22099.9966 | 14.7390 | 89 |
| M_0.3_F_qp_Set_1 | 1.9192 | 0.0316 | 22265.2751 | 180.0176 | 9 |
| M_0.3_F_qh_Set_1 | 1.9094 | 0.0474 | 22099.8293 | 14.5718 | 89 |
| M_0.3_F_ph_Set_1 | 1.9078 | 0.0474 | 22093.8997 | 8.6421 | 88 |
| M_0.3_F_lqp_Set_1 | 1.9208 | 0.0316 | 22267.5431 | 182.2855 | 13 |
| M_0.3_F_lqh_Set_1 | 1.9067 | 0.0474 | 22099.9966 | 14.7390 | 89 |
| M_0.3_F_lph_Set_1 | 1.9088 | 0.0474 | 22109.7297 | 24.4722 | 96 |
| M_0.3_F_qph_Set_1 | 1.9073 | 0.0474 | 22096.8248 | 11.5673 | 90 |
| M_0.3_F_lqph_Set_1 | 1.9065 | 0.0474 | 22086.9019 | 1.6444 | 85 |
| M_0.4_F_l_Set_1 | 1.5858 | 0.0514 | 25272.0050 | 3186.7474 | 5 |
| M_0.4_F_q_Set_1 | 1.5058 | 0.0237 | 26844.9830 | 4759.7255 | 5 |
| M_0.4_F_p_Set_1 | 1.7425 | 0.0593 | 22889.7950 | 804.5374 | 8 |
| M_0.4_F_h_Set_1 | 1.9122 | 0.0474 | 22132.1424 | 46.8849 | 94 |
| M_0.4_F_lq_Set_1 | 1.9097 | 0.0316 | 22413.2060 | 327.9485 | 7 |
| M_0.4_F_lp_Set_1 | 1.8353 | 0.0435 | 22658.7490 | 573.4914 | 11 |
| M_0.4_F_lh_Set_1 | 1.9087 | 0.0474 | 22132.1424 | 46.8849 | 94 |
| M_0.4_F_qp_Set_1 | 1.9159 | 0.0316 | 22295.4335 | 210.1760 | 9 |
| M_0.4_F_qh_Set_1 | 1.9099 | 0.0474 | 22132.1424 | 46.8849 | 94 |
| M_0.4_F_ph_Set_1 | 1.9096 | 0.0474 | 22106.5981 | 21.3406 | 86 |
| M_0.4_F_lqp_Set_1 | 1.9136 | 0.0316 | 22292.5779 | 207.3204 | 11 |
| M_0.4_F_lqh_Set_1 | 1.9058 | 0.0474 | 22132.1424 | 46.8849 | 94 |
| M_0.4_F_lph_Set_1 | 1.9087 | 0.0474 | 22107.6899 | 22.4324 | 87 |
| M_0.4_F_qph_Set_1 | 1.9110 | 0.0474 | 22088.4285 | 3.1709 | 79 |
| M_0.4_F_lqph_Set_1 | 1.9089 | 0.0474 | 22087.7688 | 2.5113 | 79 |
| M_0.5_F_l_Set_1 | 1.5723 | 0.0514 | 25272.6883 | 3187.4308 | 5 |
| M_0.5_F_q_Set_1 | 1.5035 | 0.0237 | 26847.1867 | 4761.9291 | 5 |
| M_0.5_F_p_Set_1 | 1.7360 | 0.0593 | 22908.0225 | 822.7649 | 8 |
| M_0.5_F_h_Set_1 | 1.9102 | 0.0474 | 22103.4338 | 18.1762 | 79 |
| M_0.5_F_lq_Set_1 | 1.9076 | 0.0356 | 22452.6465 | 367.3889 | 7 |
| M_0.5_F_lp_Set_1 | 1.8244 | 0.0435 | 22682.3107 | 597.0532 | 12 |
| M_0.5_F_lh_Set_1 | 1.9092 | 0.0474 | 22103.4338 | 18.1762 | 79 |
| M_0.5_F_qp_Set_1 | 1.9100 | 0.0316 | 22315.2478 | 229.9903 | 7 |
| M_0.5_F_qh_Set_1 | 1.9114 | 0.0474 | 22103.4338 | 18.1762 | 79 |
| M_0.5_F_ph_Set_1 | 1.9106 | 0.0474 | 22102.1311 | 16.8735 | 80 |
| M_0.5_F_lqp_Set_1 | 1.9142 | 0.0316 | 22313.7388 | 228.4813 | 9 |
| M_0.5_F_lqh_Set_1 | 1.9087 | 0.0474 | 22103.4338 | 18.1762 | 79 |
| M_0.5_F_lph_Set_1 | 1.9113 | 0.0474 | 22104.6420 | 19.3845 | 81 |
| M_0.5_F_qph_Set_1 | 1.9120 | 0.0474 | 22101.6338 | 16.3762 | 80 |
| M_0.5_F_lqph_Set_1 | 1.9113 | 0.0474 | 22103.0352 | 17.7776 | 81 |
| M_0.6_F_l_Set_1 | 1.5748 | 0.0514 | 25273.5893 | 3188.3317 | 5 |
| M_0.6_F_q_Set_1 | 1.5018 | 0.0277 | 26849.5705 | 4764.3130 | 5 |
| M_0.6_F_p_Set_1 | 1.7311 | 0.0593 | 22925.3840 | 840.1264 | 7 |
| M_0.6_F_h_Set_1 | 1.9106 | 0.0474 | 22117.3932 | 32.1356 | 81 |
| M_0.6_F_lq_Set_1 | 1.8993 | 0.0356 | 22491.8899 | 406.6324 | 8 |
| M_0.6_F_lp_Set_1 | 1.8289 | 0.0435 | 22706.6085 | 621.3510 | 11 |
| M_0.6_F_lh_Set_1 | 1.9103 | 0.0474 | 22117.3932 | 32.1356 | 81 |
| M_0.6_F_qp_Set_1 | 1.9075 | 0.0316 | 22339.2087 | 253.9512 | 8 |
| M_0.6_F_qh_Set_1 | 1.9115 | 0.0474 | 22127.8226 | 42.5650 | 85 |
| M_0.6_F_ph_Set_1 | 1.9121 | 0.0474 | 22095.3118 | 10.0543 | 74 |
| M_0.6_F_lqp_Set_1 | 1.9135 | 0.0316 | 22338.0017 | 252.7442 | 10 |
| M_0.6_F_lqh_Set_1 | 1.9092 | 0.0474 | 22127.8226 | 42.5650 | 85 |
| M_0.6_F_lph_Set_1 | 1.9101 | 0.0474 | 22107.9814 | 22.7238 | 79 |
| M_0.6_F_qph_Set_1 | 1.9091 | 0.0474 | 22085.2575 | 0.0000 | 70 |
| M_0.6_F_lqph_Set_1 | 1.9120 | 0.0474 | 22115.6589 | 30.4014 | 82 |
| M_0.7_F_l_Set_1 | 1.5780 | 0.0514 | 25274.5049 | 3189.2474 | 5 |
| M_0.7_F_q_Set_1 | 1.5013 | 0.0277 | 26852.0674 | 4766.8099 | 5 |
| M_0.7_F_p_Set_1 | 1.7194 | 0.0593 | 22940.9276 | 855.6700 | 7 |
| M_0.7_F_h_Set_1 | 1.9114 | 0.0514 | 22098.0048 | 12.7472 | 67 |
| M_0.7_F_lq_Set_1 | 1.9017 | 0.0356 | 22527.4500 | 442.1925 | 8 |
| M_0.7_F_lp_Set_1 | 1.8117 | 0.0435 | 22726.7556 | 641.4981 | 11 |
| M_0.7_F_lh_Set_1 | 1.9135 | 0.0514 | 22098.0048 | 12.7472 | 67 |
| M_0.7_F_qp_Set_1 | 1.9120 | 0.0356 | 22361.8070 | 276.5494 | 8 |
| M_0.7_F_qh_Set_1 | 1.9086 | 0.0514 | 22135.0144 | 49.7569 | 83 |
| M_0.7_F_ph_Set_1 | 1.9105 | 0.0514 | 22104.4559 | 19.1983 | 72 |
| M_0.7_F_lqp_Set_1 | 1.9118 | 0.0356 | 22367.4667 | 282.2092 | 10 |
| M_0.7_F_lqh_Set_1 | 1.9123 | 0.0514 | 22135.0144 | 49.7569 | 83 |
| M_0.7_F_lph_Set_1 | 1.9116 | 0.0514 | 22096.4123 | 11.1548 | 69 |
| M_0.7_F_qph_Set_1 | 1.9133 | 0.0474 | 22105.4138 | 20.1563 | 74 |
| M_0.7_F_lqph_Set_1 | 1.9108 | 0.0514 | 22090.3274 | 5.0698 | 66 |
| M_0.8_F_l_Set_1 | 1.5820 | 0.0514 | 25275.5641 | 3190.3066 | 5 |
| M_0.8_F_q_Set_1 | 1.5006 | 0.0277 | 26854.3762 | 4769.1187 | 5 |
| M_0.8_F_p_Set_1 | 1.7286 | 0.0593 | 22955.6449 | 870.3873 | 7 |
| M_0.8_F_h_Set_1 | 1.9126 | 0.0514 | 22105.7300 | 20.4724 | 66 |
| M_0.8_F_lq_Set_1 | 1.9022 | 0.0356 | 22563.2976 | 478.0400 | 7 |
| M_0.8_F_lp_Set_1 | 1.8100 | 0.0435 | 22754.1410 | 668.8835 | 11 |
| M_0.8_F_lh_Set_1 | 1.9103 | 0.0514 | 22105.7300 | 20.4724 | 66 |
| M_0.8_F_qp_Set_1 | 1.9081 | 0.0356 | 22385.1525 | 299.8950 | 8 |
| M_0.8_F_qh_Set_1 | 1.9137 | 0.0514 | 22105.7300 | 20.4724 | 66 |
| M_0.8_F_ph_Set_1 | 1.9139 | 0.0514 | 22093.1185 | 7.8610 | 64 |
| M_0.8_F_lqp_Set_1 | 1.9119 | 0.0356 | 22383.4582 | 298.2007 | 8 |
| M_0.8_F_lqh_Set_1 | 1.9109 | 0.0514 | 22105.7300 | 20.4724 | 66 |
| M_0.8_F_lph_Set_1 | 1.9114 | 0.0514 | 22099.1807 | 13.9232 | 66 |
| M_0.8_F_qph_Set_1 | 1.9117 | 0.0514 | 22098.3349 | 13.0773 | 68 |
| M_0.8_F_lqph_Set_1 | 1.9116 | 0.0514 | 22098.3349 | 13.0773 | 68 |
| M_0.9_F_l_Set_1 | 1.5694 | 0.0553 | 25276.6366 | 3191.3790 | 5 |
| M_0.9_F_q_Set_1 | 1.5014 | 0.0277 | 26856.5446 | 4771.2870 | 5 |
| M_0.9_F_p_Set_1 | 1.7356 | 0.0593 | 22971.5079 | 886.2504 | 7 |
| M_0.9_F_h_Set_1 | 1.9115 | 0.0514 | 22110.8361 | 25.5786 | 65 |
| M_0.9_F_lq_Set_1 | 1.8995 | 0.0395 | 22593.1329 | 507.8754 | 7 |
| M_0.9_F_lp_Set_1 | 1.8150 | 0.0435 | 22770.8317 | 685.5741 | 9 |
| M_0.9_F_lh_Set_1 | 1.9137 | 0.0474 | 22110.8361 | 25.5786 | 65 |
| M_0.9_F_qp_Set_1 | 1.9077 | 0.0356 | 22408.3179 | 323.0604 | 8 |
| M_0.9_F_qh_Set_1 | 1.9105 | 0.0514 | 22152.9899 | 67.7324 | 82 |
| M_0.9_F_ph_Set_1 | 1.9112 | 0.0435 | 22100.3449 | 15.0873 | 62 |
| M_0.9_F_lqp_Set_1 | 1.9053 | 0.0356 | 22406.9350 | 321.6774 | 8 |
| M_0.9_F_lqh_Set_1 | 1.9109 | 0.0474 | 22152.9899 | 67.7324 | 82 |
| M_0.9_F_lph_Set_1 | 1.9104 | 0.0514 | 22105.0506 | 19.7930 | 65 |
| M_0.9_F_qph_Set_1 | 1.9124 | 0.0474 | 22088.7272 | 3.4697 | 56 |
| M_0.9_F_lqph_Set_1 | 1.9113 | 0.0514 | 22090.2341 | 4.9765 | 58 |
| M_1_F_l_Set_1 | 1.5900 | 0.0553 | 25277.4939 | 3192.2364 | 5 |
| M_1_F_q_Set_1 | 1.5007 | 0.0277 | 26858.4798 | 4773.2223 | 5 |
| M_1_F_p_Set_1 | 1.7242 | 0.0593 | 22987.3626 | 902.1051 | 7 |
| M_1_F_h_Set_1 | 1.9124 | 0.0514 | 22100.1711 | 14.9136 | 56 |
| M_1_F_lq_Set_1 | 1.8923 | 0.0395 | 22623.1593 | 537.9017 | 6 |
| M_1_F_lp_Set_1 | 1.8142 | 0.0435 | 22794.7716 | 709.5141 | 9 |
| M_1_F_lh_Set_1 | 1.9124 | 0.0514 | 22100.1711 | 14.9136 | 56 |
| M_1_F_qp_Set_1 | 1.9079 | 0.0356 | 22429.1038 | 343.8463 | 8 |
| M_1_F_qh_Set_1 | 1.9149 | 0.0514 | 22100.1711 | 14.9136 | 56 |
| M_1_F_ph_Set_1 | 1.9129 | 0.0474 | 22098.6099 | 13.3523 | 57 |
| M_1_F_lqp_Set_1 | 1.9036 | 0.0356 | 22434.7483 | 349.4908 | 8 |
| M_1_F_lqh_Set_1 | 1.9117 | 0.0514 | 22100.1711 | 14.9136 | 56 |
| M_1_F_lph_Set_1 | 1.9096 | 0.0474 | 22105.7544 | 20.4968 | 60 |
| M_1_F_qph_Set_1 | 1.9120 | 0.0474 | 22104.1288 | 18.8713 | 60 |
| M_1_F_lqph_Set_1 | 1.9138 | 0.0474 | 22099.8870 | 14.6295 | 57 |
| M_1.1_F_l_Set_1 | 1.5810 | 0.0553 | 25279.1089 | 3193.8514 | 5 |
| M_1.1_F_q_Set_1 | 1.4997 | 0.0277 | 26860.1902 | 4774.9327 | 5 |
| M_1.1_F_p_Set_1 | 1.7331 | 0.0593 | 23002.8170 | 917.5595 | 7 |
| M_1.1_F_h_Set_1 | 1.9095 | 0.0514 | 22132.2770 | 47.0194 | 66 |
| M_1.1_F_lq_Set_1 | 1.8937 | 0.0435 | 22652.5036 | 567.2461 | 6 |
| M_1.1_F_lp_Set_1 | 1.8094 | 0.0435 | 22824.7253 | 739.4678 | 9 |
| M_1.1_F_lh_Set_1 | 1.9108 | 0.0474 | 22132.2770 | 47.0194 | 66 |
| M_1.1_F_qp_Set_1 | 1.9039 | 0.0356 | 22451.8394 | 366.5818 | 8 |
| M_1.1_F_qh_Set_1 | 1.9140 | 0.0514 | 22132.2770 | 47.0194 | 66 |
| M_1.1_F_ph_Set_1 | 1.9134 | 0.0474 | 22115.3456 | 30.0880 | 60 |
| M_1.1_F_lqp_Set_1 | 1.9058 | 0.0356 | 22453.2010 | 367.9435 | 8 |
| M_1.1_F_lqh_Set_1 | 1.9100 | 0.0474 | 22132.2770 | 47.0194 | 66 |
| M_1.1_F_lph_Set_1 | 1.9149 | 0.0474 | 22091.3897 | 6.1322 | 49 |
| M_1.1_F_qph_Set_1 | 1.9118 | 0.0474 | 22139.0864 | 53.8288 | 71 |
| M_1.1_F_lqph_Set_1 | 1.9115 | 0.0474 | 22139.0864 | 53.8288 | 71 |
| M_1.2_F_l_Set_1 | 1.5860 | 0.0553 | 25280.4389 | 3195.1814 | 5 |
| M_1.2_F_q_Set_1 | 1.5002 | 0.0277 | 26861.6114 | 4776.3539 | 5 |
| M_1.2_F_p_Set_1 | 1.7435 | 0.0593 | 23017.8413 | 932.5837 | 7 |
| M_1.2_F_h_Set_1 | 1.9142 | 0.0514 | 22133.1258 | 47.8683 | 65 |
| M_1.2_F_lq_Set_1 | 1.8877 | 0.0435 | 22679.5218 | 594.2642 | 6 |
| M_1.2_F_lp_Set_1 | 1.8032 | 0.0435 | 22849.0406 | 763.7831 | 9 |
| M_1.2_F_lh_Set_1 | 1.9132 | 0.0514 | 22133.1258 | 47.8683 | 65 |
| M_1.2_F_qp_Set_1 | 1.9054 | 0.0356 | 22474.9626 | 389.7051 | 8 |
| M_1.2_F_qh_Set_1 | 1.9105 | 0.0514 | 22133.1258 | 47.8683 | 65 |
| M_1.2_F_ph_Set_1 | 1.9124 | 0.0474 | 22124.7649 | 39.5074 | 61 |
| M_1.2_F_lqp_Set_1 | 1.9040 | 0.0356 | 22474.2006 | 388.9430 | 8 |
| M_1.2_F_lqh_Set_1 | 1.9103 | 0.0514 | 22133.1258 | 47.8683 | 65 |
| M_1.2_F_lph_Set_1 | 1.9120 | 0.0474 | 22124.7649 | 39.5074 | 61 |
| M_1.2_F_qph_Set_1 | 1.9115 | 0.0474 | 22111.0191 | 25.7616 | 54 |
| M_1.2_F_lqph_Set_1 | 1.9124 | 0.0474 | 22111.0191 | 25.7616 | 54 |
| M_1.3_F_l_Set_1 | 1.5852 | 0.0553 | 25281.8566 | 3196.5991 | 5 |
| M_1.3_F_q_Set_1 | 1.5012 | 0.0277 | 26860.4269 | 4775.1693 | 4 |
| M_1.3_F_p_Set_1 | 1.7235 | 0.0593 | 23034.1596 | 948.9021 | 7 |
| M_1.3_F_h_Set_1 | 1.9113 | 0.0514 | 22129.2785 | 44.0210 | 57 |
| M_1.3_F_lq_Set_1 | 1.8893 | 0.0435 | 22706.2924 | 621.0349 | 6 |
| M_1.3_F_lp_Set_1 | 1.7967 | 0.0435 | 22884.1059 | 798.8483 | 9 |
| M_1.3_F_lh_Set_1 | 1.9136 | 0.0514 | 22129.2785 | 44.0210 | 57 |
| M_1.3_F_qp_Set_1 | 1.9038 | 0.0356 | 22490.7000 | 405.4425 | 7 |
| M_1.3_F_qh_Set_1 | 1.9158 | 0.0514 | 22129.2785 | 44.0210 | 57 |
| M_1.3_F_ph_Set_1 | 1.9116 | 0.0474 | 22113.3301 | 28.0726 | 52 |
| M_1.3_F_lqp_Set_1 | 1.9010 | 0.0356 | 22495.1897 | 409.9322 | 7 |
| M_1.3_F_lqh_Set_1 | 1.9126 | 0.0514 | 22129.2785 | 44.0210 | 57 |
| M_1.3_F_lph_Set_1 | 1.9127 | 0.0474 | 22113.3301 | 28.0726 | 52 |
| M_1.3_F_qph_Set_1 | 1.9149 | 0.0474 | 22108.2073 | 22.9498 | 50 |
| M_1.3_F_lqph_Set_1 | 1.9123 | 0.0474 | 22108.2073 | 22.9498 | 50 |
| M_1.4_F_l_Set_1 | 1.5890 | 0.0553 | 25283.3383 | 3198.0808 | 5 |
| M_1.4_F_q_Set_1 | 1.5010 | 0.0277 | 26860.6263 | 4775.3688 | 4 |
| M_1.4_F_p_Set_1 | 1.7204 | 0.0593 | 23050.2961 | 965.0385 | 7 |
| M_1.4_F_h_Set_1 | 1.9118 | 0.0474 | 22141.8449 | 56.5874 | 59 |
| M_1.4_F_lq_Set_1 | 1.8893 | 0.0435 | 22729.3046 | 644.0471 | 6 |
| M_1.4_F_lp_Set_1 | 1.7969 | 0.0435 | 22897.8478 | 812.5903 | 8 |
| M_1.4_F_lh_Set_1 | 1.9109 | 0.0514 | 22141.8449 | 56.5874 | 59 |
| M_1.4_F_qp_Set_1 | 1.9004 | 0.0395 | 22519.4144 | 434.1569 | 7 |
| M_1.4_F_qh_Set_1 | 1.9097 | 0.0474 | 22141.8449 | 56.5874 | 59 |
| M_1.4_F_ph_Set_1 | 1.9130 | 0.0474 | 22137.5782 | 52.3206 | 59 |
| M_1.4_F_lqp_Set_1 | 1.8988 | 0.0395 | 22511.7018 | 426.4443 | 7 |
| M_1.4_F_lqh_Set_1 | 1.9131 | 0.0514 | 22141.8449 | 56.5874 | 59 |
| M_1.4_F_lph_Set_1 | 1.9122 | 0.0474 | 22127.0994 | 41.8419 | 54 |
| M_1.4_F_qph_Set_1 | 1.9130 | 0.0474 | 22116.8249 | 31.5674 | 49 |
| M_1.4_F_lqph_Set_1 | 1.9108 | 0.0474 | 22111.0501 | 25.7925 | 47 |
| M_1.5_F_l_Set_1 | 1.5834 | 0.0553 | 25284.8750 | 3199.6175 | 5 |
| M_1.5_F_q_Set_1 | 1.5009 | 0.0277 | 26860.8338 | 4775.5762 | 4 |
| M_1.5_F_p_Set_1 | 1.7279 | 0.0593 | 23066.3623 | 981.1047 | 7 |
| M_1.5_F_h_Set_1 | 1.9130 | 0.0474 | 22154.0464 | 68.7888 | 61 |
| M_1.5_F_lq_Set_1 | 1.8794 | 0.0435 | 22759.6323 | 674.3748 | 6 |
| M_1.5_F_lp_Set_1 | 1.7974 | 0.0435 | 22911.6916 | 826.4341 | 8 |
| M_1.5_F_lh_Set_1 | 1.9115 | 0.0474 | 22154.0464 | 68.7888 | 61 |
| M_1.5_F_qp_Set_1 | 1.8960 | 0.0395 | 22535.0595 | 449.8019 | 7 |
| M_1.5_F_qh_Set_1 | 1.9115 | 0.0474 | 22154.0464 | 68.7888 | 61 |
| M_1.5_F_ph_Set_1 | 1.9109 | 0.0474 | 22115.9564 | 30.6989 | 46 |
| M_1.5_F_lqp_Set_1 | 1.9025 | 0.0395 | 22534.5397 | 449.2821 | 7 |
| M_1.5_F_lqh_Set_1 | 1.9098 | 0.0474 | 22154.0464 | 68.7888 | 61 |
| M_1.5_F_lph_Set_1 | 1.9121 | 0.0474 | 22115.9564 | 30.6989 | 46 |
| M_1.5_F_qph_Set_1 | 1.9108 | 0.0474 | 22123.1295 | 37.8720 | 48 |
| M_1.5_F_lqph_Set_1 | 1.9143 | 0.0474 | 22123.1295 | 37.8720 | 48 |
| M_1.6_F_l_Set_1 | 1.5839 | 0.0553 | 25286.5029 | 3201.2454 | 5 |
| M_1.6_F_q_Set_1 | 1.5005 | 0.0237 | 26861.0480 | 4775.7905 | 4 |
| M_1.6_F_p_Set_1 | 1.7098 | 0.0593 | 23082.4118 | 997.1542 | 7 |
| M_1.6_F_h_Set_1 | 1.9112 | 0.0474 | 22169.7801 | 84.5226 | 64 |
| M_1.6_F_lq_Set_1 | 1.8860 | 0.0435 | 22783.7415 | 698.4839 | 6 |
| M_1.6_F_lp_Set_1 | 1.7913 | 0.0435 | 22925.8898 | 840.6323 | 8 |
| M_1.6_F_lh_Set_1 | 1.9116 | 0.0474 | 22171.3358 | 86.0782 | 65 |
| M_1.6_F_qp_Set_1 | 1.9037 | 0.0435 | 22553.9246 | 468.6670 | 7 |
| M_1.6_F_qh_Set_1 | 1.9111 | 0.0474 | 22173.2135 | 87.9559 | 66 |
| M_1.6_F_ph_Set_1 | 1.9144 | 0.0474 | 22154.2793 | 69.0218 | 60 |
| M_1.6_F_lqp_Set_1 | 1.8979 | 0.0435 | 22557.5705 | 472.3129 | 7 |
| M_1.6_F_lqh_Set_1 | 1.9132 | 0.0474 | 22173.2135 | 87.9559 | 66 |
| M_1.6_F_lph_Set_1 | 1.9138 | 0.0474 | 22154.2793 | 69.0218 | 60 |
| M_1.6_F_qph_Set_1 | 1.9133 | 0.0474 | 22142.4268 | 57.1693 | 54 |
| M_1.6_F_lqph_Set_1 | 1.9117 | 0.0474 | 22142.4268 | 57.1693 | 54 |
| M_1.7_F_l_Set_1 | 1.5813 | 0.0553 | 25287.8408 | 3202.5832 | 5 |
| M_1.7_F_q_Set_1 | 1.5015 | 0.0237 | 26861.2694 | 4776.0119 | 4 |
| M_1.7_F_p_Set_1 | 1.7186 | 0.0593 | 23095.7551 | 1010.4976 | 6 |
| M_1.7_F_h_Set_1 | 1.9096 | 0.0474 | 22174.5939 | 89.3363 | 64 |
| M_1.7_F_lq_Set_1 | 1.8806 | 0.0435 | 22804.8316 | 719.5740 | 5 |
| M_1.7_F_lp_Set_1 | 1.7882 | 0.0435 | 22934.9078 | 849.6503 | 7 |
| M_1.7_F_lh_Set_1 | 1.9109 | 0.0474 | 22191.4137 | 106.1561 | 71 |
| M_1.7_F_qp_Set_1 | 1.8998 | 0.0435 | 22575.5841 | 490.3266 | 8 |
| M_1.7_F_qh_Set_1 | 1.9102 | 0.0474 | 22164.5035 | 79.2460 | 59 |
| M_1.7_F_ph_Set_1 | 1.9136 | 0.0474 | 22124.6686 | 39.4111 | 44 |
| M_1.7_F_lqp_Set_1 | 1.8973 | 0.0435 | 22575.8722 | 490.6146 | 8 |
| M_1.7_F_lqh_Set_1 | 1.9097 | 0.0474 | 22164.5035 | 79.2460 | 59 |
| M_1.7_F_lph_Set_1 | 1.9146 | 0.0474 | 22124.6686 | 39.4111 | 44 |
| M_1.7_F_qph_Set_1 | 1.9117 | 0.0474 | 22154.4104 | 69.1529 | 57 |
| M_1.7_F_lqph_Set_1 | 1.9117 | 0.0474 | 22154.4104 | 69.1529 | 57 |
| M_1.8_F_l_Set_1 | 1.5833 | 0.0553 | 25289.6312 | 3204.3737 | 5 |
| M_1.8_F_q_Set_1 | 1.5019 | 0.0237 | 26861.4979 | 4776.2404 | 4 |
| M_1.8_F_p_Set_1 | 1.7120 | 0.0593 | 23110.9688 | 1025.7112 | 6 |
| M_1.8_F_h_Set_1 | 1.9142 | 0.0474 | 22179.3286 | 94.0711 | 62 |
| M_1.8_F_lq_Set_1 | 1.8714 | 0.0435 | 22826.4100 | 741.1525 | 5 |
| M_1.8_F_lp_Set_1 | 1.7866 | 0.0435 | 22948.7415 | 863.4839 | 7 |
| M_1.8_F_lh_Set_1 | 1.9123 | 0.0474 | 22185.7597 | 100.5022 | 66 |
| M_1.8_F_qp_Set_1 | 1.8928 | 0.0435 | 22600.5145 | 515.2570 | 8 |
| M_1.8_F_qh_Set_1 | 1.9116 | 0.0474 | 22191.4849 | 106.2274 | 67 |
| M_1.8_F_ph_Set_1 | 1.9137 | 0.0474 | 22139.9586 | 54.7011 | 48 |
| M_1.8_F_lqp_Set_1 | 1.8965 | 0.0435 | 22597.6586 | 512.4011 | 8 |
| M_1.8_F_lqh_Set_1 | 1.9096 | 0.0474 | 22192.4290 | 107.1714 | 67 |
| M_1.8_F_lph_Set_1 | 1.9129 | 0.0474 | 22139.9586 | 54.7011 | 48 |
| M_1.8_F_qph_Set_1 | 1.9106 | 0.0474 | 22152.3142 | 67.0567 | 53 |
| M_1.8_F_lqph_Set_1 | 1.9128 | 0.0474 | 22152.3142 | 67.0567 | 53 |
| M_1.9_F_l_Set_1 | 1.5851 | 0.0514 | 25291.5334 | 3206.2759 | 5 |
| M_1.9_F_q_Set_1 | 1.5019 | 0.0237 | 26861.7328 | 4776.4753 | 4 |
| M_1.9_F_p_Set_1 | 1.7164 | 0.0593 | 23126.2239 | 1040.9664 | 6 |
| M_1.9_F_h_Set_1 | 1.9106 | 0.0474 | 22184.6499 | 99.3924 | 62 |
| M_1.9_F_lq_Set_1 | 1.8750 | 0.0435 | 22848.3245 | 763.0670 | 5 |
| M_1.9_F_lp_Set_1 | 1.8010 | 0.0474 | 22961.7425 | 876.4849 | 7 |
| M_1.9_F_lh_Set_1 | 1.9113 | 0.0474 | 22184.6499 | 99.3924 | 62 |
| M_1.9_F_qp_Set_1 | 1.8954 | 0.0435 | 22615.6466 | 530.3891 | 8 |
| M_1.9_F_qh_Set_1 | 1.9099 | 0.0474 | 22169.9454 | 84.6879 | 54 |
| M_1.9_F_ph_Set_1 | 1.9109 | 0.0474 | 22133.3073 | 48.0498 | 42 |
| M_1.9_F_lqp_Set_1 | 1.8947 | 0.0435 | 22618.9903 | 533.7327 | 8 |
| M_1.9_F_lqh_Set_1 | 1.9134 | 0.0474 | 22177.7310 | 92.4735 | 58 |
| M_1.9_F_lph_Set_1 | 1.9122 | 0.0474 | 22137.2609 | 52.0034 | 45 |
| M_1.9_F_qph_Set_1 | 1.9139 | 0.0474 | 22150.0948 | 64.8372 | 50 |
| M_1.9_F_lqph_Set_1 | 1.9120 | 0.0474 | 22150.0948 | 64.8372 | 50 |
| M_2_F_l_Set_1 | 1.5954 | 0.0514 | 25293.2771 | 3208.0195 | 5 |
| M_2_F_q_Set_1 | 1.5006 | 0.0237 | 26861.9741 | 4776.7166 | 4 |
| M_2_F_p_Set_1 | 1.7174 | 0.0593 | 23138.0500 | 1052.7924 | 5 |
| M_2_F_h_Set_1 | 1.9103 | 0.0474 | 22197.2185 | 111.9610 | 63 |
| M_2_F_lq_Set_1 | 1.8769 | 0.0435 | 22869.2146 | 783.9571 | 5 |
| M_2_F_lp_Set_1 | 1.7832 | 0.0553 | 22976.2795 | 891.0219 | 7 |
| M_2_F_lh_Set_1 | 1.9107 | 0.0474 | 22183.9060 | 98.6484 | 57 |
| M_2_F_qp_Set_1 | 1.8957 | 0.0435 | 22638.1078 | 552.8503 | 8 |
| M_2_F_qh_Set_1 | 1.9094 | 0.0474 | 22211.7197 | 126.4622 | 69 |
| M_2_F_ph_Set_1 | 1.9113 | 0.0474 | 22159.2531 | 73.9955 | 50 |
| M_2_F_lqp_Set_1 | 1.8942 | 0.0435 | 22638.7921 | 553.5346 | 8 |
| M_2_F_lqh_Set_1 | 1.9128 | 0.0474 | 22203.0922 | 117.8347 | 66 |
| M_2_F_lph_Set_1 | 1.9152 | 0.0474 | 22159.2531 | 73.9955 | 50 |
| M_2_F_qph_Set_1 | 1.9133 | 0.0474 | 22148.6062 | 63.3487 | 46 |
| M_2_F_lqph_Set_1 | 1.9123 | 0.0474 | 22148.6062 | 63.3487 | 46 |
| M_2.1_F_l_Set_1 | 1.5811 | 0.0514 | 25295.1188 | 3209.8613 | 5 |
| M_2.1_F_q_Set_1 | 1.5007 | 0.0237 | 26862.2217 | 4776.9642 | 4 |
| M_2.1_F_p_Set_1 | 1.7317 | 0.0593 | 23150.8617 | 1065.6042 | 5 |
| M_2.1_F_h_Set_1 | 1.9107 | 0.0474 | 22177.4213 | 92.1638 | 51 |
| M_2.1_F_lq_Set_1 | 1.8703 | 0.0474 | 22889.2071 | 803.9496 | 5 |
| M_2.1_F_lp_Set_1 | 1.7884 | 0.0553 | 22989.6887 | 904.4311 | 7 |
| M_2.1_F_lh_Set_1 | 1.9093 | 0.0474 | 22192.3922 | 107.1347 | 58 |
| M_2.1_F_qp_Set_1 | 1.8945 | 0.0435 | 22658.1808 | 572.9233 | 8 |
| M_2.1_F_qh_Set_1 | 1.9095 | 0.0474 | 22181.2536 | 95.9961 | 55 |
| M_2.1_F_ph_Set_1 | 1.9094 | 0.0474 | 22159.7826 | 74.5251 | 48 |
| M_2.1_F_lqp_Set_1 | 1.8943 | 0.0435 | 22659.6822 | 574.4247 | 8 |
| M_2.1_F_lqh_Set_1 | 1.9117 | 0.0474 | 22188.9959 | 103.7383 | 56 |
| M_2.1_F_lph_Set_1 | 1.9130 | 0.0474 | 22159.7826 | 74.5251 | 48 |
| M_2.1_F_qph_Set_1 | 1.9117 | 0.0474 | 22152.2264 | 66.9689 | 44 |
| M_2.1_F_lqph_Set_1 | 1.9116 | 0.0474 | 22152.2264 | 66.9689 | 44 |
| M_2.2_F_l_Set_1 | 1.5826 | 0.0514 | 25297.0158 | 3211.7583 | 5 |
| M_2.2_F_q_Set_1 | 1.5011 | 0.0237 | 26862.4758 | 4777.2183 | 4 |
| M_2.2_F_p_Set_1 | 1.7155 | 0.0593 | 23163.8722 | 1078.6147 | 5 |
| M_2.2_F_h_Set_1 | 1.9087 | 0.0474 | 22179.7404 | 94.4828 | 51 |
| M_2.2_F_lq_Set_1 | 1.8752 | 0.0474 | 22911.8733 | 826.6158 | 5 |
| M_2.2_F_lp_Set_1 | 1.7907 | 0.0553 | 23002.6632 | 917.4056 | 7 |
| M_2.2_F_lh_Set_1 | 1.9094 | 0.0474 | 22181.7521 | 96.4945 | 50 |
| M_2.2_F_qp_Set_1 | 1.8868 | 0.0435 | 22679.9490 | 594.6915 | 8 |
| M_2.2_F_qh_Set_1 | 1.9087 | 0.0474 | 22216.8274 | 131.5699 | 66 |
| M_2.2_F_ph_Set_1 | 1.9129 | 0.0474 | 22175.1017 | 89.8442 | 52 |
| M_2.2_F_lqp_Set_1 | 1.8923 | 0.0435 | 22678.9183 | 593.6608 | 8 |
| M_2.2_F_lqh_Set_1 | 1.9136 | 0.0474 | 22205.8079 | 120.5503 | 61 |
| M_2.2_F_lph_Set_1 | 1.9124 | 0.0474 | 22175.1017 | 89.8442 | 52 |
| M_2.2_F_qph_Set_1 | 1.9137 | 0.0474 | 22161.5683 | 76.3107 | 46 |
| M_2.2_F_lqph_Set_1 | 1.9134 | 0.0474 | 22161.5683 | 76.3107 | 46 |
| M_2.3_F_l_Set_1 | 1.5778 | 0.0514 | 25299.1241 | 3213.8665 | 5 |
| M_2.3_F_q_Set_1 | 1.5009 | 0.0237 | 26862.7355 | 4777.4780 | 4 |
| M_2.3_F_p_Set_1 | 1.7099 | 0.0593 | 23176.6031 | 1091.3455 | 5 |
| M_2.3_F_h_Set_1 | 1.9072 | 0.0474 | 22203.9178 | 118.6603 | 56 |
| M_2.3_F_lq_Set_1 | 1.8721 | 0.0474 | 22933.0291 | 847.7716 | 5 |
| M_2.3_F_lp_Set_1 | 1.7917 | 0.0553 | 23015.6827 | 930.4251 | 7 |
| M_2.3_F_lh_Set_1 | 1.9101 | 0.0474 | 22200.6922 | 115.4347 | 55 |
| M_2.3_F_qp_Set_1 | 1.8867 | 0.0435 | 22698.7202 | 613.4626 | 8 |
| M_2.3_F_qh_Set_1 | 1.9066 | 0.0474 | 22207.6335 | 122.3760 | 59 |
| M_2.3_F_ph_Set_1 | 1.9109 | 0.0474 | 22174.1934 | 88.9359 | 48 |
| M_2.3_F_lqp_Set_1 | 1.8874 | 0.0435 | 22699.4596 | 614.2021 | 8 |
| M_2.3_F_lqh_Set_1 | 1.9106 | 0.0474 | 22198.6269 | 113.3694 | 55 |
| M_2.3_F_lph_Set_1 | 1.9104 | 0.0474 | 22180.8928 | 95.6353 | 51 |
| M_2.3_F_qph_Set_1 | 1.9113 | 0.0474 | 22165.0120 | 79.7544 | 44 |
| M_2.3_F_lqph_Set_1 | 1.9113 | 0.0474 | 22165.0120 | 79.7544 | 44 |
| M_2.4_F_l_Set_1 | 1.5820 | 0.0514 | 25301.0037 | 3215.7461 | 5 |
| M_2.4_F_q_Set_1 | 1.5004 | 0.0237 | 26863.0015 | 4777.7439 | 4 |
| M_2.4_F_p_Set_1 | 1.7159 | 0.0593 | 23189.3827 | 1104.1252 | 5 |
| M_2.4_F_h_Set_1 | 1.9090 | 0.0474 | 22200.7393 | 115.4818 | 52 |
| M_2.4_F_lq_Set_1 | 1.8732 | 0.0474 | 22954.3335 | 869.0760 | 5 |
| M_2.4_F_lp_Set_1 | 1.7930 | 0.0553 | 23026.1551 | 940.8975 | 7 |
| M_2.4_F_lh_Set_1 | 1.9080 | 0.0474 | 22199.1567 | 113.8991 | 52 |
| M_2.4_F_qp_Set_1 | 1.8901 | 0.0435 | 22718.4153 | 633.1578 | 8 |
| M_2.4_F_qh_Set_1 | 1.9102 | 0.0474 | 22215.6076 | 130.3500 | 60 |
| M_2.4_F_ph_Set_1 | 1.9108 | 0.0474 | 22178.3145 | 93.0569 | 47 |
| M_2.4_F_lqp_Set_1 | 1.8824 | 0.0435 | 22719.3224 | 634.0649 | 8 |
| M_2.4_F_lqh_Set_1 | 1.9125 | 0.0474 | 22209.7584 | 124.5008 | 57 |
| M_2.4_F_lph_Set_1 | 1.9137 | 0.0474 | 22180.4606 | 95.2030 | 48 |
| M_2.4_F_qph_Set_1 | 1.9133 | 0.0474 | 22195.7110 | 110.4535 | 55 |
| M_2.4_F_lqph_Set_1 | 1.9104 | 0.0474 | 22195.7110 | 110.4535 | 55 |
| M_2.5_F_l_Set_1 | 1.5836 | 0.0514 | 25300.2811 | 3215.0236 | 4 |
| M_2.5_F_q_Set_1 | 1.5010 | 0.0237 | 26863.2727 | 4778.0151 | 4 |
| M_2.5_F_p_Set_1 | 1.7127 | 0.0593 | 23202.3119 | 1117.0544 | 5 |
| M_2.5_F_h_Set_1 | 1.9098 | 0.0474 | 22210.3734 | 125.1159 | 54 |
| M_2.5_F_lq_Set_1 | 1.8743 | 0.0474 | 22975.3455 | 890.0880 | 5 |
| M_2.5_F_lp_Set_1 | 1.7865 | 0.0553 | 23040.1629 | 954.9053 | 7 |
| M_2.5_F_lh_Set_1 | 1.9100 | 0.0474 | 22212.8440 | 127.5865 | 55 |
| M_2.5_F_qp_Set_1 | 1.8869 | 0.0435 | 22738.7203 | 653.4628 | 8 |
| M_2.5_F_qh_Set_1 | 1.9079 | 0.0474 | 22239.4333 | 154.1757 | 66 |
| M_2.5_F_ph_Set_1 | 1.9121 | 0.0474 | 22189.7977 | 104.5402 | 50 |
| M_2.5_F_lqp_Set_1 | 1.8796 | 0.0435 | 22740.4104 | 655.1529 | 8 |
| M_2.5_F_lqh_Set_1 | 1.9090 | 0.0474 | 22218.3102 | 133.0527 | 58 |
| M_2.5_F_lph_Set_1 | 1.9097 | 0.0474 | 22191.3269 | 106.0693 | 49 |
| M_2.5_F_qph_Set_1 | 1.9170 | 0.0474 | 22198.2119 | 112.9544 | 54 |
| M_2.5_F_lqph_Set_1 | 1.9102 | 0.0474 | 22198.2119 | 112.9544 | 54 |
| M_2.6_F_l_Set_1 | 1.5803 | 0.0514 | 25301.3165 | 3216.0589 | 4 |
| M_2.6_F_q_Set_1 | 1.5033 | 0.0237 | 26863.5500 | 4778.2925 | 4 |
| M_2.6_F_p_Set_1 | 1.7164 | 0.0593 | 23215.1536 | 1129.8960 | 5 |
| M_2.6_F_h_Set_1 | 1.9102 | 0.0474 | 22212.1352 | 126.8777 | 52 |
| M_2.6_F_lq_Set_1 | 1.8670 | 0.0474 | 22995.1464 | 909.8888 | 5 |
| M_2.6_F_lp_Set_1 | 1.7996 | 0.0553 | 23054.0744 | 968.8169 | 7 |
| M_2.6_F_lh_Set_1 | 1.9118 | 0.0474 | 22213.0815 | 127.8240 | 51 |
| M_2.6_F_qp_Set_1 | 1.8836 | 0.0435 | 22757.0588 | 671.8013 | 8 |
| M_2.6_F_qh_Set_1 | 1.9091 | 0.0474 | 22212.1352 | 126.8777 | 52 |
| M_2.6_F_ph_Set_1 | 1.9090 | 0.0474 | 22189.6574 | 104.3999 | 46 |
| M_2.6_F_lqp_Set_1 | 1.8834 | 0.0435 | 22756.5014 | 671.2439 | 8 |
| M_2.6_F_lqh_Set_1 | 1.9089 | 0.0474 | 22213.0815 | 127.8240 | 51 |
| M_2.6_F_lph_Set_1 | 1.9106 | 0.0474 | 22197.6867 | 112.4292 | 50 |
| M_2.6_F_qph_Set_1 | 1.9121 | 0.0474 | 22197.8740 | 112.6164 | 51 |
| M_2.6_F_lqph_Set_1 | 1.9097 | 0.0474 | 22197.8740 | 112.6164 | 51 |
| M_2.7_F_l_Set_1 | 1.5804 | 0.0514 | 25302.3663 | 3217.1088 | 4 |
| M_2.7_F_q_Set_1 | 1.5020 | 0.0237 | 26863.8326 | 4778.5751 | 4 |
| M_2.7_F_p_Set_1 | 1.6965 | 0.0593 | 23227.8792 | 1142.6216 | 5 |
| M_2.7_F_h_Set_1 | 1.9087 | 0.0474 | 22208.7983 | 123.5408 | 47 |
| M_2.7_F_lq_Set_1 | 1.8728 | 0.0474 | 23016.0978 | 930.8403 | 5 |
| M_2.7_F_lp_Set_1 | 1.7909 | 0.0553 | 23066.5924 | 981.3349 | 7 |
| M_2.7_F_lh_Set_1 | 1.9070 | 0.0474 | 22227.5693 | 142.3117 | 55 |
| M_2.7_F_qp_Set_1 | 1.8751 | 0.0435 | 22776.8967 | 691.6392 | 8 |
| M_2.7_F_qh_Set_1 | 1.9087 | 0.0474 | 22208.7983 | 123.5408 | 47 |
| M_2.7_F_ph_Set_1 | 1.9107 | 0.0474 | 22185.7429 | 100.4853 | 43 |
| M_2.7_F_lqp_Set_1 | 1.8828 | 0.0435 | 22776.0397 | 690.7822 | 8 |
| M_2.7_F_lqh_Set_1 | 1.9094 | 0.0474 | 22227.5693 | 142.3117 | 55 |
| M_2.7_F_lph_Set_1 | 1.9102 | 0.0474 | 22210.8377 | 125.5802 | 53 |
| M_2.7_F_qph_Set_1 | 1.9123 | 0.0474 | 22203.7795 | 118.5220 | 50 |
| M_2.7_F_lqph_Set_1 | 1.9106 | 0.0474 | 22203.7795 | 118.5220 | 50 |
| M_2.8_F_l_Set_1 | 1.5934 | 0.0514 | 25303.4300 | 3218.1725 | 4 |
| M_2.8_F_q_Set_1 | 1.5019 | 0.0237 | 26864.1202 | 4778.8626 | 4 |
| M_2.8_F_p_Set_1 | 1.7030 | 0.0593 | 23240.6456 | 1155.3881 | 5 |
| M_2.8_F_h_Set_1 | 1.9080 | 0.0435 | 22219.3964 | 134.1388 | 48 |
| M_2.8_F_lq_Set_1 | 1.8709 | 0.0474 | 23036.4427 | 951.1851 | 5 |
| M_2.8_F_lp_Set_1 | 1.7939 | 0.0553 | 23081.9029 | 996.6453 | 7 |
| M_2.8_F_lh_Set_1 | 1.9068 | 0.0474 | 22212.8179 | 127.5604 | 45 |
| M_2.8_F_qp_Set_1 | 1.8768 | 0.0435 | 22795.3475 | 710.0899 | 8 |
| M_2.8_F_qh_Set_1 | 1.9095 | 0.0435 | 22219.3964 | 134.1388 | 48 |
| M_2.8_F_ph_Set_1 | 1.9137 | 0.0474 | 22205.3280 | 120.0704 | 48 |
| M_2.8_F_lqp_Set_1 | 1.8719 | 0.0435 | 22795.1553 | 709.8977 | 8 |
| M_2.8_F_lqh_Set_1 | 1.9062 | 0.0474 | 22212.8179 | 127.5604 | 45 |
| M_2.8_F_lph_Set_1 | 1.9068 | 0.0474 | 22205.3280 | 120.0704 | 48 |
| M_2.8_F_qph_Set_1 | 1.9100 | 0.0474 | 22209.0313 | 123.7738 | 50 |
| M_2.8_F_lqph_Set_1 | 1.9092 | 0.0474 | 22209.0313 | 123.7738 | 50 |
| M_2.9_F_l_Set_1 | 1.5795 | 0.0514 | 25304.5075 | 3219.2500 | 4 |
| M_2.9_F_q_Set_1 | 1.5015 | 0.0237 | 26864.4137 | 4779.1561 | 4 |
| M_2.9_F_p_Set_1 | 1.7045 | 0.0593 | 23253.6188 | 1168.3613 | 5 |
| M_2.9_F_h_Set_1 | 1.9063 | 0.0435 | 22218.2722 | 133.0147 | 46 |
| M_2.9_F_lq_Set_1 | 1.8719 | 0.0474 | 23056.7781 | 971.5206 | 5 |
| M_2.9_F_lp_Set_1 | 1.7877 | 0.0553 | 23089.9604 | 1004.7029 | 7 |
| M_2.9_F_lh_Set_1 | 1.9112 | 0.0474 | 22217.1150 | 131.8575 | 44 |
| M_2.9_F_qp_Set_1 | 1.8783 | 0.0435 | 22812.9009 | 727.6434 | 8 |
| M_2.9_F_qh_Set_1 | 1.9095 | 0.0435 | 22218.2722 | 133.0147 | 46 |
| M_2.9_F_ph_Set_1 | 1.9100 | 0.0474 | 22194.9452 | 109.6877 | 41 |
| M_2.9_F_lqp_Set_1 | 1.8694 | 0.0435 | 22813.8583 | 728.6007 | 8 |
| M_2.9_F_lqh_Set_1 | 1.9095 | 0.0474 | 22217.1150 | 131.8575 | 44 |
| M_2.9_F_lph_Set_1 | 1.9094 | 0.0474 | 22194.9452 | 109.6877 | 41 |
| M_2.9_F_qph_Set_1 | 1.9113 | 0.0474 | 22215.2374 | 129.9799 | 50 |
| M_2.9_F_lqph_Set_1 | 1.9100 | 0.0474 | 22215.2374 | 129.9799 | 50 |
| M_3_F_l_Set_1 | 1.5823 | 0.0514 | 25305.5983 | 3220.3408 | 4 |
| M_3_F_q_Set_1 | 1.5017 | 0.0237 | 26864.7118 | 4779.4543 | 4 |
| M_3_F_p_Set_1 | 1.7065 | 0.0593 | 23266.1986 | 1180.9410 | 5 |
| M_3_F_h_Set_1 | 1.9041 | 0.0435 | 22229.1218 | 143.8642 | 47 |
| M_3_F_lq_Set_1 | 1.8690 | 0.0474 | 23078.7586 | 993.5010 | 6 |
| M_3_F_lp_Set_1 | 1.7929 | 0.0553 | 23103.4945 | 1018.2370 | 7 |
| M_3_F_lh_Set_1 | 1.9098 | 0.0474 | 22224.6732 | 139.4157 | 44 |
| M_3_F_qp_Set_1 | 1.8742 | 0.0435 | 22831.1104 | 745.8529 | 8 |
| M_3_F_qh_Set_1 | 1.9061 | 0.0435 | 22229.1218 | 143.8642 | 47 |
| M_3_F_ph_Set_1 | 1.9112 | 0.0474 | 22229.8298 | 144.5723 | 52 |
| M_3_F_lqp_Set_1 | 1.8727 | 0.0435 | 22831.4606 | 746.2031 | 8 |
| M_3_F_lqh_Set_1 | 1.9095 | 0.0474 | 22224.6732 | 139.4157 | 44 |
| M_3_F_lph_Set_1 | 1.9139 | 0.0474 | 22217.6176 | 132.3601 | 48 |
| M_3_F_qph_Set_1 | 1.9114 | 0.0474 | 22238.0968 | 152.8392 | 57 |
| M_3_F_lqph_Set_1 | 1.9121 | 0.0474 | 22238.0968 | 152.8392 | 57 |
| M_3.1_F_l_Set_1 | 1.5874 | 0.0514 | 25306.7026 | 3221.4451 | 4 |
| M_3.1_F_q_Set_1 | 1.5021 | 0.0237 | 26865.0159 | 4779.7584 | 4 |
| M_3.1_F_p_Set_1 | 1.7159 | 0.0593 | 23278.9926 | 1193.7350 | 5 |
| M_3.1_F_h_Set_1 | 1.9094 | 0.0435 | 22239.7177 | 154.4602 | 48 |
| M_3.1_F_lq_Set_1 | 1.8705 | 0.0474 | 23098.7960 | 1013.5385 | 6 |
| M_3.1_F_lp_Set_1 | 1.7837 | 0.0553 | 23115.8632 | 1030.6056 | 7 |
| M_3.1_F_lh_Set_1 | 1.9069 | 0.0474 | 22246.1983 | 160.9407 | 50 |
| M_3.1_F_qp_Set_1 | 1.8744 | 0.0474 | 22850.7160 | 765.4585 | 8 |
| M_3.1_F_qh_Set_1 | 1.9091 | 0.0435 | 22239.7177 | 154.4602 | 48 |
| M_3.1_F_ph_Set_1 | 1.9088 | 0.0474 | 22232.1210 | 146.8635 | 51 |
| M_3.1_F_lqp_Set_1 | 1.8699 | 0.0474 | 22848.9543 | 763.6968 | 8 |
| M_3.1_F_lqh_Set_1 | 1.9088 | 0.0474 | 22246.1983 | 160.9407 | 50 |
| M_3.1_F_lph_Set_1 | 1.9086 | 0.0474 | 22225.0105 | 139.7529 | 47 |
| M_3.1_F_qph_Set_1 | 1.9107 | 0.0474 | 22234.9201 | 149.6626 | 52 |
| M_3.1_F_lqph_Set_1 | 1.9105 | 0.0474 | 22234.9201 | 149.6626 | 52 |
| M_3.2_F_l_Set_1 | 1.5743 | 0.0514 | 25307.8208 | 3222.5632 | 4 |
| M_3.2_F_q_Set_1 | 1.5036 | 0.0237 | 26865.3236 | 4780.0661 | 4 |
| M_3.2_F_p_Set_1 | 1.7079 | 0.0593 | 23291.6643 | 1206.4068 | 5 |
| M_3.2_F_h_Set_1 | 1.9066 | 0.0435 | 22252.1080 | 166.8505 | 50 |
| M_3.2_F_lq_Set_1 | 1.8754 | 0.0474 | 23118.4740 | 1033.2164 | 6 |
| M_3.2_F_lp_Set_1 | 1.7855 | 0.0553 | 23127.8457 | 1042.5882 | 7 |
| M_3.2_F_lh_Set_1 | 1.9088 | 0.0474 | 22248.5085 | 163.2509 | 48 |
| M_3.2_F_qp_Set_1 | 1.8742 | 0.0474 | 22866.5487 | 781.2912 | 8 |
| M_3.2_F_qh_Set_1 | 1.9084 | 0.0435 | 22252.1080 | 166.8505 | 50 |
| M_3.2_F_ph_Set_1 | 1.9103 | 0.0474 | 22234.0640 | 148.8065 | 49 |
| M_3.2_F_lqp_Set_1 | 1.8676 | 0.0474 | 22866.9286 | 781.6711 | 8 |
| M_3.2_F_lqh_Set_1 | 1.9097 | 0.0474 | 22248.5085 | 163.2509 | 48 |
| M_3.2_F_lph_Set_1 | 1.9095 | 0.0474 | 22227.2872 | 142.0297 | 46 |
| M_3.2_F_qph_Set_1 | 1.9112 | 0.0474 | 22244.1558 | 158.8983 | 54 |
| M_3.2_F_lqph_Set_1 | 1.9096 | 0.0474 | 22244.1558 | 158.8983 | 54 |
| M_3.3_F_l_Set_1 | 1.5862 | 0.0514 | 25308.9517 | 3223.6942 | 4 |
| M_3.3_F_q_Set_1 | 1.5007 | 0.0237 | 26865.6368 | 4780.3793 | 4 |
| M_3.3_F_p_Set_1 | 1.7212 | 0.0593 | 23304.2701 | 1219.0125 | 5 |
| M_3.3_F_h_Set_1 | 1.9066 | 0.0435 | 22268.4096 | 183.1521 | 55 |
| M_3.3_F_lq_Set_1 | 1.8688 | 0.0474 | 23136.4640 | 1051.2064 | 6 |
| M_3.3_F_lp_Set_1 | 1.7796 | 0.0553 | 23140.7512 | 1055.4937 | 7 |
| M_3.3_F_lh_Set_1 | 1.9069 | 0.0474 | 22260.7204 | 175.4629 | 51 |
| M_3.3_F_qp_Set_1 | 1.8713 | 0.0474 | 22885.3836 | 800.1261 | 8 |
| M_3.3_F_qh_Set_1 | 1.9063 | 0.0435 | 22268.4096 | 183.1521 | 55 |
| M_3.3_F_ph_Set_1 | 1.9105 | 0.0474 | 22233.6309 | 148.3733 | 46 |
| M_3.3_F_lqp_Set_1 | 1.8635 | 0.0474 | 22884.6616 | 799.4041 | 8 |
| M_3.3_F_lqh_Set_1 | 1.9075 | 0.0474 | 22260.7204 | 175.4629 | 51 |
| M_3.3_F_lph_Set_1 | 1.9106 | 0.0474 | 22233.6309 | 148.3733 | 46 |
| M_3.3_F_qph_Set_1 | 1.9070 | 0.0474 | 22245.6109 | 160.3534 | 51 |
| M_3.3_F_lqph_Set_1 | 1.9109 | 0.0474 | 22245.6109 | 160.3534 | 51 |
| M_3.4_F_l_Set_1 | 1.5814 | 0.0514 | 25310.0961 | 3224.8386 | 4 |
| M_3.4_F_q_Set_1 | 1.5019 | 0.0237 | 26865.9548 | 4780.6972 | 4 |
| M_3.4_F_p_Set_1 | 1.7050 | 0.0593 | 23316.8252 | 1231.5677 | 5 |
| M_3.4_F_h_Set_1 | 1.9073 | 0.0435 | 22262.5777 | 177.3202 | 50 |
| M_3.4_F_lq_Set_1 | 1.8666 | 0.0474 | 23156.4201 | 1071.1626 | 6 |
| M_3.4_F_lp_Set_1 | 1.7919 | 0.0553 | 23151.6214 | 1066.3638 | 7 |
| M_3.4_F_lh_Set_1 | 1.9053 | 0.0474 | 22261.2560 | 175.9985 | 48 |
| M_3.4_F_qp_Set_1 | 1.8686 | 0.0474 | 22902.0398 | 816.7822 | 8 |
| M_3.4_F_qh_Set_1 | 1.9077 | 0.0435 | 22286.9086 | 201.6510 | 60 |
| M_3.4_F_ph_Set_1 | 1.9146 | 0.0474 | 22229.9484 | 144.6909 | 43 |
| M_3.4_F_lqp_Set_1 | 1.8668 | 0.0474 | 22901.0543 | 815.7968 | 8 |
| M_3.4_F_lqh_Set_1 | 1.9047 | 0.0474 | 22280.3443 | 195.0868 | 57 |
| M_3.4_F_lph_Set_1 | 1.9105 | 0.0474 | 22229.9484 | 144.6909 | 43 |
| M_3.4_F_qph_Set_1 | 1.9066 | 0.0474 | 22243.4172 | 158.1596 | 47 |
| M_3.4_F_lqph_Set_1 | 1.9103 | 0.0474 | 22241.1508 | 155.8933 | 46 |
| M_3.5_F_l_Set_1 | 1.5747 | 0.0514 | 25311.2539 | 3225.9964 | 4 |
| M_3.5_F_q_Set_1 | 1.5021 | 0.0237 | 26866.2765 | 4781.0189 | 4 |
| M_3.5_F_p_Set_1 | 1.7010 | 0.0632 | 23329.4280 | 1244.1705 | 5 |
| M_3.5_F_h_Set_1 | 1.9076 | 0.0435 | 22260.4639 | 175.2063 | 45 |
| M_3.5_F_lq_Set_1 | 1.8632 | 0.0474 | 23175.9528 | 1090.6952 | 6 |
| M_3.5_F_lp_Set_1 | 1.7798 | 0.0553 | 23163.9855 | 1078.7280 | 7 |
| M_3.5_F_lh_Set_1 | 1.9084 | 0.0474 | 22279.2537 | 193.9961 | 53 |
| M_3.5_F_qp_Set_1 | 1.8653 | 0.0514 | 22919.0609 | 833.8034 | 8 |
| M_3.5_F_qh_Set_1 | 1.9080 | 0.0435 | 22292.4758 | 207.2183 | 59 |
| M_3.5_F_ph_Set_1 | 1.9107 | 0.0474 | 22232.4828 | 147.2252 | 40 |
| M_3.5_F_lqp_Set_1 | 1.8662 | 0.0474 | 22917.8148 | 832.5573 | 8 |
| M_3.5_F_lqh_Set_1 | 1.9047 | 0.0474 | 22293.1836 | 207.9261 | 60 |
| M_3.5_F_lph_Set_1 | 1.9095 | 0.0474 | 22232.4828 | 147.2252 | 40 |
| M_3.5_F_qph_Set_1 | 1.9116 | 0.0474 | 22250.8621 | 165.6045 | 49 |
| M_3.5_F_lqph_Set_1 | 1.9104 | 0.0474 | 22249.1681 | 163.9105 | 47 |
| M_3.6_F_l_Set_1 | 1.5772 | 0.0514 | 25312.4245 | 3227.1669 | 4 |
| M_3.6_F_q_Set_1 | 1.5037 | 0.0237 | 26864.4409 | 4779.1834 | 3 |
| M_3.6_F_p_Set_1 | 1.7144 | 0.0632 | 23341.7964 | 1256.5388 | 5 |
| M_3.6_F_h_Set_1 | 1.9064 | 0.0435 | 22279.8618 | 194.6043 | 51 |
| M_3.6_F_lq_Set_1 | 1.8540 | 0.0474 | 23194.9851 | 1109.7275 | 6 |
| M_3.6_F_lp_Set_1 | 1.7893 | 0.0553 | 23175.2335 | 1089.9759 | 7 |
| M_3.6_F_lh_Set_1 | 1.9089 | 0.0474 | 22292.7854 | 207.5278 | 56 |
| M_3.6_F_qp_Set_1 | 1.8580 | 0.0514 | 22935.6407 | 850.3832 | 8 |
| M_3.6_F_qh_Set_1 | 1.9052 | 0.0435 | 22279.8618 | 194.6043 | 51 |
| M_3.6_F_ph_Set_1 | 1.9113 | 0.0474 | 22248.0925 | 162.8349 | 44 |
| M_3.6_F_lqp_Set_1 | 1.8671 | 0.0514 | 22933.1308 | 847.8732 | 8 |
| M_3.6_F_lqh_Set_1 | 1.9048 | 0.0474 | 22292.7854 | 207.5278 | 56 |
| M_3.6_F_lph_Set_1 | 1.9103 | 0.0474 | 22248.0925 | 162.8349 | 44 |
| M_3.6_F_qph_Set_1 | 1.9105 | 0.0474 | 22234.4421 | 149.1845 | 37 |
| M_3.6_F_lqph_Set_1 | 1.9117 | 0.0474 | 22234.4421 | 149.1845 | 37 |
| M_3.7_F_l_Set_1 | 1.5870 | 0.0514 | 25313.6083 | 3228.3507 | 4 |
| M_3.7_F_q_Set_1 | 1.5027 | 0.0237 | 26864.5645 | 4779.3070 | 3 |
| M_3.7_F_p_Set_1 | 1.7027 | 0.0632 | 23354.2042 | 1268.9466 | 5 |
| M_3.7_F_h_Set_1 | 1.9084 | 0.0435 | 22287.5686 | 202.3110 | 52 |
| M_3.7_F_lq_Set_1 | 1.8691 | 0.0474 | 23214.6515 | 1129.3940 | 6 |
| M_3.7_F_lp_Set_1 | 1.7836 | 0.0553 | 23187.2279 | 1101.9704 | 7 |
| M_3.7_F_lh_Set_1 | 1.9073 | 0.0435 | 22291.2846 | 206.0270 | 53 |
| M_3.7_F_qp_Set_1 | 1.8628 | 0.0514 | 22946.4964 | 861.2389 | 7 |
| M_3.7_F_qh_Set_1 | 1.9053 | 0.0435 | 22305.2272 | 219.9697 | 60 |
| M_3.7_F_ph_Set_1 | 1.9087 | 0.0474 | 22264.6316 | 179.3741 | 47 |
| M_3.7_F_lqp_Set_1 | 1.8609 | 0.0514 | 22945.9572 | 860.6997 | 7 |
| M_3.7_F_lqh_Set_1 | 1.9060 | 0.0435 | 22306.0669 | 220.8094 | 60 |
| M_3.7_F_lph_Set_1 | 1.9100 | 0.0474 | 22264.6316 | 179.3741 | 47 |
| M_3.7_F_qph_Set_1 | 1.9102 | 0.0474 | 22270.6573 | 185.3997 | 50 |
| M_3.7_F_lqph_Set_1 | 1.9098 | 0.0474 | 22243.4532 | 158.1957 | 38 |
| M_3.8_F_l_Set_1 | 1.5879 | 0.0514 | 25314.8049 | 3229.5474 | 4 |
| M_3.8_F_q_Set_1 | 1.5028 | 0.0237 | 26864.6904 | 4779.4328 | 3 |
| M_3.8_F_p_Set_1 | 1.7054 | 0.0632 | 23366.8139 | 1281.5564 | 5 |
| M_3.8_F_h_Set_1 | 1.9074 | 0.0435 | 22303.7101 | 218.4526 | 56 |
| M_3.8_F_lq_Set_1 | 1.8687 | 0.0474 | 23233.6085 | 1148.3510 | 6 |
| M_3.8_F_lp_Set_1 | 1.7917 | 0.0553 | 23199.9227 | 1114.6652 | 8 |
| M_3.8_F_lh_Set_1 | 1.9062 | 0.0435 | 22308.6889 | 223.4314 | 57 |
| M_3.8_F_qp_Set_1 | 1.8598 | 0.0553 | 22957.0623 | 871.8048 | 7 |
| M_3.8_F_qh_Set_1 | 1.9088 | 0.0435 | 22305.4809 | 220.2234 | 56 |
| M_3.8_F_ph_Set_1 | 1.9089 | 0.0435 | 22261.2419 | 175.9844 | 44 |
| M_3.8_F_lqp_Set_1 | 1.8586 | 0.0514 | 22958.1746 | 872.9171 | 7 |
| M_3.8_F_lqh_Set_1 | 1.9054 | 0.0435 | 22309.2897 | 224.0322 | 58 |
| M_3.8_F_lph_Set_1 | 1.9108 | 0.0474 | 22261.2419 | 175.9844 | 44 |
| M_3.8_F_qph_Set_1 | 1.9088 | 0.0435 | 22273.5788 | 188.3213 | 50 |
| M_3.8_F_lqph_Set_1 | 1.9091 | 0.0435 | 22271.8249 | 186.5674 | 48 |
| M_3.9_F_l_Set_1 | 1.5865 | 0.0514 | 25316.0144 | 3230.7569 | 4 |
| M_3.9_F_q_Set_1 | 1.5030 | 0.0237 | 26864.8170 | 4779.5594 | 3 |
| M_3.9_F_p_Set_1 | 1.7161 | 0.0632 | 23378.9677 | 1293.7102 | 5 |
| M_3.9_F_h_Set_1 | 1.9093 | 0.0435 | 22305.1726 | 219.9151 | 53 |
| M_3.9_F_lq_Set_1 | 1.8577 | 0.0474 | 23252.3146 | 1167.0571 | 6 |
| M_3.9_F_lp_Set_1 | 1.7897 | 0.0553 | 23211.4570 | 1126.1994 | 8 |
| M_3.9_F_lh_Set_1 | 1.9000 | 0.0435 | 22296.6664 | 211.4089 | 49 |
| M_3.9_F_qp_Set_1 | 1.8553 | 0.0593 | 22969.3599 | 884.1024 | 7 |
| M_3.9_F_qh_Set_1 | 1.9033 | 0.0435 | 22305.1726 | 219.9151 | 53 |
| M_3.9_F_ph_Set_1 | 1.9089 | 0.0474 | 22277.6649 | 192.4074 | 49 |
| M_3.9_F_lqp_Set_1 | 1.8568 | 0.0593 | 22968.1427 | 882.8851 | 7 |
| M_3.9_F_lqh_Set_1 | 1.9041 | 0.0435 | 22296.6664 | 211.4089 | 49 |
| M_3.9_F_lph_Set_1 | 1.9082 | 0.0474 | 22277.6649 | 192.4074 | 49 |
| M_3.9_F_qph_Set_1 | 1.9053 | 0.0435 | 22272.7508 | 187.4933 | 46 |
| M_3.9_F_lqph_Set_1 | 1.9072 | 0.0435 | 22278.2145 | 192.9569 | 49 |
| M_4_F_l_Set_1 | 1.5912 | 0.0514 | 25317.2368 | 3231.9793 | 4 |
| M_4_F_q_Set_1 | 1.5032 | 0.0237 | 26864.9457 | 4779.6882 | 3 |
| M_4_F_p_Set_1 | 1.7032 | 0.0632 | 23391.2036 | 1305.9460 | 5 |
| M_4_F_h_Set_1 | 1.9036 | 0.0435 | 22294.1211 | 208.8636 | 45 |
| M_4_F_lq_Set_1 | 1.8612 | 0.0474 | 23270.9214 | 1185.6638 | 6 |
| M_4_F_lp_Set_1 | 1.7870 | 0.0553 | 23228.8601 | 1143.6026 | 8 |
| M_4_F_lh_Set_1 | 1.9054 | 0.0435 | 22302.7684 | 217.5109 | 50 |
| M_4_F_qp_Set_1 | 1.8532 | 0.0593 | 22980.3131 | 895.0556 | 7 |
| M_4_F_qh_Set_1 | 1.9053 | 0.0435 | 22294.1211 | 208.8636 | 45 |
| M_4_F_ph_Set_1 | 1.9088 | 0.0435 | 22276.2933 | 191.0358 | 45 |
| M_4_F_lqp_Set_1 | 1.8515 | 0.0593 | 22978.4392 | 893.1817 | 7 |
| M_4_F_lqh_Set_1 | 1.9071 | 0.0435 | 22302.7684 | 217.5109 | 50 |
| M_4_F_lph_Set_1 | 1.9075 | 0.0474 | 22276.2933 | 191.0358 | 45 |
| M_4_F_qph_Set_1 | 1.9064 | 0.0435 | 22267.7124 | 182.4549 | 41 |
| M_4_F_lqph_Set_1 | 1.9076 | 0.0435 | 22267.7124 | 182.4549 | 41 |

**
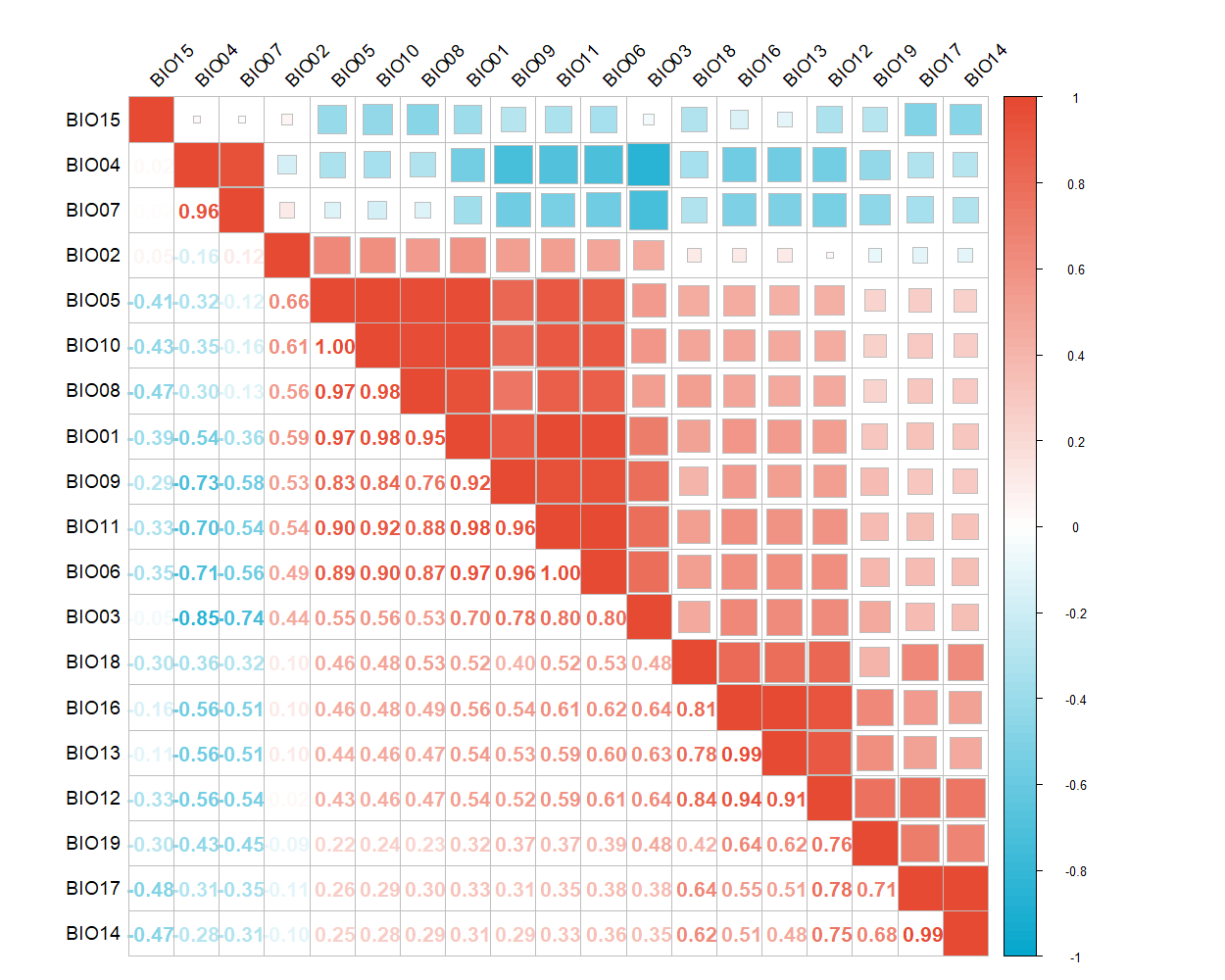
Fig.S1** Pearson's correlation coefficients between 19 environmental variables. The environment variables filtered out by the final model were: BIO04 (Temperature seasonality), BIO05 (Max temperature of warmest month) BIO13 (Precipitation of wettest month) BIO14 (Precipitation of driest month) BIO18 (Precipitation of warmest quarter).
